# First-in-Human Phase I Study of PRS-050 (Angiocal), an Anticalin Targeting and Antagonizing VEGF-A, in Patients with Advanced Solid Tumors

**DOI:** 10.1371/journal.pone.0083232

**Published:** 2013-12-13

**Authors:** Klaus Mross, Heike Richly, Richard Fischer, Dirk Scharr, Martin Büchert, Angelika Stern, Hendrik Gille, Laurent P. Audoly, Max E. Scheulen

**Affiliations:** 1 Klinik für Tumorbiologie, Albert-Ludwigs Universität, Freiburg, Germany; 2 Department of Medical Oncology, West German Cancer Center, University Hospital, University Duisburg-Essen, Essen, Germany; 3 Department of Gastroenterology, University Medical Center, Freiburg, German; 4 Magnetic Resonance Development and Application Center, University Medical Center, Freiburg, Germany; 5 Stern Consult, Basel, Switzerland; 6 Pieris AG, Freising, Germany; Schulze Center for Novel Therapeutics, Mayo Clinic, United States of America

## Abstract

**Background:**

To report the nonrandomized first-in-human phase I trial of PRS-050, a novel, rationally engineered Anticalin based on human tear lipocalin that targets and antagonizes vascular endothelial growth factor A (VEGF-A).

**Methods:**

Patients with advanced solid tumors received PRS-050 at 0.1 mg/kg to 10 mg/kg by IV in successive dosing cohorts according to the 3+3 escalation scheme. The primary end point was safety.

**Results:**

Twenty-six patients were enrolled; 25 were evaluable. Two patients experienced dose-limiting toxicity, comprising grade (G) 3 hypertension and G3 pyrexia, respectively. The maximum tolerated dose was not reached. Most commonly reported treatment-emergent adverse events (AEs) included chills (52%; G3, 4%), fatigue (52%; G3, 4%), hypertension (44%; G3, 16%), and nausea (40%, all G1/2). No anti–PRS-050 antibodies following multiple administration of the drug were detected. PRS-050 showed dose-proportional pharmacokinetics (PK), with a terminal half-life of approximately 6 days. Free VEGF-A was detectable at baseline in 9/25 patients, becoming rapidly undetectable after PRS-050 infusion for up to 3 weeks. VEGF-A/PRS-050 complex was detectable for up to 3 weeks at all dose levels, including in patients without detectable baseline-free VEGF-A. We also detected a significant reduction in circulating matrix metalloproteinase 2, suggesting this end point could be a pharmacodynamic (PD) marker of the drug’s activity.

**Conclusions:**

PRS-050, a novel Anticalin with high affinity for VEGF-A, was well-tolerated when administered at the highest dose tested, 10 mg/kg. Based on target engagement and PK/PD data, the recommended phase II dose is 5 mg/kg every 2 weeks administered as a 120-minute infusion.

**Trial Registration:**

ClinicalTrials.gov NCT01141257 http://clinicaltrials.gov/ct2/show/NCT01141257

## Introduction

Angiogenesis is a key process required for the growth and metastasis of many solid tumors and is mediated by a range of angiogenic factors, including vascular endothelial growth factor A (VEGF-A) [1]. Activation of the VEGF-A signaling pathway leads to endothelial cell proliferation, migration, and survival, as well as increased vessel permeability and mobilization of endothelial progenitor cells [2,3]. In humans, the VEGF family includes five key members, VEGF-A to VEGF-D and the placental growth factor (PlGF) [4]. The biological functions of VEGFs are mediated by binding to one or more of the related family of protein tyrosine kinase receptors (VEGFR-1, -2, and -3) [5].

Overexpression of VEGF and/or its receptors has been documented in a broad range of solid tumors [2], suggesting a potential therapeutic role for VEGF inhibitors. First proof-of-principle came when anti-VEGF antibodies were shown to inhibit the growth of several tumor cell lines in nude mice, with an associated decrease in the density of tumor blood vessels [6]. Similarly, expression of a dominant-negative version of VEGFR-2 by endothelial cells prevented glioblastoma growth in nude mice [7]. Since then, approval of bevacizumab, a humanized monoclonal antibody that neutralizes VEGF-A, as well as several small molecule tyrosine kinase inhibitors, such as sunitinib and sorafenib, which include VEGFR among their targets, have validated the use of VEGF/VEGFR-directed therapy in several oncological indications [8-11]. Other selective VEGFR-targeted agents are currently undergoing clinical evaluation in patients with advanced solid tumors, such as telatinib, vatalanib, and cediranib [12,13].

The use of monoclonal antibodies (such as bevacizumab) as targeted biological agents has been validated during the past decade through their therapeutic and commercial success. Nevertheless, they possess several practical limitations including, but not limited to, manufacturability due, in part, to their large size, posttranslational modifications of multiple polypeptide chains, and often undesired immunological effector functions. Next-generation protein scaffolds, including Anticalins, have accordingly been proposed and engineered for specific target recognition and their potential for superior development properties and therapeutic index [14].

Lipocalins are a family of structurally conserved proteins involved in diverse physiological functions. At least ten different human lipocalins have been identified to date [15], including tear lipocalin (Tlc, Lcn1), for which a range of functions has been suggested, including inactivation of viral DNA and binding of microbial siderophores [16]. 

Lipocalins with different biochemical functions share limited sequence identity, which can be less than 10% [17]. Despite the low amino acid sequence conservation and diverse binding functions of the natural lipocalins, they share a highly conserved single β-barrel ”backbone” scaffold which supports four loops of variable lengths, sequences, and conformations at its open end. This lipocalin loop region is somewhat analogous to the hypervariable complementarity-determining regions of antibodies [18,19].

Lipocalins have several biotechnological advantages over antibodies, including smaller size, being composed of a single polypeptide chain, produced in bacteria (but also eukaryotic systems if required), and possessing a simpler set of four hypervariable loops that can be more easily manipulated at the genetic level [14]. Lipocalins have been rationally engineered into Anticalins using targeted random mutagenesis and phage display selection to form novel binding proteins for specific and tight binding of low molecular weight compounds, peptides, as well as protein antigens with potential therapeutic applications [14,20]. Tlc shows broad ligand promiscuity, indicating flexibility of its binding site to accommodate a wide range of clinically relevant targets [21]. More recently, we developed an Anticalin against the cell surface tyrosine kinase receptor and signal transducer MET, further supporting the broad range of applications of the technology [22]. 

Starting from a naive combinatorial library where residues forming the natural ligand-binding site of Tlc were randomized, followed by affinity maturation, the final Anticalin PRS-050 was selected to bind all splice forms of VEGF-A with picomolar affinity. Moreover, the Anticalin was found to cross-react with the rodent orthologs [23]. As the Anticalin efficiently antagonizes the interaction between VEGF-A and its cellular receptors, inhibition of VEGF-induced mitogenic signalling and proliferation of primary human endothelial cells with subnanomolar IC_50_ values was observed *in vitro* [23]. PRS-050 was coupled with a 40 kDa polyethylene glycol (PEG) moiety in a site-directed manner to extend its half-life in plasma via a rationally engineered cysteine amino acid residue. In preclinical studies, PRS-050 has been shown to inhibit angiogenic and vascular permeability functions of VEGF, as well as exhibit potent antitumor activity in a number of settings including the A673 sarcoma model while being devoid of detectable thrombocytopenic activity [23-25]. 

Here, we present the results of a first-in-human, phase I, dose-escalation study of PRS-050 in patients with advanced solid tumors, designed to evaluate the safety, tolerability, pharmacokinetics (PK), and pharmacodynamics (PD) of this novel VEGF-A antagonist. 

## Methods

The study was reviewed and approved by the instutional ethics review board of Freiburg University (Ethik-Kommission, Albert-Ludwigs-Universität). All patients gave their signed, informed consent to participate in the study. Study period: May 28, 2010 (first patient in) to Sept. 28, 2011 (last patient out). The protocol for this trial and supporting TREND checklist are available as supporting information; see Checklist S1 and Protocol S1.

### Subjects

The trial enrolled patients aged ≥18 years with a confirmed diagnosis of advanced, recurrent, or metastatic cancer that was refractory to standard therapy, or for which there was no standard therapy available. Other inclusion criteria were measurable or nonmeasurable disease according to Response Evaluation Criteria in Solid Tumors (RECIST); Eastern Cooperative Oncology Group (ECOG) performance status ≤2; estimated life expectancy of ≥3 months; and no current acute toxicity related to previous anticancer therapy, although patients with persistent G1 and G2 toxicities induced by previous therapy were eligible. Patients were excluded if they met any of the following criteria: chronic daily treatment with aspirin (>325 mg/day), clopidogrel (>75 mg/day), or corticosteroids (≥10 mg/day methylprednisolone or equivalent), with the exception of inhaled steroids; inadequate bone marrow function, defined as absolute neutrophil count <1.5 x 10^9^/L, or platelet count <100 x 10^9^/L or hemoglobin ≤10 g/dL; inadequate liver function, defined as serum (total) bilirubin >1.5 × the upper limit of normal (ULN) and /or aspartate aminotransferase (AST) or alanine aminotransferase (ALT) >2.5 × ULN (or >5 × ULN in patients with liver metastases; inadequate renal function, defined as serum creatinine >1.5 × ULN and/or creatinine clearance <50 mL/min and/or urine dipstick for proteinuria ≥2 and >1 g of protein in 24-hour urine; lymphoma; evidence of spinal cord compression or brain metastases; uncontrolled hypertension (systolic blood pressure >150 mmHg and/or diastolic blood pressure >100 mmHg) or clinically significant cardiovascular disease; for patients not receiving anticoagulant medication, an International Normalized Ratio >1.5 or activated partial thromboplastin time (aPTT) >1.5 x ULN within 7 days before starting treatment; or history of inherited bleeding diathesis or coagulopathy with risk of bleeding.

### Trial design and treatment

In this first-in-human, phase I, open-label, dose-escalation study of PRS-050, patients with solid tumors were enrolled sequentially into cohorts with a standard 3 + 3 design. Successive cohorts received PRS-050 dose levels of 0.1, 0.5, 1.5, 3, 6, or 10 mg/kg administered intravenously (IV), initially as a slow bolus (5–20 minutes) and as a 120-minute infusion towards the end of the study in order to avoid infusion reactions (IR). A single dose was given on day 1, and safety and pharmacokinetics were assessed. Then PRS-050 was administered on days 22, 29, 36, and 43 during a repeat dosing period. From the third patient in the 3-mg/kg dose cohort onwards, prophylactic treatment with the H1 antagonist clemastin (2 mg), ranitidine (50 mg), and fortecortin (dexamethasone; 16 mg) was administered IV before the study drug.

 At baseline, patients were assessed by CT to determine eligibility. Only patients with progressive disease after the last standard therapy (if available) at baseline were included. The three study centers were located in Freiburg (2 different centers) and Essen. Recruitment was from the in-patient pool of each center as well as from out-patients referred by the local networks of medical oncologists. Patients who responded to PRS-050 treatment or had stable disease according to RESIST 1.0 criteria at day 43 were given the opportunity (at the discretion of the investigator) to receive maintenance treatment with PRS-050 given every 14 days (biweekly dosing or the biweekly phase) until tumor progression, dose-limiting toxicity (DLT) or withdrawal of patient consent. PRS-050 was initially given as a slow bolus over 3 to 5 minutes, extended to approximately 20 minutes for patients receiving PRS-050 3, 6, or 10 mg/kg, and subsequently increased to 120 minutes in patients who experienced IRs despite the use of premedication. The primary objective of the study was to evaluate the safety and tolerability of PRS-050 administered to patients with advanced solid tumors. Secondary objectives included characterization of the PD response to PRS-050, and its PK profile, as well as evaluating the efficacy of PRS-050 in terms of tumor response and exploratory biomarker readouts.

 DLT was defined as any of the following clinical toxicities according to the National Cancer Institute Common Toxicity Criteria for AEs (NCI-CTCAE version 3.0): Any grade 3 or 4 non-hematological toxicity excluding nausea, vomiting and alopecia as well as grade 4 platelet and red blood cell toxicity and grade 4 granulocyte toxicity lasting longer than 7 days.

 The maximum tolerated dose (MTD) was defined as the highest dose at which no more than one of six patients experienced a DLT, with at least two patients experiencing DLT at the next highest dose level. DLT was assessed during the follow-up period after the first dose (until day 15), and any patient with DLT either during this period or later in the study was withdrawn from treatment. 

### Assessments

 Regular safety assessments included physical examination, weight, hematology, clinical chemistry and coagulation tests, urinalysis, 12-lead electrocardiography, vital signs, and ECOG performance status. Adverse events (AEs) were monitored regularly and graded using the National Cancer Institute Common Toxicity Criteria for Adverse Events (NCI CTCAE) Version 3.0. Tumor response was assessed using RECIST [26] at baseline, on day 43, and after every fourth dose (i.e., every 8 weeks) for patients treated with biweekly PRS-050. Tumor vascularity and perfusion was assessed by dynamic contrast-enhanced magnetic resonance imaging (DCE-MRI) at baseline, day 2 and day 43. 

 MRI examinations were carried out on a clinical 1.5T MR-scanner (Symphony, Siemens, Erlangen) using body and spine array coils. A protocol using standard T1- and T2 weighted imaging was used for RECIST analysis and positioning of the succeeding dynamic scans. A T1 weighted multi TI inversion recovery TrueFISP sequence [27] was used for the DCE-MRI dynamic scan. Since a good temporal resolution is necessary to sample enough data points during the rapid contrast change after contrast agent application only one slice was acquired in 3s intervals. 110 consecutive acquisitions lead to a scan time of 5.5min. After the first 36s 0.1ml/kg of a Gd-based contrast agent (Multihance©) is automatically administered into the right antecubital vein using an MR compatible power injector (MEDRAD, Inc., Pittsburgh, PA). Software, developed under MATLAB (http://www.mathworks.com), was used for data analysis. As a model independent parameter initial area under concentration curve values for the first 60 s after the bolus reached the tissue (iAUC_60_) were calculated. The volume transfer constant between blood plasma and extravascular extracellular space (K_trans_) was evaluated by applying the tracer kinetic model of Tofts [28].

 Blood samples for PK and VEGF-A analysis were collected before the first dose on day 1, and at 0.25, 0.5, 1, 2, 4, 8, 24 (day 2), and 48 (day 3) hours after dosing, as well as days 5 (optional), 15, 22, 29, 36, and 43, and at the final visit (day 71). Additional blood samples for PK were taken 5 minutes before the end of the infusion in patients who received PRS-050 over 2 hours, and were taken before each dose in patients treated with biweekly PRS-050. Plasma concentrations of PRS-050 were measured at Covance Laboratories Ltd, Harrogate, UK, using a validated electro-chemiluminescence method. Free plasma VEGF-A and VEGF-A in complex with PRS-050 (VEGF-A/PRS-050 complex) were detected and quantified using two separate, specific electro-chemiluminescence-based immuno-assays, with analyses performed and reported by Pieris AG. The lower limit of quantification for free VEGF-A detection was 5 pg/mL and drug-target complex concentrations as low as 20 pg/mL could be quantified. Blood samples for antidrug antibody response assessment in serum were taken at baseline and on day 43, day 57 (the start of biweekly dosing) if applicable, and at the final follow-up assessment 4 weeks after completing dosing. Antidrug antibodies were analyzed using a validated direct ELISA by Covance Laboratories Ltd, Harrogate, UK. The assay reproducibly detected 25 ng/mL of a positive control rabbit antibody preparation (relative sensitivity).

 Multi-analyte profile (MAP) technology was used to analyze duplicate serum samples for the presence of potential biomarkers of response to PRS-050. Samples were taken before the first dose and at 24 (day 2) and 48 (day 3) hours after, as well as on days 5 and 15. The analysis, which is based on Luminex technology using a multiplex, microsphere-based assay, was performed by Rules-Based Medicine, Inc. (Austin, TX). 

### Statistical Analyses

The sample size was not based on statistical methodology. Decision rules for the number of patients to be treated per dose level were specified in the protocol, such that a maximum of 36 patients (six patients in each planned dosing cohort) would be treated with PRS-050. All patients receiving at least one dose of PRS-050 were included in the safety population, and all those who also had evaluable PK data were included in the PK analyses. PK parameters were derived and reported by Covance Clinical Research Unit, Leeds, UK using noncompartmental procedures in WinNonlin Version 5.2 (model 201). For analysis of the biomarker data, pairwise t-tests and Wilcoxon signed rank tests were used to compare baseline values to values at subsequent visits, for all cohorts pooled as well as for each cohort separately. The Benjamin and Hochberg procedure was used to adjust P-values for multiple comparisons. Statistical analyses were performed using the R Version 2.12 base distribution.

### PK/PD Modeling

 Findings from mouse xenograft experiments were used to predict the human dose of PRS-050 expected to be associated with antitumor activity in phase II. Antitumor activity of PRS-050 has been demonstrated in nude-mouse xenograft models: steady state trough plasma concentrations of PRS-050 were available from a U87-MG (glioblastoma) experiment, as well as more detailed PK data from a separate study done in non–tumor-bearing NMRI mice. These data were used to estimate plasma concentrations of PRS‑050 in the nude mice receiving the lowest active dose of PRS-050. Using noncompartmental PK modeling, three variables at steady state were estimated: (1) the maximum plasma concentration (C_max_), (2) the trough or minimum plasma concentration (C_min_), and (3) the average exposure over time, represented by the area under the plasma-concentration versus time curve (AUC). These values were considered to be Target Exposure Values (TEVs), expected to be associated with therapeutic activity in humans. The TEVs were 84,000 ng/mL for C_max_, 28,000 ng/mL for C_min_, and 1,200,000 hr*ng/mL for AUC, corresponding to a mean plasma concentration of 52,000 ng/mL. Single-dose K profiles were obtained for each evaluable subject participating in the current study (n=25). These data were used to estimate the dose and treatment schedule expected to result in steady state systemic PK exposure in humans comparable to the TEVs. In this study, C_max_, C_min_, and AUC were each found to increase in a linear manner with increasing dose. The correlation coefficient (R2) values were 0.87, 0.87, and 0.90 for C_max_, C_min_, and AUC, respectively. The TEVs were within the range of the plasma concentrations observed, making it reasonable to estimate the human dose associated with each of the TEVs by linear interpolation. 

## Results

**Figure 1:**
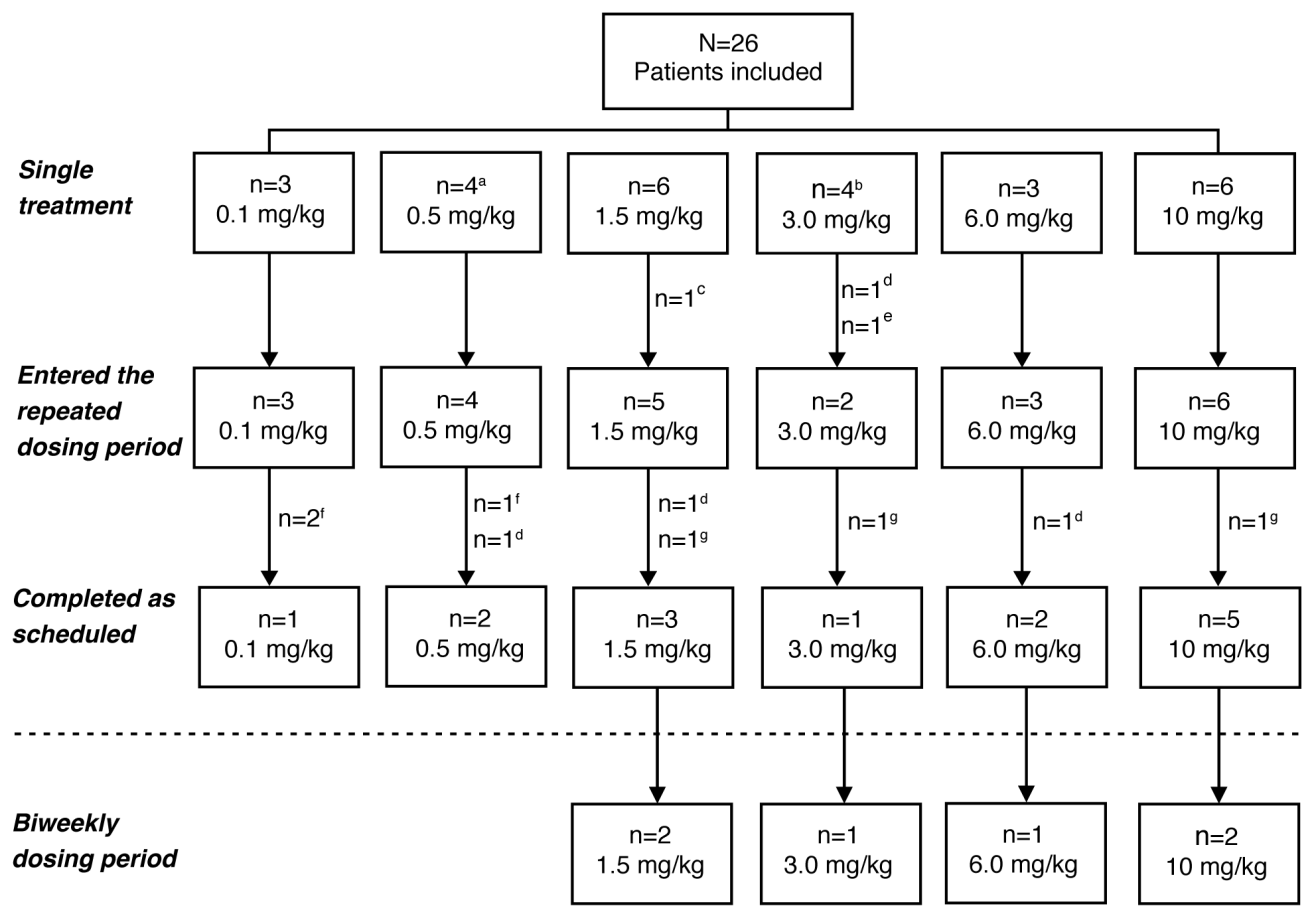
Patient disposition. Patients who completed the single treatment and repeated weekly dosing period were specified to have completed as scheduled. ^a^A fourth patient was enrolled by mistake. ^b^One patient was treated with the wrong dose of study medication. ^c^Withdrawal of consent. ^d^Disease progression. ^e^The patient erroneously treated with wrong dose of study medication was withdrawn (same patient as in footnote b). ^f^Patient went to primary care physician for further visits. ^g^Adverse event. N=total number of patients; n=number of patients in the subgroup.

Patient demographics and baseline characteristics. Twenty-six patients were enrolled, of whom 25 patients were evaluable and included in all analyses (Figure 1). Patients enrolled at each dose level were as follows: 0.1 mg/kg (n=3); 0.5 mg/kg (n=4); 1.5 mg/kg (n=6); 3.0 mg/kg (n=3); 6.0 mg/kg (n=3); and 10.0 mg/kg (n=6). Demographic and baseline characteristics are shown in Table 1.

**Table 1:** Patient demographics and baseline characteristics, all cohorts combined.

**Patient characteristic**		**Patients receiving PRS-050 (n=25)**
Gender (male/female), *n* (%)		14/11 (56/44)
Median age (range), years		62 (42–77)
ECOG performance status, *n* (%)		
	0	8 (32)
	1	16 (64)
	2	1 (4)
Tumor type, *n* (%)		
	Colorectal carcinoma	11 (44)
	Melanoma	3 (12)
	Hepatocellular carcinoma	2 (8)
	Neuroendocrine tumor	2 (8)
	Pancreatic carcinoma	2 (8)
	Other	5 (20)
Previous tumor-related treatment, *n* (%)		
	Surgery	22 (88)
	Systemic therapy	25 (100)

ECOG, Eastern Cooperative Oncology Group.

### Study Drug Exposure

 The 25 evaluable patients received a total of 141 doses of PRS-050; 16 patients received five doses or more and nine patients received fewer than five doses. Fourteen patients received five doses as scheduled, and six of these patients subsequently received biweekly dosing: two in the 1.5-mg/kg dose cohort (receiving a total of nine and ten doses, respectively); two in the 10-mg/kg dose cohort (total of eight and nine doses, respectively); and one patient each in the 3-mg/kg and 6-mg/kg dose cohorts (total of 22 and eight doses, respectively). Reasons for premature discontinuation from the study included disease progression (n=4); AE (n=3); lost to follow-up (n=3); and consent withdrawn (n=1). In a deviation to the protocol one patient in the 3 mg/kg cohort was treated with an insufficient first dose of study drug due to an incorrect body weight determination. The patient was withdrawn from the study (Figure 1).

### Safety

 One patient each in the 1.5-mg/kg and 10-mg/kg dose groups experienced a DLT, consisting of G3 hypertension and G3 pyrexia, respectively. The MTD was not reached. As shown in Table 2, the most commonly reported treatment-emergent AEs were chills (52%), fatigue (52%), hypertension (44%), and nausea (40%). IRs comprising chills (or rigors) and pyrexia were common, considered related to PRS-050, and were not observed after extending the infusion time to 2 hours in patients who initially experienced IRs despite the use of pre-medication (one patient in the 3-mg/kg dose group and three patients in the 10-mg/kg dose group, receiving a total of 17 infusions). Three patients died of disease progression during the study.

**Table 2:** Treatment-emergent AEs reported in at least 15% of patients across all cohorts, by maximum severity G^a^.

**AE**	**Number of patients (%) (n=25)**		
	**G1/2**	**G3** ^b^	**Total**
Chills^§^	12	1	13 (52)
Fatigue	12	1	13 (52)
Hypertension	7	4	11 (44)
Nausea	10	0	10 (40)
Decreased appetite	7	2	9 (36)
Pyrexia	7	1	8 (32)
Abdominal pain	6	1	7 (28)
Constipation	6	1	7 (28)
Vomiting	6	0	6 (24)
Back pain	5	0	5 (20)
Edema, peripheral	5	0	5 (20)
Hypotension	5	0	5 (20)
Tumor pain	4	1	5 (20)
Dyspepsia	4	0	4 (16)
Dyspnea	3	1	4 (16)
Flatulence	3	1	4 (16)
Weight decreased	4	0	4 (16)

^a^ Graded according to NCI CTCAE Version 3.0.

^b^ G4 events reported included ileal perforation (n=1), increased blood bilirubin (n=2), and increased uric acid and γ-glutamyltransferase (each n=1).

^§^ Infusion-related chills or rigor were not observed after extending the infusion time to 2 hours

AEs, adverse events; G, grade.

 Most treatment-emergent AEs were mild or moderate (G1/2), with the proportion of G3 AEs (11.5% of the total) remaining fairly constant across all dose levels (data not shown). Only five G4 AEs were reported, including ileal perforation (n=1), increased blood bilirubin (n=2), and increased uric acid and γ-glutamyltransferase (each n=1). In the case of the patient (from 10-mg/kg cohort) with ileal perforation, prophylactic moxifloxacin was prescribed along with concomitant clemastin, ranitidine, and dexamethasone. Following resection of the ileum segment, the patient recovered without sequelae. The ileal perforation was deemed to be probably related to PRS-050 due to the known association of this complication with bevacizumab [29].

### Immunogenicity

 PRS-050 appeared to lack immunogenicity, based on the absence of an anti-PRS-050 antibody response in 24 patients with postbaseline samples available. This included samples from six patients who received biweekly dosing. Notably, one patient was tested for ADA after having received 17 doses.

### Efficacy

 No objective tumor responses were observed. Based on RECIST criteria, 16 patients (76%) had stable disease at day 43, including one patient treated at 0.1 mg/kg, 4 patients at 0.5 mg/kg, 2 patients at each of 1.5, 3.0, and 6.0 mg/kg, and 5 patients at 10 mg/kg. Based on investigator assessment, seven patients had stable disease, including two patients each in the 3.0- and 10-mg/kg dose groups and one patient each in the 0.1-, 1.5-, and 6-mg/kg dose groups, respectively. Five of these patients started biweekly dosing, and had a median duration of stable disease of 3.2 months (range, 2.7–9.6 months). 

 Owing to the small number of patients per dose group, no trend between increasing PRS-050 dose and tumor vascularity and perfusion (as measured by DCE-MRI) was apparent. The largest decrease in K_trans_ and iAUC_60_ (DCE-MRI parameters) between screening and day 43 was seen in the 10-mg/kg dose group.

### Pharmacokinetics

Summary PK parameters of PRS-050 were prepared based on data from all 25 patients after a single IV administration of 0.1 mg/kg to 10.0 mg/kg PRS-050, and are shown in Table 3. Peak plasma concentrations were reached 0.25- to 0.5-hours postdose across all cohorts and declined in a biphasic manner, with a mean terminal half-life (t_½_) ranging from 5.5 to 7.0 days at doses of 0.5 mg/kg or greater. In the 0.1-mg/kg dosing cohort, mean t_½_ was 3.2 days, and showed high inter-patient variability as reflected by the high geometric coefficient of variation (CV) of 87%. However, PRS-050 was below the lower limit of detection by days 15 and 22, respectively, in two of the three patients from this cohort, and the PK parameters may, therefore, be unreliable in this cohort. Exposure to PRS-050 (as measured by C_max_, AUC_0-tlast_, and AUC_0-∞_) increased in a dose-proportional manner over the dose range examined. Clearance and volume of distribution of PRS-050 were generally independent of dose, supporting the presence of dose-proportional PK. Clearance ranged from 0.0032 to 0.00730 mL/min/kg, reflecting the relatively slow catabolism process typical for proteins. The volume of distribution ranged from 0.044 to 0.061 L/kg, encompassing human plasma volume (approximately 0.043 L/kg) and suggesting that PRS-050 was mainly confined to the systemic circulation with some binding/distribution to tissue. Interpatient variability was moderate, as measured by pooled geometric mean CV, with values of 39% and 44% for C_max_ and AUC_0-∞_, respectively. 

**Table 3:** Summary of mean PK parameters of PRS-050 by dosing cohort.

		**PRS-050 dose (mg/kg)**					
Parameter*		**0.1 (n=3)**	**0.5 (n=4)**	**1.5 (n=6)**	**3.0 (n=3)**	**6.0 (n=3)**	**10.0 (n=6)**
After a single IV administration							
	AUC_0-last_, μg.h/mL	184 (119)	1980 (25.5)	4370 (39.7)	11,800 (34.6)	26,900 (44.8)	34,500 (33.5)
	AUC_0-∞,_ μg.h/Ml	227 (83.9)	2160 (24.5)	4880 (39.1)	14,300 (46.3)	31,200 (50.1)	38,100 (33.6)
	C_max_, μg/mL	4.2 (34.3)	22.9 (21.0)	49.1 (17.7)	102.0 (56.6)	214.0 (49.7)	327.0 (51.1)
	C’_168h_, μg/mL	0.3 (273)	4.8 (26.0)	10.4 (38.3)	28.9 (58.8)	63.5 (42.9)	79.2 (38.0)
	T_max_, h	0.32 (0.27–0.55)	0.31 (0.27–0.35)	0.31 (0.25–0.55)	0.25 (0.25–0.37)	0.47 (0.33–0.48)	0.52 (0.27–0.60)
	t_1/2_, days	3.2 (87.0)	5.5 (28.0)	6.0 (15.5)	6.8 (37.9)	7.0 (19.8)	6.3 (8.9)
	CL, mL/min/kg	0.0073 (83.9)	0.0039 (24.5)	0.0051 (39.1)	0.0035 (46.3)	0.0032 (50.1)	0.0044 (33.6)
	Vz, L/kg	0.049 (16.8)	0.044 (36.8)	0.064 (29.6)	0.049 (48.4)	0.047 (37.1)	0.057 (36.1)
	Vss, L/kg	0.048 (15.8)	0.044 (35.1)	0.061 (28.7)	0.050 (48.0)	0.046 (38.1)	0.055 (35.1)
After repeated once weekly IV administration							
	Ctrough, ss, ng/mL	900 (52.2)	6910 (29.4)	17,900 (72.2)	44,900; 94,800^†^	93,100 (76.6)	107,000 (58.2)
	RA_Ctrough_	2.67 (126)	1.46 (30.7)	1.46 (44.6)^§^	1.79, 1.82^†^	1.47 (34.2)	1.35 (59.3)

* Geometric mean values (coefficient of variation; CV%) presented except for T_max_ where median (range) presented.

^†^ n=2.

^§^ n=3.

AUC, area under the plasma-concentration versus time curve; CL, clearance; C_max_, maximum plasma concentration; C_min_, trough or minimum plasma concentration; IV, intravenous; PK, pharmacokinetic; RA_Ctrough_, accumulation ratio based on trough concentrations; t_1/2_, terminal half-life; Vss, volume of distribution at steady state; Vz, volume of distribution during the terminal phase.

 Inspection of C_trough_ concentrations of PRS-050 after repeated dosing suggests that PRS-050 PK were at or approaching steady state conditions before dosing on day 43. Accumulation of PRS-050 following repeated weekly IV administration was assessed using the ratio of C_trough_ at steady state (day 43) and the predicted concentration 168 hours after a single IV dose (C’168h). With the exception of the lowest dose group (0.1 mg/kg), mean accumulation of PRS-050 was less than two-fold (Table 3), although it is possible that steady state conditions had not been fully met. 

### Pharmacodynamics

**Figure 2:**
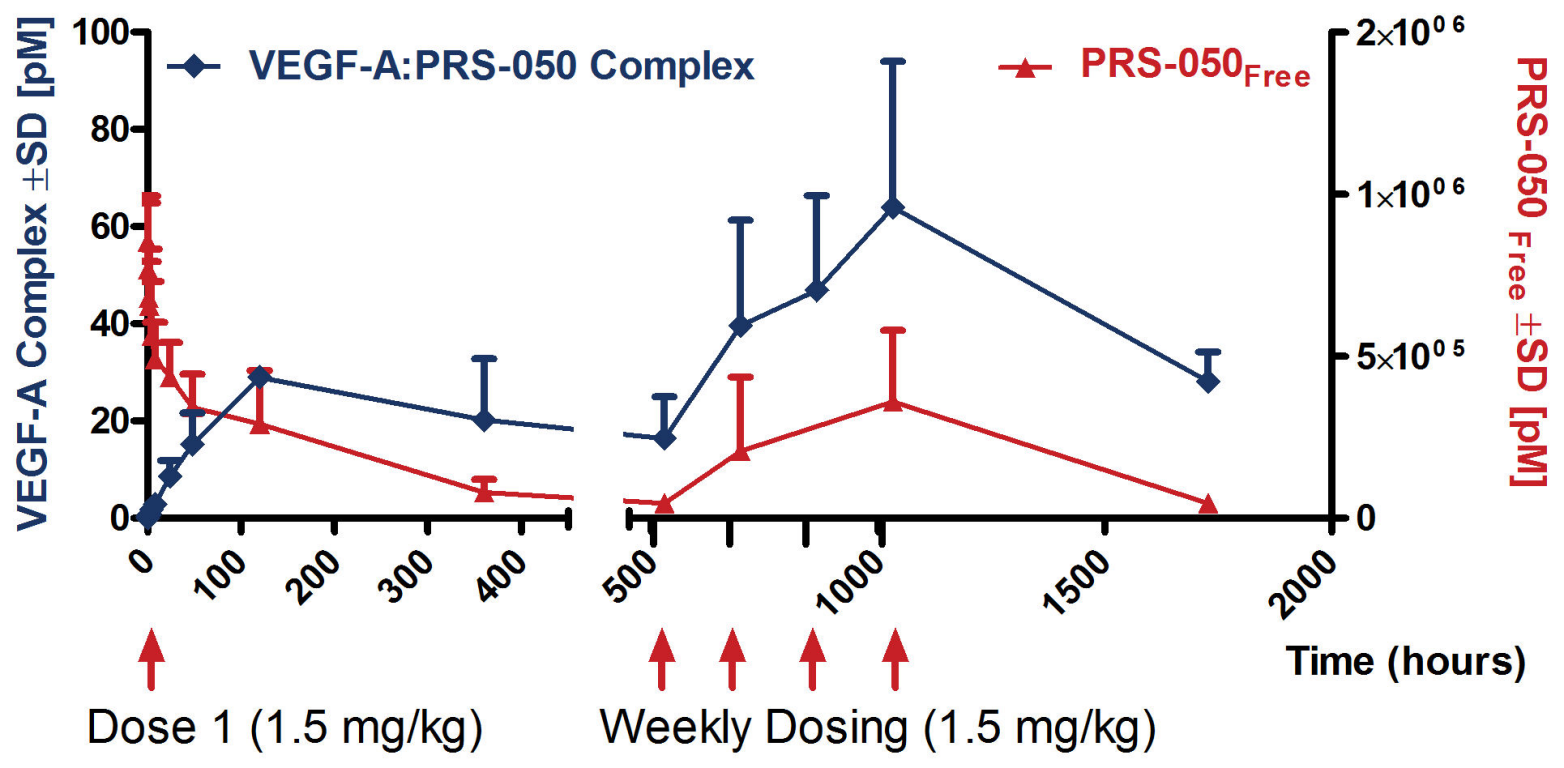
Free PRS-050 drug levels are in excess of VEGF-A/PRS-050 complex concentrations. Molar plasma concentrations of unbound PRS-050 (red, right Y-axis) and VEGF-A/PRS-050 complex (blue, left Y-axis) in patients treated with 1.5 mg/kg PRS-050 (*n* = 6) ±SD.

To assess engagement of PRS-050 with its target, plasma levels of free VEGF-A and of the VEGF-A/PRS-050 complex were measured using two separate assays. Samples for analysis were available from all 25 patients. Free VEGF-A was detected in predose samples from nine patients, but was undetectable after PRS-050 dosing at levels of 0.5 mg/kg and above, and remained below the lower limit of quantification (5 pg/mL) over the following 3-week observation period in 21/25 patients (84%). The four remaining patients were all among those with detectable free VEGF-A at baseline; in three of these patients, treated with 1.5-, 6.0-, and 10.0-mg/kg PRS-050, respectively, free VEGF-A was detectable only sporadically or right at the end of the observation period. In the fourth patient, who received 0.1-mg/kg PRS-050, free VEGF-A was undetectable immediately after dosing but was detected reliably from 24 hours onwards after dosing. These observations confirm the lack of circulating unbound VEGF-A activity in the majority of patients treated with PRS-050 and suggest target saturation. Consistent with this, the VEGF-A/PRS-050 complex became detectable in all 25 patients immediately after dosing, with levels initially rising over time. The complex remained detectable in all available samples from all patients, apart from one patient (treated with 0.5 mg/kg) in whom no complex was detected at the final visit on day 71. Figure 2 shows plasma levels of the VEGF-A/PRS-050 complex alongside those of unbound PRS-050 for patients in the 1.5-mg/kg cohort, demonstrating that PRS-050 was in significant molar excess over the complex at all times, and again consistent with target saturation.

**Figure 3:**
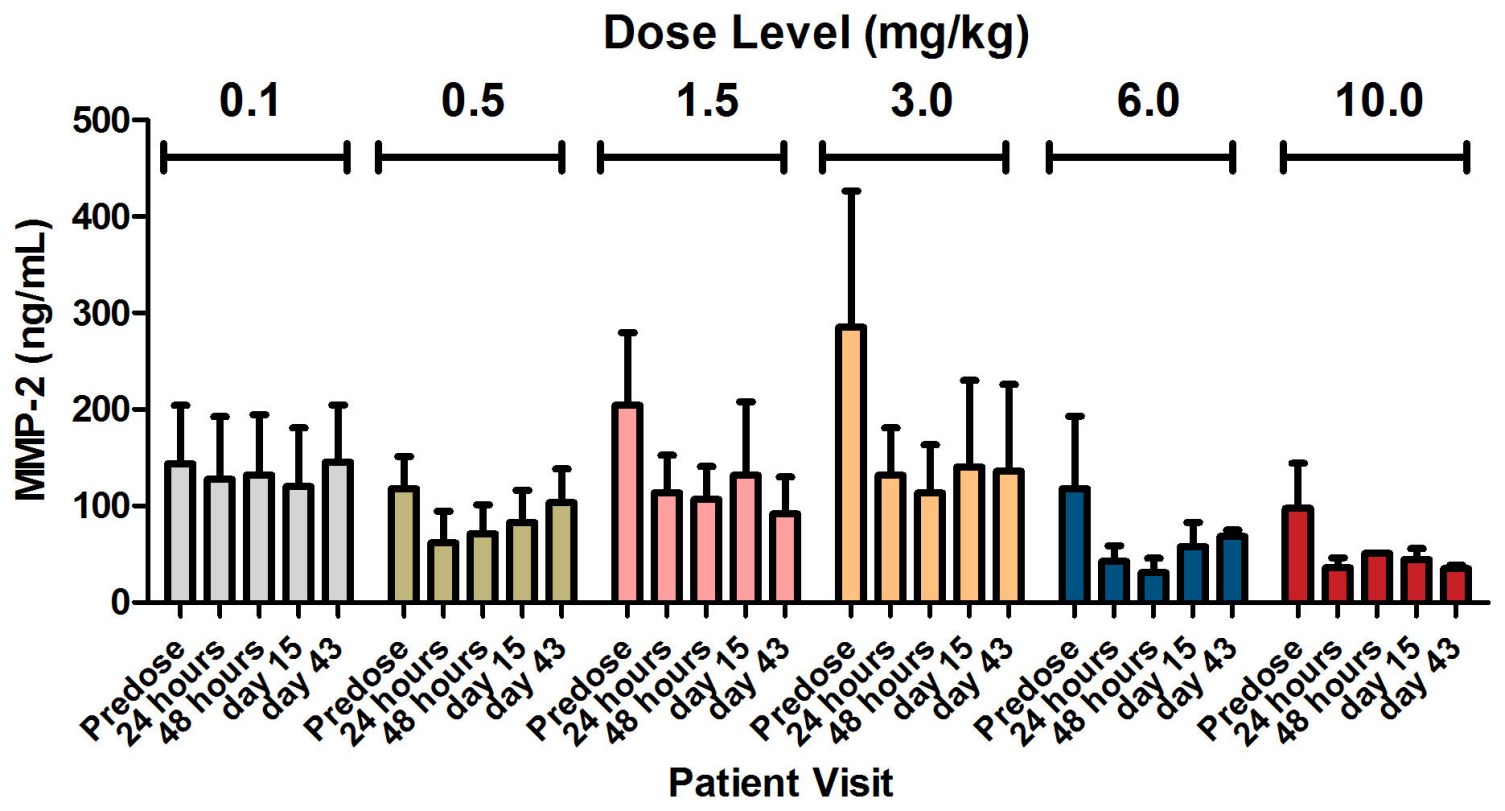
Changes in level of serum MMP-2 after treatment with a single dose of PRS-050. Note that dose levels are depicted according to dosing cohort (±SE).

 A total of 133 human serum samples, taken before the first dose and at days 2, 3, 5, and 15, were analyzed using MAP technology for changes in levels of 101 potential cancer-related biomarkers on treatment with PRS-050. The levels of 21 markers changed significantly between baseline and time points after treatment, including down-regulation of free VEGF-A, consistent with the above results, and down-regulation of matrix metalloproteinase 2 (MMP-2) at doses of 0.5-mg/kg PRS-050 and above (p<0.01; Figure 3). Although these data provide a novel observation to the best of our knowledge, further validation in humans is needed.

## Discussion

This is the first report of an Anticalin (Tlc-derived) administered in humans. In this phase I, dose-escalation study in patients with advanced solid tumors, PRS-050 was generally well-tolerated when administered as a 2-hour infusion at doses of up to 10 mg/kg and exhibited dose-proportional PK while demonstrating expected PD effects. Based on the absence of an antidrug antibody response across all dose cohorts, PRS-050 was deemed to be nonimmunogenic. The half-life of PRS-050 was approximately 6 days, with less than two-fold accumulation on weekly dosing. The MTD was not achieved.

 In order to assist with predicting effective doses of PRS-050 for further clinical studies and in analogy to a published approach [30], PK/PD modeling using the human PK parameters generated in this study was performed. Different treatment schedules were simulated in order to estimate the doses and dosing interval required to achieve human target exposure values (TEVs) for C_max_, Cmin and AUC, associated with activity in preclinical mouse models. On the basis of the estimates shown in Table 4, the recommended phase II dose for PRS-050 was 6 mg/kg infused IV over 2 hours every 2 weeks.

**Table 4:** IV doses (mg/kg) at which the average subject would be expected to reach TEVs.

	**Schedule**		
	**Weekly**	**Every 14 days**	**Every 21 days**
C_max_	2.3	3.3	3.8
C_min_	1.7	5.1	10.8
AUC	2.1	4.5	6.2

AUC, area under the plasma-concentration versus time curve; C_max_, maximum plasma concentration; C_min_, trough or minimum plasma concentration; IV, intravenous; TEVs, target exposure values.

The most common treatment-related AEs reported were chills and fatigue (13 patients (52%) for each event), which were predominantly mild or moderate (G1/2) in nature. Infusion-related reactions, comprising pyrexia and chills (predominantly G1/2), were reported in some patients when PRS-050 was administered over 5-20 minutes. These IRs usually responded well to steroids or analgesics, with or without H1 and H2 blockers. No further reactions were reported in a small group of patients after extending the infusion time to 2 hours together with prophylactic treatment, suggesting the main cause of IRs was the speed of administration. Confirmation of the absence of reactions to PRS-050 in a larger group of patients will be determined using an infusion period used for other licensed biologics in oncology. IRs have been documented with other anticancer treatments, such as monoclonal antibodies bevacizumab and cetuximab, which are infused over 30 minutes or longer [31].

Hypertension was observed in 11 patients (44%) and was G3 in four cases (16%). Hypertension is an expected, on-target effect of VEGF-inhibition which has been reported in up to 80% of patients treated with various inhibitors of the VEGFR signaling pathway [32-35], and which has also been shown to be a biomarker for response in this class of drugs [33,36-38]. In a randomized phase II trial of bevacizumab in patients with metastatic renal-cell carcinoma, dose-dependent development of hypertension was observed, and a significant number of patients (21%) treated with high-dose bevacizumab experienced G3 hypertension compared with placebo (p≤0.05) [39]. In phase I studies of selective small molecular inhibitors of VEGFR, telatinib, vatalanib, and cediranib in patients with advanced solid tumors, 19% to 28% of patients experienced G3/4 hypertension [12,13]. Furthermore, multitargeted kinase inhibitors, sorafenib and sunitinib, have been associated with the development of a preeclampsia-like syndrome, characterized by hypertension and proteinuria [40]. 

The cause of hypertension may be related to the normal function of VEGF in stimulating production of mediators of vasodilation, nitric oxide (NO) and prostacyclin (PGI2) in endothelial cells, via VEGFR-mediated signaling [41-43]. A lack of NO and PGI2 causes an increase in peripheral vascular resistance and also an increase in blood pressure. Another possible cause of hypertension is reduction in the density of capillaries in a tissue (capillary rarefaction), demonstrated in both preclinical models and humans following chronic VEGF-A inhibition) [34].

It was noted that one patient in the 10-mg/kg dose cohort experienced ileal perforation, which was subsequently managed with no further complications. While it is known that bowel perforations are a known risk factor associated with the use of bevacizumab [29], it cannot be known for certain whether this isolated case of ileal perforation was as a result of PRS-050. Further evidence is required from a larger group of patients.

Importantly, this study demonstrated that PRS-050 engages with and saturates its target, VEGF-A. Free VEGF-A was undetectable in all patients immediately after dosing and remained so in the vast majority of patients throughout the observation period. The VEGF-A/PRS-050 complex was detected in all patients immediately after dosing and was present throughout the available observation period in 24 of 25 patients, providing direct evidence of target engagement. An initial rise in complex levels, as well as its persistence in the circulation, may reflect restricted clearance of the complex compared with free VEGF-A; a hypoxia-driven increase in VEGF-A synthesis rate may also have contributed to the increased levels with time. Comparison of plasma levels of the VEGF-A/PRS-050 complex with those of total plasma PRS-050 showed that the drug remained in significant molar excess of the complex at all times, adding credence to the conclusion that VEGF-A was saturated. At the recommended phase 2 dosing regimen derived from PK/PD modeling (6 mg/kg infused over 2 hours every second week) complete target saturation is therefore also to be expected.

It is known that plasma concentration of VEGF-A can be elevated in response to inhibition of VEGFR with tyrosine kinase inhibitors such as vatalanib, vandetanib and regorafenib [12,44-46]. The ability of PRS-050 to immediately saturate VEGF-A makes it a potentially synergistic partner for VEGFR inhibitors. However, caution must be advised owing to the reported increased toxicity of bevacizumab in combination with inhibitors of VEGFR [47,48].

Analysis of potential biomarkers in this study was difficult to interpret, given the small sample size. Nonetheless, we report a significant decrease in levels of MMP-2 after treatment with PRS-050 at doses of 0.5 mg/kg and above, suggesting a novel link in humans between MMP-2 and blockade of VEGF-A signaling. We suggest that this may be directly linked to the decreased availability of free VEGF-A, supported by evidence that VEGF upregulates the expression of MMP-2 [49,50]. The role of MMPs in tumor progression and invasion, as well as in earlier stages such as in malignant transformation, angiogenesis, and tumor growth, has been well-documented [51]. In particular, MMP-2 and MMP-9 (and possibly MMP-1) have been shown to have a central role in initiating angiogenesis [52-55], and may do so by mobilizing VEGF-A [52,56,57]. It is unclear, however, how these observations are consistent with those in this study: that decreased VEGF-A activity has a negative impact on MMP-2. Nonetheless, if the two parameters are linked, it is possible that MMP-2 levels may serve as a PD biomarker for PRS-050 target binding.

In contrast to monoclonal antibodies, PRS-050 is produced more easily in E. coli. Another important distinction is the absence of an Fc domain in this protein scaffold. Bevacizumab is generally well tolerated while significant toxicities occur in a subset of patients, for example thromboembolic events [58]. It has been demonstrated that bevacizumab forms multimeric immune complexes which may be a cause for these effects [59]. Furthermore, immune complex deposition in glomeruli of the kidney may cause glomerulosclerosis [60].

With respect to thromboembolic complications of bevacizumab use, these can be mirrored in human FcγIIa receptor transgenic mice where administration of complexes between heparin, bevacizumab and heparin-binding isoforms of VEGF-A lead to platelet aggregation and thrombosis [61]. Preclinically, PRS-050 which lacks an Fc domain has not exhibited the thrombocytopenic activity exhibited by bevacizumab in human Fc receptor transgenic mice [25]. However this parameter will require evaluation in a prospective larger clinical trial to investigate whether it translates into a safety benefit in humans.

In summary, this phase I study showed that PRS-050 could be safely given to patients with advanced solid tumors at doses up to 10 mg/kg. PD studies showed that PRS-050 successfully bound to and saturated its target VEGF-A for up to 3 weeks after a single dose and produced no immunogenic activity. The recommended phase II dose is 6 mg/kg infused over 2 hours, given every 2 weeks.

## Supporting Information

Checklist S1 **TREND Checklist.**
(PDF)

Protocol S1 **Trial Protocol.**
(PDF)
