# Supplementary material for: First-in-Human Phase I Study of PRS-050 (Angiocal), an Anticalin Targeting and Antagonizing VEGF-A, in Patients with Advanced Solid Tumors
Source: PLoS One. 2013 Dec 13;8(12):e83232. doi: 10.1371/journal.pone.0083232 (PMC3862718; doi:10.1371/journal.pone.0083232)
Supplement: Protocol S1 — Trial Protocol. (PDF) [file pone.0083232.s002.pdf]

CLINICAL STUDY PROTOCOL

A PHASE I, OPEN-LABEL STUDY OF THE SAFETY, TOLERABILITY, AND PHARMACOKINETICS OF ANGIOCAL® (PRS-050-PEG40) IN PATIENTS WITH SOLID TUMORS

Protocol code:	Pieris001
EudraCT No.:	2009-015601-38
Clinical study phase:	I
Investigational product:	Angiocal® (PRS-050-PEG40)
Indication:	Solid tumors
Sponsor:	Pieris AG<br>Lise-Meitner-Straße 30<br>85354 Freising<br>Germany
Protocol version:	Final 2.0
Date:	10-May-2010

This study protocol must be kept strictly confidential. Disclosure of the contents (in whole or part) to third parties is permissible only with written consent of Pieris AG.

1 Summary and flow chart

Study code: Pieris001

Title of the study:

A phase I, open-label study of the safety, tolerability, and pharmacokinetics of Angiocal® (PRS-050-PEG40) in patients with solid tumors

Coordinating investigator:

PD Dr. med. Klaus Mross, Tumor Biology Center at the Albert-Ludwigs-University Freiburg, Department of Medical Oncology, Breisacher Str. 117, 76106 Freiburg im Breisgau, Germany

Countries and centers: 3 centers in Germany

Clinical phase: I

Study duration (planned): approximately 9 to 12 months

Study design: Open-label, multi-center, dose escalation study

Methodology:

This is a phase I, open-label, dose escalation study with Angiocal® in patients with solid tumors (cancer patients excluding patients with lymphomas) using a group sequential adaptive treatment assignment. Patients will be allocated to different dose levels in small cohorts and will receive a total of 5 intravenous (i.v.) applications of Angiocal®: one single dose application on Day 1 and one application once weekly for 4 weeks during a repeated dosing period. The single dose application will be followed by a 3-week follow-up period prior to start of the repeated dosing period. The time between the single dose and the repeated dosing period may be adjusted should the plasma half-life of the drug differ from the predicted duration based on pre-clinical studies. Patients will be evaluated at Screening (Day -7 to 0) and Baseline (Day 1). Follow-up visits will be performed on Days 2, 3, 5 (optional), and 15 after the single dose application. In the absence of any dose limiting toxicity (DLT) and based on the patient's and investigator's decision, patients will continue into the repeated dosing period, where they will be treated and evaluated once weekly for 4 weeks on Days 22, 29, 36, and 43[1]. A Final Visit will be performed on Day 71. For patients who discontinue after the follow-up period and do not continue into the repeated dosing period, assessments scheduled for Day 22 and Day 29 should be performed whenever possible. Safety will be

[1] At Day 43, patients who respond to Angiocal® treatment or have stable disease will, at the discretion of the investigator, be given the opportunity to get further treatment with Angiocal® in a biweekly schedule. The Final Visit at Day 71 will not be performed in these patients.

assessed by the evaluation of adverse events (AEs), laboratory parameters, physical examination, electrocardiogram (ECG), Anti-Drug Antibody (ADA) response and vital signs. Efficacy will be assessed by Eastern Cooperative Oncology Group (ECOG) performance status, by the assessment of a tumor-associated biomarker, as tumor response measured by RECIST (Response Evaluation Criteria In Solid Tumors) criteria with computed tomography (CT) or magnetic resonance imaging (MRI) and by the measurement of tumor vascularity and perfusion with DCE-MRI (Dynamic Contrast-Enhanced MRI). Concentrations of Angiocal® in plasma will be measured to calculate standard pharmacokinetic parameters and to determine the plasma half-life for adjusting the time between the single and repeated dosing period.

Study objectives:

Primary objective

- To evaluate the safety and tolerability of Angiocal® (PRS-050-PEG40) when administered intravenously as a slow bolus injection to patients with solid tumors.

Secondary objectives

- To characterize the pharmacodynamic response and evaluate the pharmacokinetic profile of Angiocal® (PRS-050-PEG40);
- To evaluate the effectiveness of Angiocal® (PRS-050-PEG40) in terms of tumor response.

Treatments:

Dosage form:	Solution for i.v. injection
Active ingredient:	PRS-050-PEG40
Inactive ingredient:	Phosphate buffered saline (PBS)
Doses:	0.1 mg/kg, 0.5 mg/kg, 1.5 mg/kg, 3 mg/kg, 6 mg/kg, 10 mg/kg and 15 mg/kg. Increments to the next higher dose level may be reduced at the discretion of the investigator and in accordance with the sponsor.
Frequency:	Five applications: single application (Day 1) and repeated applications once weekly for 4 weeks (Days 22, 29, 36, and 43). Further applications may be given biweekly (every 2 weeks) in case of stable disease or response to treatment.

Dose escalation:

Patients will be allocated to a dose level of Angiocal® in small cohorts of three patients. The first cohort will be allocated to the 0.1 mg/kg dose level. Within a cohort, treatment will occur sequentially: only one patient will be treated first, the next two patients will be treated after 48 hours of follow-up (Day 3) of the previously treated patient. The dose will be increased to the next higher level when Day 15 data of all three patients of one cohort are available and are reviewed. Treatment of subsequent cohorts will depend on the occurrence of DLT of the previous cohort. The maximum dose level studied will be 15 mg/kg; however, the maximum dose may be decreased based on the judgment of the investigator and sponsor. In case of any arising safety issues dose increments to the next higher dose level may be reduced at the discretion of the investigator and in accordance with the sponsor.

Dose limiting toxicity:

DLT is defined as any of the following clinical toxicities, referencing National Cancer Institute Common Toxicity Criteria for AEs (NCI-CTCAE version 3.0):

Non-hematological toxicity

- Any grade 3 or 4 toxicity, excluding nausea and vomiting, which responds to antiemetic treatment and alopecia;
- Prolonged (>2 weeks) grade 2 toxicities including serum creatinine 1.5 - 3 x upper limit of normal (ULN) or calculated creatinine clearance <60 mL/min, increase in alanine aminotransferase (ALT), aspartate aminotransferase (AST), alkaline phosphatase >2.5 - 5.0 x ULN, total bilirubin >1.5 - 3.0 ULN, intention tremor, slurred speech, nystagamus, or dysmetria. Exception: patients with liver metastases at screening must have a (>2 weeks) grade 3 toxicity with an increase in ALT and AST >5.0 - 20.0 x ULN.

Hematological toxicity

- Grade 4 platelets and red blood cell toxicities,
- Grade 4 granulocyte toxicity lasting ≥ 7 days;
- Febrile neutropenia defined as an absolute neutrophils count <500/mm^3 together with fever either as 2 events of increased oral temperature (>38°C) with one hour interval or a single event of oral temperature (>38.5°C), provided that this single episode is not clearly related to other events.

Those events will be indicated as DLT if they occur after the single dose application within the follow-up period (until Day 15). Patients who experience a DLT after the single dose application within the follow-up period will not continue into the repeated dosing period. Patients who experience one of those events during the repeated dosing period will be withdrawn from the study.

Number of patients planned:

At first, patients will be allocated to a dose level of Angiocal® in small cohorts of three patients. The number of patients treated per cohort will be adjusted according to the occurrence of a DLT:

- If **none** of the patients in a cohort experiences a DLT, then the next cohort will receive the next higher dose (if current dose ≤15 mg/kg).
- If **one** patient in a cohort experiences a DLT, then 3 more patients will be allocated to the same dose level:
    - If none of these patients experiences a DLT, then the next cohort will receive the next higher dose (if current dose ≤15 mg/kg);
    - If one or more of these three patients experiences a DLT, then dose escalation will stop. If the current dose is >0.1 mg/kg the prior lower dose will be the maximum tolerated dose (MTD) and three (3) additional patients will be treated with the highest dose below the MTD, if only three patients were treated previously at that dose.
- If **two** or more patients in a cohort experience a DLT dose escalation will stop. If the current dose is >0.1 mg/kg the previous lower dose will be the MTD and three (3) additional patients will be treated with the highest dose below the MTD, if only three patients were treated previously at that dose.

A maximum of 42 patients will be treated with Angiocal®, given that at each dose level one of three patients presents with a DLT and for the 2nd set of three patients at this dose level no DLT is observed.

Patient population:

Inclusion criteria:

1. Males or females with pathologically confirmed diagnosis of advanced, recurrent or metastatic cancer, refractory to standard therapy. Patients with tumors for which no standard therapy exists will also be eligible;
2. Age ≥18 years;
3. Measurable or non-measurable disease according to RECIST, as determined by the investigator;
4. ECOG performance status ≤2;
5. Estimated life expectancy of at least 3 months;
6. No current toxicities due to previous anticancer therapy (radiation therapy, chemotherapy, or surgery);
7. Signed informed consent form and ability to understand the study procedures.

Exclusion criteria:

1. Concomitant anticancer therapy, including radiation;
2. Current or previous (within 30 days of first study dosing) treatment with another investigational drug or participation in another clinical study;
3. Chronic daily treatment with aspirin (>325 mg/day) or clopidogrel (>75 mg/day);
4. Chronic daily treatment with corticosteroids (dose of ≥10 mg/day methylprednisolone or equivalent), with the exception of inhaled steroids;
5. Inadequate bone marrow function: absolute neutrophil count (ANC): <1.5 x 10^9/L, or platelet count <100 x 10^9/L or hemoglobin ≤10 g/dL;
6. Inadequate liver function, defined as:
   - Serum (total) bilirubin >1.5 x ULN, and/or
   - AST or ALT >2.5 x ULN (>5 x ULN in patients with liver metastases);
7. Inadequate renal function, defined as:
   - Serum creatinine >1.5 x ULN, and/or
   - Creatinine clearance <50 mL/min (calculated according to Cockroft and Gault), and/or
   - Urine dipstick for proteinuria ≥2+ and >1 g of protein in the 24-hour urine (patients discovered to have ≥2+ proteinuria on dipstick urinalysis have to undergo a 24 hour urine collection and demonstrate ≤1 g of protein in the urine);
8. Patients not receiving anticoagulant medication who have an International Normalized Ratio (INR) >1.5 or an activated partial thromboplastin time (aPTT) >1.5 x ULN within 7 days prior to first study treatment. Note: Patients receiving full dose oral or parenteral anticoagulants may be included in the study as long as anticoagulant dosing has been stable for at least 2 weeks prior to study entry and the appropriate coagulation monitoring tests are within local therapeutic limits;
9. Patients with lymphomas;
10. Evidence of spinal cord compression or brain metastases. A CT or MRI of the brain must be performed if the presence of metastases at these sites is suspected (CT or MRI scans performed within 4 weeks prior to the first study treatment may be sufficient);
11. Other malignancy diagnosed within the previous 5 years, except for adequately treated carcinoma *in situ* of the cervix or squamous carcinoma of the skin, or adequately controlled limited basal cell skin cancer;
12. Pregnant or lactating females. Serum pregnancy test must be performed within 7 days prior to study treatment start, or within 14 days with a confirmatory urine pregnancy test within 7 days prior to study treatment start;
13. All patients (including both female patients of childbearing potential and male patients with childbearing potential partners) who do not use a highly effective method of birth control (failure rate less than 1% per year when used consistently and correctly), e.g. implants, injectables, combined oral contraceptives in combination with a barrier

method, some intrauterine contraceptive devices, sexual abstinence, or a vasectomized partner;

14. Major surgical procedure (including open biopsy) within 28 days prior to the first study treatment, or anticipation of the need for major surgery during the course of the study treatment;
15. Minor surgical procedures, within 24 hours prior to the first study treatment;
16. History or evidence of inherited bleeding diathesis or coagulopathy with the risk of bleeding;
17. Uncontrolled hypertension (systolic blood pressure >150 mmHg and/or diastolic blood pressure >100 mmHg) or clinically significant (i.e. active) cardiovascular disease: cerebrovascular accident/stroke within ≤6 months prior to the first study treatment, myocardial infarction within ≤6 months prior to the first study treatment, unstable angina, New York Heart Association (NYHA) Grade II or greater congestive heart failure, or serious cardiac arrhythmia requiring medication;
18. History of abdominal fistula, grade 4 bowel obstruction or gastrointestinal perforation, intra-abdominal abscess within 6 months of enrollment;
19. Lung carcinoma of squamous cell histology or any histology in close proximity to a major vessel;
20. Serious non-healing wound, peptic ulcer or bone fracture;
21. Known hypersensitivity to the study medication or any of its excipients;
22. Evidence of any other medical conditions (such as psychiatric illness, physical examination or laboratory findings) that may interfere with the planned treatment, affect patient compliance or place the patient at high risk of treatment-related complications;
23. Previous enrollment in this study;
24. Known hepatitis B or C or HIV infection;
25. Employees of the sponsor or patients who are employees or relatives of the investigator.

Criteria for evaluation:

Safety

- AEs/toxicity (CTC AE v3.0);
- Laboratory parameters;
- Vital signs;
- Physical examination;
- 12-lead ECG;
- ADA response.

Pharmacokinetics

- $AUC_{0-\tau}$: Area under the plasma concentration-time over the dosing interval;

- $AUC_{0\text{-}tlast}$: Area under the plasma concentration-time curve from time zero to the last quantifiable concentration;
- $AUC_{0\text{-}\infty}$: Area under the plasma concentration-time curve from time zero to infinity;
- C_{max}: Maximum observed plasma concentration;
- T_{max}: Time of the maximum observed plasma concentration;
- $t_{1/2}$: Apparent terminal elimination half-life;
- CL: Total plasma clearance;
- V_z: Apparent volume of distribution during the terminal elimination phase;
- V_{ss}: Apparent volume of distribution at steady state;
- RA_{AUC}: Accumulation ratio based on $AUC_{0\text{-}\tau}$;
- RA_{Cmax} Accumulation ratio based on C_{max};
- RL: Ratio of Linearity.

In addition, dose normalized values (norm) for $AUC_{0\text{-}\tau}$ and C_{max} will be determined by dividing the original toxicokinetic parameter by the dose level.

Efficacy

- Assessment of tumor response (RECIST) by MRI or CT;
- Tumor vascularity and perfusion (DCE-MRI);
- Biomarkers: PlGF (placental growth factor) and one additional biomarker of interest;
- ECOG.

Statistical methods:

All patients enrolled in the study and receiving at least one dose of study medication will be included in the evaluation of safety and efficacy. No formal hypothesis will be stated and statistically tested. All parameters will be descriptively analyzed using standard statistical methods. Tables and graphs, as well as patient listings will be presented by dose groups.

Dosing flow chart

The first cohort of three patients will be treated with 0.1 mg/kg Angiocal®. Doses will be increased according to the following dose escalation schedule in case no DLT occurs after the single dose application within the follow-up period (until Day 15):

Week	Dose level (mg/kg)						
	0.1	0.5	1.5	3.0	6.0	10.0	15.0
0	S						
1							
2		S					
3	R						
4	R		S				
5	R	R					
6	R	R		S			
7		R	R				
8		R	R		S		
9			R	R			
10			R	R		S	
11				R	R		
12				R	R		S
13					R	R	
14					R	R	
15						R	R
16						R	R
17							R
18							R

S = single dose, R = repeated dose.

Each patient will come to the study site according to the following schedule:

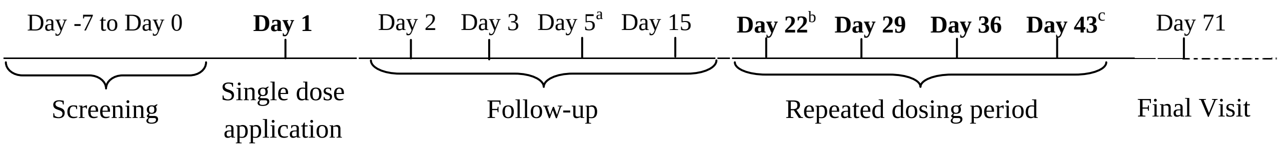

[a] Visit on Day 5 is optional and is at the discretion of the investigator.
[b] In the absence of any DLT and based on the patient’s and investigator's decision, patients will continue into the repeated dosing period.
[c] Patients who respond to Angiocal® treatment or have stable disease will, at the discretion of the investigator, be given the opportunity to get further treatment with Angiocal® in a biweekly schedule. The Final Visit at Day 71 will not be performed in these patients.

Schedule of assessments (single dose period)

	Screening		Single treatment		Follow-up	
	(Day -7 to Day 0)	(Day 1) Baseline	Day 2 (24 h ±2 h)	Day 3 (48 h ±2 h)	Day 5 (96 h ±2 h)	Day 15
	Visit 1	Visit 2	Visit 3	Visit 4	Visit 5 - optional	Visit 6
Informed consent	x[1]					
Inclusion and exclusion criteria	x					
Medical history	x					
Brain CT scan if metastases are expected	x[1]					
Demographic data	x					
Height	x					
Pregnancy test for women of childbearing potential	x					
Previous medication	x[2]					
Anti-Drug Antibody response	x					
DCE-MRI	x		x			
MRI/CT staging (RECIST)	x[1]					
Physical examination	x	x	x			
Weight	x	x				
Administration of study medication		x				
12-lead ECG	x		x			
Concomitant medication	x	x	x	x	x	x
ECOG performance status	x	x	x			
Vital signs	x	x[3]	x	x	x	x
Blood samples for biomarkers[4]		x	x	x	x	x
Hematology	x	x	x			x
Coagulation	x	x	x			
Clinical chemistry	x	x	x			
Urinalysis	x	x	x			
Blood samples for PK		x[5]	x	x	x	x
AEs/toxicity (NCI-CTCAE v3.0)		x	x	x	x	x

Please see next page for footnotes

Schedule of assessments (repeated dosing period)

	**Repeated dosing period**				**Final Visit**	**Biweekly dosing period**
	Day 22	Day 29	Day 36	Day 43	Day 71	starting on
	Visit 7	Visit 8	Visit 9	Visit 10	Visit 11	Day 57
Anti-drug antibody response				x	x	
DCE-MRI				x		
MRI/CT staging (RECIST)				x		x[7]
Physical examination	x			x	x	x
Weight	x	x	x	x	x	
Administration of study medication	x	x	x	x		x
12-lead ECG	x[6]	x[6]	x[6]	x[6]	x	
Concomitant medication	x	x	x	x	x	
ECOG performance status	x			x	x	x
Vital signs	x[3]	x[3]	x[3]	x[3]	x	x
Blood samples for biomarkers[4]				x		
Hematology	x	x	x	x	x	x
Coagulation	x	x	x	x	x	x
Clinical chemistry	x	x	x	x	x	x
Urinalysis	x	x	x	x	x	x
Blood samples for PK	x	x	x	x	x	
AEs/toxicity (NCI-CTCAE v3.0)	x	x	x	x	x	x

At Days 1, 22, 29, 36, and 43 all assessments will be performed pre-dose if not otherwise noted. AEs/toxicity will be recorded pre- and post-dose.

[1] To be performed within 28 days prior to Day 1.

[2] Taken during 30 days prior to study start.

[3] Pulse rate and blood pressure will be assessed pre-dose and 30 min, 1 h, 2 h, 4 h and 8 h post-dose.

[4] Biomarkers: PlGF and one additional biomarker of interest.

[5] Blood samples will be collected 5 min pre-dose and 15 min, 30 min, 1 h, 2 h, 4 h, 8 h, 24 h (Day 2), 48 h (Day 3) ±5 min for each time point] and Days 5 and 15 [±2 h of time study drug was administered at Day 1 for each time point] post-dose.

[6] To be performed 2 h post-dose.

[7] To be performed after every fourth dose of treatment. Treatment will stop in case of progressive disease.

AE = adverse event, CT = computed tomography, CTC = Common Toxicity Criteria, DCE-MRI = Dynamic Contrast-Enhanced Magnetic Resonance Imaging, ECG = electrocardiogram, ECOG = Eastern Cooperative Oncology Group, MRI = Magnetic Resonance Imaging, NCI = National Cancer Institute, PK = pharmacokinetics, PlGF = placental growth factor, RECIST = Response Evaluation Criteria In Solid Tumors.

2 Addresses and responsibilities

Sponsor signatory

Dr. Angelika C. Stern
Pieris AG
Lise-Meitner-Straße 30
85354 Freising
Germany

Coordinating investigator

PD Dr. med. Klaus Mross
Tumor Biology Center at the Albert-Ludwigs-University Freiburg
Department of Medical Oncology
Breisacher Str. 117
76106 Freiburg im Breisgau
Germany

Sponsor's Medical Expert

Dr. Angelika C. Stern
Pieris AG
Lise-Meitner-Straße 30
85354 Freising
Germany

Medical Monitor

Dr. med. Edgar J. Fenzl
FGK Clinical Research GmbH
Heimeranstrasse 35
80339 München
Germany

3 Table of contents

Page

1 SUMMARY AND FLOW CHART 2
2 ADDRESSES AND RESPONSIBILITIES 13
3 TABLE OF CONTENTS 14
4 ABBREVIATIONS 18
5 INTRODUCTION 20
5.1 Background information 20
5.2 Angiocal® (PRS-050-PEG40) 21
5.2.1 Structure and function 21
5.2.2 Non-clinical pharmacology 22
5.2.3 Pharmacokinetics and biodistribution in animal studies 23
5.2.4 Toxicology 23
6 INVESTIGATORS AND STUDY ADMINISTRATIVE STRUCTURE 24
7 STUDY OBJECTIVES 25
8 STUDY DESIGN AND DESIGN RATIONALE 25
8.1 Overall study design 25
8.2 Dose escalation 26
8.2.1 Dose levels 26
8.2.2 Dose escalation decision rules 26
8.3 Study design rationale 27
8.4 Risk and benefit assessment 27
9 PATIENT SELECTION 28
9.1 Sample size 28
9.2 Inclusion criteria 28
9.3 Exclusion criteria 29
10 TREATMENTS 31
10.1 Investigational medicinal products 31
10.1.1 Description, packaging and labeling 31

10.1.2 Storage and stability ... 31
10.1.3 Administration ... 31
10.1.4 Drug accountability and patient compliance ... 32
10.1.5 Procedure in the case of missed doses ... 32
10.2 Previous and concomitant medication ... 32
10.3 Method of assigning patient numbers ... 33
11 STUDY SCHEDULE ... 33
11.1 Enrollment ... 33
11.2 Study conduct ... 33
11.3 Screening ... 33
11.4 Single dose application - Baseline (Day 1) ... 34
11.5 Follow-up ... 35
11.5.1 Day 2 ... 35
11.5.2 Day 3, Day 5 (optional) and Day 15 ... 35
11.6 Repeated dosing period (Days 22, 29, 36, and 43) ... 36
11.7 Biweekly dosing period ... 37
11.8 Final Visit - Day 71 ... 37
11.9 Study termination ... 38
11.10 Time windows ... 38
12 SAFETY ASSESSMENTS ... 39
12.1 Adverse events ... 39
12.1.1 Definition of adverse events ... 39
12.1.2 Causality and severity assessment ... 41
12.1.3 Documentation of adverse events ... 42
12.1.4 Reporting of SAEs ... 42
12.1.5 Follow up of adverse events ... 43
12.1.6 Dose limiting toxicity ... 43
12.2 Laboratory investigations ... 44
12.3 Assessment of anti-drug antibodies ... 45
12.4 Electrocardiogram ... 45
12.5 Vital signs ... 46
12.6 Physical examination ... 46

13 PHARMACOKINETICS ... 46
13.1 Specimen collection and shipment ... 46
13.2 Pharmacokinetic assessments ... 47
14 EFFICACY ASSESSMENTS ... 47
14.1 Tumor measurements ... 47
14.2 Eastern Cooperative Oncology Group (ECOG) performance status ... 48
14.3 Assessment of biomarkers ... 49
15 BIOSTATISTICAL METHODS ... 49
15.1 Analysis sets and types of analyses ... 49
15.2 Analysis of study conduct and patient disposition ... 49
15.3 Analysis of Baseline characteristics ... 49
15.4 Safety Analyses ... 50
15.4.1 Adverse events ... 50
15.4.2 Dose limiting toxicity (DLT) ... 50
15.4.3 Clinical laboratory measures ... 50
15.4.4 Anti-drug antibodies ... 50
15.4.5 Electrocardiogram ... 50
15.4.6 Vital signs ... 50
15.4.7 Physical examination ... 51
15.5 Pharmacokinetic analysis ... 51
15.6 Efficacy Analyses ... 51
15.6.1 Biomarkers ... 51
15.6.2 Tumor measurements ... 51
15.6.3 ECOG performance status ... 51
15.7 Interim analysis ... 51
16 PATIENT WITHDRAWAL FROM STUDY PARTICIPATION ... 52
17 ETHICAL AND LEGAL REQUIREMENTS ... 53
17.1 General requirements ... 53
17.2 Independent ethics committee and competent authority approval ... 53
17.3 Patient information and consent procedure ... 53
17.4 Insurance coverage ... 54

18 CRITERIA FOR PREMATURE TERMINATION OF THE STUDY AND CRITERIA FOR CLOSING A STUDY CENTER.. 54

19 DATA COLLECTION 54

20 ARCHIVING OF DATA 55

21 MONITORING AND QUALITY ASSURANCE 55

22 STUDY REPORT AND PUBLICATIONS 56

23 STUDY PERIODS 57

24 REFERENCES.. 58

25 CHANGES TO PROTOCOL VERSION 1.0... 59

26 APPROVAL AND SIGNATURES.. 60

27 APPENDIX: PROTOCOL MR-IMAGING... 62

4 Abbreviations

ADR	Adverse drug reaction
AE	Adverse event
ALT	Alanine aminotransferase
aPTT	Activated partial thromboplastin time
AST	Aspartate aminotransferase
$AUC_{0-\tau}$	Area under the plasma concentration-time over the dosing interval;
$AUC_{0-tlast}$	Area under the plasma concentration-time curve from time zero to the last quantifiable concentration
$AUC_{0-\infty}$	Area under the plasma concentration-time curve from time zero to infinity
CL	Total plasma clearance
C_{max}	Maximum observed plasma concentration
CRO	Contract Research Organization
CT	Computed tomography
CTC	Common toxicity criteria
DCE-MRI	Dynamic contrast-enhanced magnetic resonance imaging
DRF	Dose range finding
EC	Ethics Committee
ECG	Electrocardiogram
ECOG	Eastern Cooperative Oncology Group
eCRF	Electronic Case Report Form
GCP	Good Clinical Practice
GLP	Good Laboratory Practice
GMP	Good Manufacturing Practices
HED	Human equivalent dose
HUVEC	Human Umbilical Vein Endothelial Cell
iAUC	Initial area under the contrast agent concentration curve
$iAUC_{60,90,120}$	iAUC of the first 60, 90, and 120 seconds after contrast agent arrival
ICH	International Conference on Harmonisation
INR	International Normalized Ratio
K^{trans}	Transfer constant
MRI	Magnetic resonance imaging
MTD	Maximum tolerated dose
NCI	National Cancer Institute
NYHA	New York Heart Association

PBS	Phosphate buffered saline
PK	Pharmacokinetics
PlGF	Placental growth factor
PT	Prothrombin time
q2d	Every second day
q3d	Every third day
R10	Baseline relaxation rate
RA_{AUC}	Accumulation ratio based on $AUC_{0-\tau}$
RA_{Cmax}	Accumulation ratio based on C_{max}
RBC	Red blood cell count
RECIST	Response Evaluation Criteria In Solid Tumors
RL	Ratio of Linearity
SAE	Serious adverse event
SAP	Statistical analysis plan
SUSAR	Suspected Unexpected Serious Adverse Reactions
TK	Toxicokinetics
$t_{1/2}$	Apparent terminal elimination half-life
t_{max}	Time of the maximum observed plasma concentration
ULN	Upper limit of normal
VEGF	Vascular Endothelial Growth Factor
VEGFR	Vascular Endothelial Growth Factor Receptor
V_{ss}	Apparent volume of distribution at steady state
V_z	Apparent volume of distribution during the terminal elimination phase
WBC	White blood cell count

5 Introduction

5.1 Background information

One characteristic in cancer is that angiogenesis, the process of forming new blood vessels, and the development of metastases are intrinsically connected. The growth of tumors and subsequent development of metastasis rely on the formation of new blood vessels.[1,2]

Angiogenesis is essential for primary and metastasized tumors since all cells must be within 100-200 µm of blood vessels in order to receive essential oxygen and nutrients.[3] Sprouting of pre-existing vessels requires major reorganization involving destabilization of the mature vessels, proliferation, migration of endothelial cells and maturation. The proangiogenic factor Vascular Endothelial Growth Factor (VEGF-A) has been demonstrated to be a major contributor to angiogenesis by increasing the number of capillaries in a given network. VEGF is a diffusible glycoprotein produced by normal and neoplastic cells and is upregulated in most human tumor types.[4] The VEGF family comprises seven members: VEGF-A, VEGF-B, VEGF-C, VEGF-D, VEGF-E, placental growth factor (PlGF) and VEGF-F.[5] Of these, VEGF-A, also called VEGF, is the dominant glycoprotein and the most extensively studied member of the VEGF family. To date at least 7 splice variants of VEGF, which vary in numbers of amino acids, have been characterized: $VEGF_{121}$, $VEGF_{145}$, $VEGF_{148}$, $VEGF_{165}$, $VEGF_{183}$, $VEGF_{189}$, and $VEGF_{206}$.[6]

VEGF binds to its receptors VEGFR-1 and VEGFR-2. The binding of VEGF to VEGFR-2 triggers dimerization and ligand-dependent phosphorylation of the receptor.[10] This activation of the receptor leads to mitogenic and pro-survival signals that are transferred downstream to Raf-Mek-Erk and the PI3K-Akt pathways.[7-9] Therefore, it is assumed that the VEGF/VEGFR-2 complex is the predominant pathway driving endothelial cell mitogenesis, migration, survival and vascular permeability.[11] A large and growing body of evidence shows that increased levels of VEGF expression are associated with a range of solid tumor types such as breast, lung, cervix, ovarian, pancreatic, gastrointestinal, kidney, bladder, renal cancer and glioblastoma. Increased VEGF serum levels in these cancer types have been correlated with poor survival.

The importance of the VEGF family and VEGFRs in tumor angiogenesis led to development of new therapeutic strategies targeting either VEGFs or VEGFRs. Molecular target-based approach aims to improve the efficacy and selectivity of cancer treatment by developing molecules that block specific pathogenic mechanisms which account for malignant transformation. Unlike targeted drugs, traditional cytotoxic anticancer agents are being discovered through random screening. Cytotoxic cancer agents are developed to target DNA, tubulin or molecules involved in cell division. As a result, the pharmacological effects of most of these cytotoxic drugs are nonselective and their cytotoxicity is irreversible. On the

other hand, binding of a targeted drug to its target has been shown to be likely cytostatic rather than cytotoxic.[12] In summary, molecularly targeted drugs interact with proteins that are specific in tumor progression or upregulated during malignant transformation. Compared to traditional cytotoxic anticancer agents the target-based therapy promises to be more selective and less toxic to normal tissue.

5.2 Angiocal® (PRS-050-PEG40)

5.2.1 Structure and function

PRS-050 belongs to a new class of therapeutic proteins, Anticalins®, which are engineered lipocalins, developed by Pieris AG (Pieris) and which are based on a proprietary lipocalin Anticalin® technology. Anticalins® are homologous to naturally occurring lipocalins and can be generated against a variety of targets using mutation and selection processes. The Anticalin® technology provides proteins with a compact protein structure, high intrinsic stability, and a small molecular size. The latter property may allow the anticalin to penetrate neovascularized tumor tissue more effectively. In addition, this technology offers a broad formulation flexibility.

Human tear lipocalin (Tlc, Lcn1) was used as a protein scaffold to engineer the PRS-050 'Anticalin' which specifically binds and antagonizes the function of VEGF-A. Starting from a naïve combinatorial library, PRS-050 was selected based on its ability to bind to all splice forms of VEGF-A with picomolar affinity. In-vitro studies showed that PRS-050 efficiently antagonizes the interaction of VEGF-A with its cellular transmembrane tyrosine kinase receptors. PRS-050 was coupled to polyethylene glycol (PEG) to extend the half life of the protein in plasma. The pegylated protein (PRS-050-PEG40) has a molecular weight of about 58 kDa.

The following variants of Angiocal® were used in preclinical studies:

PRS-050#002:	Original clone harboring an eptitope tag (StrepTag); periplasmatic expression.
PRS-050#006:	Original clone fused to an Albumin-binding domain to extend the plasma half-life; periplasmatic expression.
PRS-050#009-PEG40:	A free cysteine residue was introduced to facilitate site-directed pegylation; this version still features a StrepTag; periplasmatic expression.
PRS-050#011-PEG40: (Angiocal® PRS-050-PEG40)	Containing the free cysteine and codon usage optimized for bacterial expression; the StrepTag was removed; expressed in secreted form; final development candidate.

5.2.2 Non-clinical pharmacology

A series of pharmacology studies were performed to evaluate the specificity, mechanism of action and biological activity of PRS-050-PEG40 or its earlier variants.

The ability of PRS-050 to interfere with VEGF-induced mitogenic signaling and proliferation was investigated *in vitro* with PRS-050#002 using primary Human Umbilical Vein Endothelial cells (HUVECs). Stimulation of HUVECs with VEGF leads to dual phosphorylation and activation of ERK 1 and ERK 2 via VEGF binding to VEGFR-2. PRS-050#002 potently inhibited this VEGF-induced ERK phosphorylation similar to Avastin®. Furthermore, PRS-050#002 inhibited VEGF-induced HUVEC cell proliferation also with a similar potency than Avastin®. Inhibition of VEGF-induced HUVEC cell proliferation was also demonstrated for Angiocal®.

PRS-050#006, a PRS-050 variant which carries a short peptide mediating serum albumin binding to extend the plasma half-life (see above) demonstrated significant anti-tumor activity in a human rhabdomyosarcoma model in nude mice. PRS-050#006 also significantly inhibited VEGF-induced blood vessel formation in an *in-vivo* angiogenesis assay consisting of VEGF-induced blood vessel formation in matrigel-filled angioreactors in mice.

The pegylated variant was tested in an *in-vivo* vascular permeability assay in guinea pigs. PRS-050#009PEG40 significantly inhibited vascular permeability similar to Avastin®. It was also shown to inhibit tumor growth in a human colon cancer model in rats similar to the positive control Sorefanib, a registered multikinase inhibitor which inhibits tumor growth of a broad spectrum of human tumor xenografts.

To bridge the preclinical studies conducted with “pre-versions” with the final candidate PRS-050#011PEG40, PRS-050#011PEG40 was also tested in the human rhabdomyosarcoma xenograft model for its antitumor activity. In this study, a significantly slower tumor growth was observed with PRS-050#011PEG40 compared to vehicle and the increase of tumor volume under PRS-050#011PEG40 treatment was significantly slower compared with Avastin® treatment. The antitumor activity of PRS-050#011PEG40 was further characterized in a human colon xenograft model in nude rats. Tumors were treated with PRS-050#011PEG40 or Sorafenib as control and tumor growth was monitored. Tumor growth was significantly decreased with both compounds when compared to vehicle. In addition to tumor growth monitoring, dynamic contrast enhanced magnetic resonance imaging (DCE-MRI) was employed to gain an understanding of effects of PRS-050#011PEG40 on perfusion and permeability (K^{trans} parameter) of tumors at early time points before the inhibition of tumor growth becomes apparent. In the tumor rim, a drop in the relevant K^{trans} parameter was observed as early as 3 days after treatment start, making DCE-MRI an ideal modality to monitor immediate treatment effects in the clinical program.

5.2.3 Pharmacokinetics and biodistribution in animal studies

Pharmacokinetics

Kinetics of PRS-050-PEG40 in plasma were assessed in mice, rats and cynomolgus monkeys after single i.v. dosing and in rat and cynomolgus monkeys after single and repeated i.v. administration The plasma kinetics of PRS-050-PEG40 were essentially linear following i.v. administration in both rat and cynomolgus monkey. Elimination half-lives, clearance and volume of distribution were independent from the dose administered. Also, there seemed to be no sex-linked difference in the plasma kinetics in the rodent as well as non-rodent species. Upon repeated i.v. dosing accumulation of PRS-050-PEG40 in plasma was noted in rat and cynomolgus monkey.

Key pharmacokinetic parameters after single dosing are summarized in the table below:

Species (Study no.)	$t_{½}$ (h)	AUC_{inf} (h*µg/mL)	V_{ss} (mL/kg)	CL (mL/h/kg)
Mouse (PPS_008_07)	28.26	6188	64.81	1.616
Rat (PPS_003_08)	41.53	8703	57.66	1.149
Cynomolgus monkey (PPS_004_08)	58.51	15930	47.13	0.622

The cynomolgus monkey represents the species best suited to allow predictions to PRS-050-PEG40 pharmacokinetics (PK) behaviour. However, PK data from mouse and rat were also taken into account for allometric scaling, which revealed a terminal elimination half life of PRS-050-PEG40 of about 5 days for humans.

Distribution

In a bio-distribution study with radio-labeled PRS-050#11-PEG40 in the rat no undue tissue accumulation of PRS-050-PEG40 in normal and healthy rats was observed. Maximum concentration of PRS-050-PEG40 was found in heart, lungs, spleen, liver and kidneys at peak activity uptake. Renal clearance was the major route of elimination with fewer amounts excreted in feces.

5.2.4 Toxicology

Repeated dose, 4-week i.v. toxicity studies were conducted in the rat and cynomolgus monkey Dosing intervals in the rat (q2d = 2-day interval) and cynomolgus (q3d = 3-day interval) toxicity studies were selected based on the PK parameters.

Treatments were generally well tolerated and no major signs of toxicity were seen after

treatment with PRS-050-PEG40. PRS-050-PEG40 showed good local and systemic tolerability in both species and no mortality, no overt signs of toxicity, no late toxicity, and no effects on vital functions were noted in the dose range finding (DRF) and Good Laboratory Practice (GLP) toxicity studies. In addition, no evidence of immunotoxicity up to 200 mg/kg q2d in the rat DRF (125 mg/kg in the 4-week GLP study) and 150 mg/kg q3d in the cynomolgus monkey DRF (100 mg/kg in the 4-week GLP study) was noted. There were also no effects on either the cardiovascular (cynomolgus monkey) or the central nervous (rat) safety pharmacology parameters up to the highest doses tested of 100 mg/kg q3d or 125 mg/kg q2d, respectively. Some findings, such as alterations of incisors (rat-specific finding), low body weight gain and bone changes in rats or monkeys, were clearly attributable to the mode of action of PRS-050-PEG40 and are thus not considered adverse. The effect on the female reproductive organs (reduced organ weights of uterus and ovaries) in cynomolgus monkey was also expected due to the anti-angiogenic activity of PRS-050-PEG40. The No-Observed-Adverse Effect level (NOAEL) for the 4-week rat study, based on microscopic findings in the kidney, was considered to be 20 mg/kg/occasion. Based on renal tubular vacuolation, in the cynomolgus monkey study, the NOAEL was considered to be 45 mg/kg/dose.

6 Investigators and study administrative structure

The study is planned to be conducted at three centers in Germany coordinated by PD Dr. med. Klaus Mross (Coordinating Investigator).

The sponsor is responsible for supervision of the study. FGK Clinical Research GmbH, a Contract Research Organization (CRO), will provide medical monitoring, project management, site monitoring, handling of adverse events (AE) reporting, consulting services for medical and regulatory issues, data management, statistical analysis and report writing. Addresses and telephone numbers of main responsible persons are provided in Section 2.

Anti-drug antibodies (ADA) and PK samples will be analyzed by Covance Ltd., Harrogate, United Kingdom and PK data analysis will be done at Covance Clinical Research Unit, Leeds, United Kingdom. All other laboratory analyses will be performed by the local laboratories of the centers. DCE-MRI data will be analyzed by the Magnetic Resonance Development and Application Center, Freiburg, Germany.

7 Study objectives

Primary objective:

- To evaluate the safety and tolerability of Angiocal® when administered intravenously as a slow bolus injection to patients with solid tumors.

Secondary objectives:

- To characterize the pharmacodynamic response and evaluate the pharmacokinetic profile of Angiocal®;
- To evaluate the effectiveness of Angiocal® in terms of tumor response.

8 Study design and design rationale

8.1 Overall study design

This is a Phase I, open-label, dose escalation study with Angiocal® in patients with solid tumors (cancer patients excluding patients with lymphomas) using a group sequential adaptive treatment assignment. Patients will be allocated to different dose levels in small cohorts and will receive a total of 5 intravenous (i.v.) applications of Angiocal®: one single dose application on Day 1 and one application once weekly for 4 weeks during a repeated dosing period. The single dose application will be followed by a 3-week follow-up period prior to start of the repeated dosing period. The time between the single dose and the repeated dosing period may be adjusted should the plasma half-life of the drug differ from the predicted duration based on pre-clinical studies. Patients will be evaluated at Screening (Day -7 to 0) and Baseline (Day 1). Follow-up visits will be performed on Days 2, 3, 5 (optional), and 15 after the single dose application. In the absence of any dose limiting toxicity (DLT) and based on the patient’s and investigator's decision, patients will continue with the repeated dosing period, where they will be treated and evaluated once weekly for 4 weeks on Days 22, 29, 36, and 43. A Final Visit will be performed on Day 71. For patients who discontinue after the follow-up period and do not continue to the repeated dosing period, assessments scheduled for the Day 22 and Day 29 Visit should be performed whenever possible.

In case of stable disease or response to treatment at Day 43 the investigator may decide to continue with Angiocal® treatment if the patient agrees. Angiocal® will then be given biweekly (every 2 weeks) starting at Day 57. The Final Visit at Day 71 will not be performed in these patients. The investigator will decide to continue treatment during the follow-up visits every two weeks. After every fourth dose of Angiocal® a CT scan will be performed to determine the progress of the disease. In case of progressive disease no further treatment with

Angiocal® will be given.

8.2 Dose escalation

8.2.1 Dose levels

Patients will be allocated to a dose level of Angiocal® in small cohorts of three patients. The first cohort will be allocated to the 0.1 mg/kg dose level. Within a cohort, treatment will occur sequentially: only one patient will be treated first, the next two patients will be treated after 48 hours of follow-up (Day 3) of the previously treated patient. The dose will be increased to the next higher level when Day 15 data of all three patients of one cohort are available (see dosing flow chart in Section 1). Treatment of subsequent cohorts will depend on the occurrence of dose limiting toxicity (DLT) of the previous cohort. For the definition of DLT see Section 12.1.6. The maximum dose level studied will be 15 mg/kg; however, the maximum dose may be decreased based on the judgment of the investigator and sponsor. In case of any arising safety issues dose increments to the next higher dose level may be reduced at the discretion of the investigator and in accordance with the sponsor.

Close correspondence between the participating study centers will assure the well-being of all study participants and to assure an adequate information communication system.

8.2.2 Dose escalation decision rules

The number of patients treated per cohort will be adjusted according to the occurrence of a DLT:

- If **none** of the patients in a cohort experiences a DLT, then the next cohort will receive the next higher dose (if current dose ≤15 mg/kg).
- If **one** patient in a cohort experiences a DLT, then 3 more patients will be allocated to the same dose level:
  - If none of these patients experiences a DLT, then the next cohort will receive the next higher dose (if current dose ≤15 mg/kg);
  - If one or more of these three patients experiences a DLT, then dose escalation will stop. If the current dose is >0.1 mg/kg the prior lower dose will be the maximum tolerated dose (MTD) and three (3) additional patients will be treated with the highest dose below the MTD, if only three patients were treated previously at that dose.
- If **two** or more patients in a cohort experience a DLT dose escalation will stop. If the current dose is >0.1 mg/kg the previous lower dose will be the MTD and three (3) additional patients will be treated with the highest dose below the MTD, if only three patients were treated previously at that dose.

Patients who experience a DLT after the single dose application within the follow-up period will not continue into the repeated dosing period. Patients who experience a DLT event during the repeated dosing period will be withdrawn from the study.

8.3 Study design rationale

The chosen 3+3 design for the study is a standard design used in phase I dose escalating studies to determine how a drug is tolerated in people and it is often used in first-in-men studies.

The starting dose was based on the NOAEL in cynomolgus monkeys of 45 mg/kg as the most relevant species and rats of 20 mg/kg as the most sensitive species. It was defined to contain a safety factor of 10 below the NOAEL levels and to be close to but underneath the dose were minimal biological activity was observed. Taken into account a human equivalent dose (HED) factor of 3.1 for monkeys and 6.2 for rat and the safety factor of 10 the starting doses would be 1.5 mg/kg or 0.3 mg/kg based on the NOAEL. The starting dose was further reduced to 0.1 mg/kg to be below the minimal biological activity, which was seen at a HED of 0.2 mg/kg. The starting dose is thus 145-fold lower than the NOAEL in the most relevant species in terms of target identity and 32-fold lower than the NOAEL as observed in rat, the most sensitive species. It is reasonable to expect that this starting dose will be safe in humans.

8.4 Risk and benefit assessment

For patients suffering from solid cancer, surgery is the most important form of treatment. However, only small tumors and easily accessible tumors can be removed. Other conventional treatment options like chemotherapy and radiation therapy often produce side effects, leading to an impaired quality of life. Treatment alternatives are urgently needed for patients suffering from these deadly tumors associated with short life expectancy. Inhibition of tumor growth and prevention of metastasis formation are the most important aspects of cancer treatment.

Angiocal® specifically inhibits the binding of human VEGF to its receptor and thereby effectively blocks the signalling activity of VEGF. In appropriate preclinical models Angiocal® inhibited angiogenesis and vascular permeability and displayed a strong anti-tumor activity. The drug exhibits a comparable binding and functional activity profile in direct comparison to currently approved VEGF antagonists e.g. Avastin® (bevacizumab). The anticipated lowest human effective dose range of Angiocal® is between 0.2 and 0.8 mg/kg i.v. Thus the starting dose of 0.1 mg/kg is below but close to the dose at which minimal biological activity was observed in animal models.

In toxicity studies, Angiocal® was generally well tolerated and no major signs of toxicity were seen. Minor reversible changes in laboratory diagnostic parameters were noted in animals treated with PRS-050-PEG40. In addition, toxicologically relevant morphological

changes in the kidneys indicative of target organ toxicity were recorded in the rat and monkey at repeated i.v. doses of ≥50 mg/kg q2d or 100 mg/kg q3d, respectively. However, the red blood cell count, number of platelets, coagulation time as well as serum biochemistry (kidney function) parameters will be closely monitored in the study. In addition, robust safety margins are chosen. The starting dose in the study is 145-fold lower than the NOAEL in cynomolgus monkeys and 32-fold lower than the NOAEL in rats. The dose escalation scheme of the study further ensures that patients are not exposed to any undue risk.

Starting dose and dose escalation are thus selected in a way to provide a sufficient safety margin on the one hand side and a dose range which could be beneficial for the patient on the other side.

9 Patient selection

9.1 Sample size

No formal statistical hypotheses are being tested for this standard design dose escalating study. Decision rules for the number of patients to be treated per dose level are defined in Section 8.2.2. A maximum of 42 patients will be treated with Angiocal®, given that at each dose level one of three patients presents with a DLT and for the 2nd set of three patients at this dose level no DLT is observed. The maximum number of patients will be reached only in the following mathematically possible but rather unlikely escalating scenario:

Within each dose level: If within the first three patients exactly one patient experiences a DLT, three further patients will be treated at this dose level. If of these three patients no patient experiences a DLT, three further patients will be treated at the next higher dose level.

Executing this scheme to overall seven different doses, a maximum number of 7 (doses) x 6 (patients per dose) = 42 patients will be treated.

9.2 Inclusion criteria

All of the following criteria have to be met for including the patient in the study:

1. Males or females with pathologically confirmed diagnosis of advanced, recurrent or metastatic cancer, refractory to standard therapy. Patients with tumors for which no standard therapy exists will also be eligible;
2. Age ≥18 years;
3. Measurable or non-measurable disease according to Response Evaluation Criteria In Solid Tumors (RECIST), as determined by the investigator;

4. Eastern Cooperative Oncology Group (ECOG) performance status ≤2;
5. Estimated life expectancy of at least 3 months;
6. No current toxicities due to previous anticancer therapy (radiation therapy, chemotherapy or surgery);
7. Signed informed consent form and ability to understand the study procedures.

9.3 Exclusion criteria

The patient will not be eligible for the study if any of the following criteria applies:

1. Concomitant anticancer therapy, including radiation;
2. Current or previous (within 30 days of first study dosing) treatment with another investigational drug or participation in another clinical study;
3. Chronic daily treatment with aspirin (>325 mg/day) or clopidogrel (>75 mg/day);
4. Chronic daily treatment with corticosteroids (dose of ≥10 mg/day methylprednisolone or equivalent), with the exception of inhaled steroids;
5. Inadequate bone marrow function: absolute neutrophil count (ANC): <1.5 x 10^9/L, or platelet count <100 x 10^9/L or hemoglobin ≤10 g/dL;
6. Inadequate liver function, defined as:

- Serum (total) bilirubin >1.5 x the upper limit of normal (ULN), and/or
- Aspartate aminotransferase (AST) or alanine aminotransferase (ALT) >2.5 x ULN (>5 x ULN in patients with liver metastases);

7. Inadequate renal function, defined as:

- Serum creatinine >1.5 x ULN, and/or
- Creatinine clearance <50 mL/min (Calculated according to Cockroft and Gault), and/or
- Urine dipstick for proteinuria ≥2+ and >1 g of protein in the 24-hour urine (patients discovered to have ≥2+ proteinuria on dipstick urinalysis have to undergo a 24 hour urine collection and demonstrate ≤1 g of protein in the urine);

8. Patients not receiving anticoagulant medication who have an International Normalized Ratio (INR) >1.5 or an activated partial thromboplastin time (aPTT) >1.5 x ULN within 7 days prior to first study treatment. Note: Patients receiving full dose oral or parenteral anticoagulants may be included in the study as long as anticoagulant dosing has been stable for at least 2 weeks prior to study entry and the appropriate

coagulation monitoring tests are within local therapeutic limits;

9. Patients with lymphomas;
10. Evidence of spinal cord compression or brain metastases. A computed tomography (CT) or magnetic resonance imaging (MRI) of the brain must be performed if the presence of metastases at these sites is suspected (CT or MRI scans performed within 4 weeks prior to first study treatment may be sufficient);
11. Other malignancy diagnosed within the previous 5 years, except for adequately treated carcinoma *in situ* of the cervix or squamous carcinoma of the skin, or adequately controlled limited basal cell skin cancer;
12. Pregnant or lactating females. Serum pregnancy test must be performed within 7 days prior to study treatment start, or within 14 days with a confirmatory urine pregnancy test within 7 days prior to study treatment start;
13. All patients (including both female patients of childbearing potential and male patients with childbearing potential partners) who do not use a highly effective method of birth control (failure rate less than 1% per year when used consistently and correctly), e.g. implants, injectables, combined oral contraceptives in combination with a barrier method, some intrauterine contraceptive devices, sexual abstinence, or a vasectomized partner;
14. Major surgical procedure (including open biopsy) within 28 days prior to the first study treatment, or anticipation of the need for major surgery during the course of the study treatment;
15. Minor surgical procedures, within 24 hours prior to the first study treatment;
16. History or evidence of inherited bleeding diathesis or coagulopathy with the risk of bleeding;
17. Uncontrolled hypertension (systolic blood pressure >150 mmHg and/or diastolic blood pressure >100 mmHg) or clinically significant (i.e. active) cardiovascular disease: cerebrovascular accident/stroke within ≤6 months prior to the first study treatment, myocardial infarction within ≤6 months prior to the first study treatment, unstable angina, New York Heart Association (NYHA) Grade II or greater congestive heart failure, or serious cardiac arrhythmia requiring medication;
18. History of abdominal fistula, grade 4 bowel obstruction or gastrointestinal perforation, intra-abdominal abscess within 6 months of enrollment;
19. Lung carcinoma of squamous cell histology or any histology in close proximity to a major vessel;
20. Serious non-healing wound, peptic ulcer or bone fracture;

21. Known hypersensitivity to the study medication or any of its excipients;
22. Evidence of any other medical conditions (such as psychiatric illness, physical examination or laboratory findings) that may interfere with the planned treatment, affect patient compliance or place the patient at high risk of treatment-related complications;
23. Previous enrollment in this study;
24. Known hepatitis B or C or HIV infection;
25. Employees of the sponsor or patients who are employees or relatives of the investigator.

10 Treatments

10.1 Investigational medicinal products

10.1.1 Description, packaging and labeling

All study medication will be packed and labeled according to current Good Manufacturing Practices (GMP) guidelines, Good Clinical Practice (GCP) guidelines, and national legal requirements. Manufacturing and packaging will be done by Miltenyi Biotech GmbH, Teterow, Germany. Angiocal® will be supplied as a clear to opalescent colorless to slightly yellow sterile solution in phosphate buffered saline (PBS) with a pH of 7.4. The primary packaging material is composed of 10R sterile glass vials, 20 mm stoppers for injection and flip-off caps.

10.1.2 Storage and stability

The investigator is responsible for safe and proper handling and storage of the study medication at the investigational site. The study medication must be stored in a locked facility with access limited to the investigator and authorized personnel. The study medication has to be stored at -20°C (±5 °C) and needs to be thawed directly prior to its use. Once thawed it has to be transferred from the vials in 1 mL, 10 mL or 50 mL syringes depending on the drug volume to be applied during dose escalation. Detailed handling instructions will be provided. Within a period of 4 hours Angiocal® has to be administered to the patient. The investigator must ensure that the study medication is administered only to the patients enrolled in this study.

10.1.3 Administration

Angiocal® is to be administered intravenously by a slow bolus injection over 3 to 5 minutes. The volume to be administered to a patient is calculated according to handling instructions. Patients will receive 5 i.v. injections, one single treatment and 4 treatments during the repeated dosing period. During the repeated dosing period study medication will be administered once weekly for 4 weeks on Days 22, 29, 36, and 43.

10.1.4 Drug accountability and patient compliance

The study medication must not be used outside the context of this study protocol. The investigator or authorized staff is obliged to document the receipt, dispensation, and return of the study medication received during this study. Records on receipt, use, return, loss, or other disposition of study medication must be maintained. Records on study medication delivery to the site, the inventory at the site, the use by each patient, and the return to the sponsor must be maintained by the investigator and/or a pharmacist or another appropriately trained individual at the investigational site. The investigators must maintain records documenting that the patients were provided with the doses specified in the protocol. Furthermore, they should reconcile all study medication received from the sponsor. It is the responsibility of the investigator or the pharmacist to give reasons for any discrepancies in study medication accountability. Forms will be provided to improve drug accountability.

Following authorization by study management, drugs may be destroyed at the investigational site or by a licensed German facility following final accountability and in accordance with applicable regulations. Documentation of destruction is required. Alternatively, all unused products will be collected by the monitor or designee and returned to the sponsor or CRO for destruction.

10.1.5 Procedure in the case of missed doses

In the event that a dose cannot be administered according to the pre-specified schedule of repeated treatment counted from the day of administration of the first repeated dose, the respective dose should be omitted without substitution. Every effort should be made to comply with the dosage schedule.

10.2 Previous and concomitant medication

All medication taken during 30 days prior to study start as well as all concurrent medication taken by the patient during the course of the study will be documented in the corresponding section of the electronic case report form (eCRF) along with the indication and duration of treatment. Medication use will be reviewed by the investigator and checked against the exclusion criteria.

The use of any of the following medications is prohibited during the course of the study:

- Anticancer therapy;
- Aspirin;
- Clopidogrel;
- Corticosteroids.

10.3 Method of assigning patient numbers

Each patient who meets all eligibility criteria and is accepted for the study will be assigned a unique six-digit study number (e.g. 01-02-03) which will be composed of the study center (digits 1 and 2) the cohort (digits 3 and 4) and the individual patient number within the cohort (digits 5 and 6).

11 Study schedule

11.1 Enrollment

Patient enrollment will be done sequentially. Each dose level cohort will consist of three patients. Only one patient will be treated first, the next two patients will be treated after 48 h of follow-up of the previously treated patient.

11.2 Study conduct

An overview on study visits and study procedures is provided in the schedule of assessments (see Section 1).

11.3 Screening

Study participants will be recruited from the group of patients attending the investigators due to the study-specific indication. These can be patients attending the investigators independently or by referral from their general practitioners. The patients will be asked by the investigators for a general interest to participate in a clinical study. Patients for whom written informed consent (Section 17.3) has been obtained (within 28 days prior to Day 1) will undergo the following assessments:

- Review of inclusion and exclusion criteria;
- Recording of medical history;
- Brain CT scan if metastasis are expected (to be performed within 28 days prior to Day 1);

- Demographic data;
- Weight and height;
- Pregnancy test for women of childbearing potential;
- Previous (within 30 days prior to study start) and concomitant medication;
- Blood sampling for ADA response assessment in serum;
- MRI/CT staging according to RECIST (to be performed within 28 days prior to Day 1)[1];
- DCE-MRI (Note: not applicable if no target lesion was identified during MRI/CT);
- Physical examination;
- 12-lead electrocardiogram (ECG);
- ECOG performance status;
- Vital signs (heart rate, blood pressure and body temperature);
- Laboratory analysis including hematology, clinical chemistry, coagulation parameters, and urinalysis (see Section 14.2).

11.4 Single dose application - Baseline (Day 1)

The following assessments will be performed **before** the first i.v. injection of Angiocal®:

- Physical examination;
- Weight;
- Concomitant medication;
- ECOG performance status;
- Vital signs (heart rate, blood pressure and body temperature);
- Laboratory analysis including hematology, clinical chemistry, coagulation parameters, and urinalysis (see Section 14.2);
- Blood sampling for PK 5 min pre-dose;
- Blood samples for determination of PlGF in plasma and one additional biomarker of interest in serum;
- AEs and toxicity (National Cancer Institute Common Toxicity Criteria for AEs [NCI-

[1] Note: Failure to identify any target lesion does not lead to an exclusion of the patient from the study.

CTCAE] version 3.0).

The following assessments will take place **after** the first i.v. injection of Angiocal®:

- Blood sampling for PK: 15 min, 30 min, 1 h, 2 h, 4 h, and 8 h (±5 min for each time point) post-dose;
- Vital signs: pulse rate and blood pressure 30 min, 1 h, 2 h, 4 h and 8 h post-dose (If the patient agrees, the investigator may decide to perform a 24-hour blood pressure measurement. This may be reasonable in patients assigned to doses of Angiocal® of 6 mg/kg or higher);
- AEs and toxicity (NCI-CTCAE version 3.0).

11.5 Follow-up

11.5.1 Day 2

The following assessments will take place on Day 2:

- DCE-MRI (Note: not applicable if no target lesion was identified during MRI/CT at Screening);
- Physical examination;
- 12-lead ECG;
- Concomitant medication;
- ECOG performance status;
- Vital signs;
- Laboratory analysis including hematology, clinical chemistry, coagulation parameters, and urinalysis (see Section 14.2);
- Blood sampling for PK-sampling (24 h post-dose ±5 min);
- Blood samples for determination of PlGF in plasma and one additional biomarker of interest in serum;
- AEs and toxicity (NCI-CTCAE version 3.0).

11.5.2 Day 3, Day 5 (optional) and Day 15

The following assessments will take place:

- Concomitant medication;

- Vital signs (If the patient agrees, the investigator may decide to perform a 24-hour blood pressure measurement. This may be reasonable in patients assigned to doses of Angiocal® of 6 mg/kg or higher);
- AEs and toxicity (CTC AE version 3.0);
- Blood samples for determination of PlGF in plasma and one additional biomarker of interest in serum;
- Blood sampling for PK (Day 3: 48 h ±5 min post-dose; Day 5 and Day 15: ±2 h of the time of study drug administration on Day 1);
- Hematology (only on Day 15).

11.6 Repeated dosing period (Days 22, 29, 36, and 43)

The following assessments will take place **before** the first i.v. injection of Angiocal® during the repeated dosing period:

- Weight;
- Concomitant medication;
- Vital signs;
- Laboratory analysis including hematology, clinical chemistry, coagulation parameters, and urinalysis (see Section 14.2);
- Blood sampling for PK;
- AEs and toxicity (NCI-CTCAE version 3.0);
- Physical examination (only on Day 22 and Day 43);
- ECOG performance status (only on Day 22 and Day 43);
- DCE-MRI (only on Day 43; Note: not applicable if no target lesion was identified during MRI/CT at Screening);
- MRI/CT staging according to RECIST (only on Day 43; Note: not applicable if no target lesion was identified during MRI/CT at Screening).
- Blood sampling for ADA response assessment in serum (only on Day 43);
- Blood samples for determination of PlGF and one additional biomarker of interest in serum (only on Day 43).

Note: An emergency kit for the treatment of a potential anaphylactic shock should be readily available. The following assessments will take place **after** each i.v. injection of Angiocal®

during the repeated dosing period:

- 12-lead ECG: to be performed 2 h post-dose;
- Vital signs: pulse rate and blood pressure 30 min, 1 h, 2 h, 4 h and 8 h post-dose (If the patient agrees, the investigator may decide to perform a 24-hour blood pressure measurement. This may be reasonable in patients assigned to doses of Angiocal® of 6 mg/kg or higher);
- AEs and toxicity (NCI-CTCAE version 3.0).

11.7 Biweekly dosing period

In the absence of any DLT and in case of stable disease or response to treatment at Day 43 the investigator may decide to continue with Angiocal® treatment if the patient agrees. Angiocal® will be given biweekly (every 2 weeks) starting at Day 57. The investigator will decide to continue treatment during the follow-up visits every two weeks. The following assessments will take place:

- Physical examination;
- ECOG performance status;
- Vital signs;
- Laboratory analysis including hematology, clinical chemistry, coagulation parameters, and urinalysis (see Section 14.2);
- AEs and toxicity (NCI-CTCAE version 3.0);
- CT staging according to RECIST (only after every fourth dose; in case of progressive disease no further treatment with Angiocal® will be given).

11.8 Final Visit - Day 71

A Final Visit will be performed at Day 71 for patients who do not continue to the biweekly dosing period. The following assessments will take place on Day 71:

- Blood sampling for ADA response assessment in serum;
- Physical examination;
- Weight;
- 12-lead ECG;
- Concomitant medication;
- ECOG performance status;

- Vital signs (If the patient agrees, the investigator may decide to perform a 24-hour blood pressure measurement. This may be reasonable in patients assigned to doses of Angiocal® of 6 mg/kg or higher).
- Laboratory analysis including hematology, clinical chemistry, coagulation parameters, and urinalysis (see Section 14.2);
- PK-sampling;
- AEs and toxicity (NCI-CTCAE version 3.0).

11.9 Study termination

At study termination, the investigator will discuss with the patients how to further proceed with their treatment. Patients will be treated adequately by the investigator according to standard treatment guidelines.

For patients who prematurely discontinue or for patients who discontinue after the follow-up period and do not continue to the repeated dosing period, assessments scheduled for the Day 22 and Day 29 Visit should be performed whenever possible. In case of premature discontinuation after the Day 29 Visit the assessments planned for the Final Visit are to be performed whenever possible.

In the absence of any DLT and in case of stable disease or response to treatment at Day 43 the investigator may decide to continue with Angiocal® treatment if the patient agrees. Angiocal® will be given biweekly starting at Day 57. The Final Visit at Day 71 will not be performed in these patients. The investigator will decide to continue treatment during the follow-up visits every two weeks. After every fourth dose of Angiocal® a CT scan will be performed to determine the progress of the disease. In case of progressive disease no further treatment with Angiocal® will be given.

11.10 Time windows

The investigator will be asked to adhere to the predefined time window for each visit. Patients will be examined and evaluated according to the following schedule of visits:

- Visit 1: Screening (Day -7 to Day -0);
- Visit 2: Baseline (Day 1);
- Visit 3: Day 2 (±2 hours of the time study drug was administered at Day 1);
- Visit 4: Day 3 (±2 hours of the time study drug was administered at Day 1);
- Visit 5: Day 5 (±2 hours of the time study drug was administered at Day 1);

- Visit 6: Day 15;
- Visit 7: Day 22;
- Visit 8: Day 29;
- Visit 9: Day 36;
- Visit 10: Day 43;
- Visit 11: Day 71.

12 Safety assessments

12.1 Adverse events

12.1.1 Definition of adverse events

Adverse Events (AE)

An AE is defined as any untoward medical occurrence in a patient or clinical trial patient administered a trial product and which does not necessarily have a causal relationship with this treatment.

An AE can therefore be any unfavorable and unintended sign (including an abnormal laboratory finding), symptom, or disease temporarily associated with the use of a pharmaceutical product, whether or not causally related to the investigational medicinal product.

Whenever possible, an AE should be evaluated and reported as a diagnosis rather than individual symptoms and signs. If a definitive diagnosis is not possible, individual signs and symptoms should be reported.

The following should not be recorded as AEs, if recorded prior to Screening:

- pre-planned procedures unless the condition for which the procedure was planned has unexpectedly worsened from the first study-related activity after the patient has signed the informed consent;
- pre-existing conditions should not be reported as an AE unless there is a substantial increase in severity or frequency of this event (starting with the first application of the study product), or if this event cannot be attributed to natural causes. Any change of pre-existing conditions since Screening will be documented.

Adverse Drug Reactions (ADRs)

Adverse drug reactions (ADRs) of an investigational medicinal product are all untoward and unintended responses to an investigational medicinal product related to any dose administered.

All adverse events judged by either the reporting investigator or the sponsor as having a reasonable causal relationship to the investigational medicinal product qualify as ADRs. The expression 'reasonable causal relationship' means to convey in general that there is evidence or argument to suggest a causal relationship.

Events classified as possibly, probably or definitely related to the study medication will be regarded as having a reasonable causal relationship to the study medication.

Serious Adverse Event (SAE)

An AE is defined as serious if it results in any of the following outcomes:

- Results in death;
- Is life-threatening*;
- Requires hospitalization# or prolongation of existing in-patient hospitalization;
- Results in persistent or significant disability or incapacity;
- Involves congenital anomaly or birth defect;
- Is medically significant (an important medical event requiring medical or surgical intervention to prevent one of the outcomes listed above).

*A life-threatening AE is any AE that places the patient or subject, in the view of the investigator, at immediate risk of death from the reaction as it occurred (i.e. it does not include a reaction that, had it occurred in a more severe form, might have caused death).

#Hospitalization solely for the purpose of diagnostic tests, even if related to an adverse event, elective hospitalization for an intervention which was already planned before the inclusion of the patient in the study, and admission to a day-care facility may not themselves constitute sufficient grounds to be considered as a serious adverse event.

Medical and scientific judgment should be exercised in deciding whether an AE is serious in other situations, such as important medical events that may not be immediately life-threatening or result in death or hospitalization but may jeopardize the patient or may require intervention to prevent one of the other outcomes listed in the definition above. These should also be considered to be SAEs.

Suspected Unexpected Serious Adverse Reactions (SUSARs)

Suspected Unexpected Serious Adverse Reactions (SUSARs) are all suspected adverse reactions related to a tested investigational medicinal product that are both unexpected and serious. An unexpected adverse reaction is one that is not reported in the Investigators Brochure.

12.1.2 Causality and severity assessment

The investigator should use the following definitions when determining the severity of the AE:

- Grade 1: mild AE;
- Grade 2: moderate AE;
- Grade 3: severe AE;
- Grade 4: life-threatening or disabling AE;
- Grade 5: death related to AE.

For each AE an assessment must be made of the probability that it was related to the study treatment. The relationship of the AE to the study medication should be described using a scale consisting of the following categories of relationship:

- Not related: there is evidence that the event definitely has another cause than the study medication and does not meet any other criteria listed.
- Possibly related: a causal relationship is conceivable and cannot be dismissed;
- Probably related: there are good reasons and sufficient documentation to assume a causal relationship;
- Definitely related: strong evidence exists that the study medication caused the AE. There is a temporal relationship between the event onset and administration of the study medication. There is strong mechanistic evidence that the event was caused by the study medication. The patient’s clinical state and concomitant therapies have been ruled out as a cause.

12.1.3 Documentation of adverse events

All AEs will be assessed using the National (US) Cancer Institute-Common Toxicity Criteria for Adverse Events (NCI-CTCAE, version 3.0); where applicable, pre-study conditions will be graded using the same scale. For each episode, the highest severity grade attained should be recorded. AEs not included in the NCI-CTCAE are to be recorded, and intensity of AEs will be graded by the investigator using the criteria described in Section 12.1.2.

Information about all AEs, whether volunteered by the patient, discovered by the investigator questioning or detected through physical examination, laboratory test or other means, will be collected and recorded on the according pages of the CRF and in the patient's medical record.

The onset and end date, time, seriousness, severity and relationship to study medication will be recorded for each AE as well as any action (e.g. hospitalization, discontinuation of therapy) or outcome (e.g., recovered, recovered with sequelae). If known, the diagnosis should be recorded in preference to the listing of individual signs and symptoms. Information about SAEs will be reported by the investigator to the Medical Monitor within 24 hours of obtaining knowledge of the event.

If the AE meets the definition of an SAE, or if the investigator becomes aware of an unexpected AE that places the patient at risk or a pregnancy at any time after the study drug administration up to the end of the study follow-up period, the event must be documented and reported as described in Section 12.1.4. All AEs including SAEs experienced by a patient after the patient has signed the informed consent form until the end of the follow-up period, as specified in the protocol, must be reported.

12.1.4 Reporting of SAEs

Information about an SAE will be collected and recorded on the SAE Report Form. The investigator must assess the relationship to the study drug, complete the SAE Report Form in English, and send the completed, signed form by fax IMMEDIATELY within 24 hours to the Medical Monitor:

Dr. med. Edgar J. Fenzl
FGK Clinical Research GmbH
Heimeranstr. 35, 80339 München, Germany
Fax: +49-89-893119-20
Phone: +49-89-893119-22
Mobile: +49-172-673 8556
E-mail: edgar.fenzl@fgk-cro.de

The investigator will be requested to supply as much detailed information as possible regarding the SAE that is available at the time of the initial contact. The investigator should also complete missing or requested information and submit follow-up reports to the Medical

Monitor until the SAE has resolved or, in the case of permanent impairment, until the SAE has stabilized.

The Medical Monitor will inform the sponsor about all SUSARs within 24 hours. SAE reports which are not subject to expedited reporting will be sent to the sponsor by the Medical Monitor within 3 calendar days after receipt of the respective report from the investigator.

Reports should be directed to:

Dr. Angelika C. Stern
Pieris AG
Lise-Meitner-Straße 30
85354 Freising, Germany
Fax: +49-8161-1411-444
Phone: +49-8161-1411-400
E-mail: stern@pieris-ag.com

The Medical Monitor will ensure reporting of all SUSARs to competent authorities (CAs), ethics committees (ECs), and investigators according to the legal requirements. SUSARs that are fatal or life-threatening will be reported to the leading EC, CA, and investigators within 7 calendar days after first knowledge of the minimum criteria for expedited reporting. The initial notification will be followed by a complete report as possible within 8 additional calendar days. All other SUSARs that are not fatal or life-threatening will be reported to the leading EC, CAs, and investigators as soon as possible but no later than 15 calendar days after first knowledge of the information. All these reports will be provided to the sponsor in parallel.

12.1.5 Follow up of adverse events

Serious AEs or AEs leading to a patient's premature termination of the study will be followed up until the event has been resolved or stabilized, or the event has been diagnosed as chronic.

12.1.6 Dose limiting toxicity

DLT is defined as any of the following clinical toxicities, referencing National Cancer Institute Common Toxicity Criteria for adverse events (NCI-CTCAE v 3.0):

Non-hematological toxicity

- Any grade 3 or 4 toxicity, excluding nausea and vomiting, which responds to antiemetic treatment and alopecia;
- Prolonged (>2 weeks) grade 2 toxicities including serum creatinine 1.5 - 3 x ULN or calculated creatinine clearance <60 mL/min, increase in ALT, AST, alkaline phosphatase >2.5 - 5.0 x ULN, total bilirubin >1.5 - 3.0 ULN, intention tremor, slurred

speech, nystagamus, or dysmetria. Exception: patients with liver metastases at screening must have a prolonged (>2 weeks) grade 3 toxicity with an increase in ALT and AST >5.0 - 20.0 x ULN.

Hematological toxicity

- Grade 4 platelets and red blood cell toxicities,
- Grade 4 granulocyte toxicitiy lasting ≥7 days;
- Febrile neutropenia defined as an absolute neutrophils count <500/mm^3 together with fever either as 2 events of increased oral temperature (>38°C) with one hour interval or a single event of oral temperature (>38.5°C), provided that this single episode is not clearly related to other events.

Those events will be indicated as DLT if they occur after the single dose application within the follow-up period. Patients who experience one of those events during the repeated dosing period will be withdrawn from the study (see also Section 16).

12.2 Laboratory investigations

Laboratory assessments (hematology, biochemistry, coagulation parameters, urinalysis, and pregnancy test) will be performed at the local laboratory according to local practice. Laboratory reference ranges and certificates reflecting the validation of the tested parameters will be provided by the sites.

Blood samples should be taken using standard venous puncture techniques.

The clinical laboratory reports provided by the local laboratories will be reviewed by the investigator for clinical relevance. Laboratory values outside the normal range will be classified according to the following 3 criteria:

- Abnormal, clinically not significant;
- Abnormal, clinically significant;

  Abnormal clinically significant values must be recorded in the CRF as an AE unless the abnormal laboratory values are associated with a pathologic pre-existing condition with the abnormal values not representing a substantial change in magnitude compared to the respective screening value, or the abnormal laboratory value is associated with an AE already reported;
- Laboratory error.

Blood and urine samples for the determination of hematology, biochemistry, coagulation parameters, and urinalysis will be taken at Screening, Baseline, Day 2, Day 15 (only hematology), Day 22, Day 29, Day 36, Day 43 and the Final Visit. If necessary, blood and

urine samples for safety laboratory evaluation may be collected at any unscheduled visit.

The laboratory parameters to be assessed are as follows:

Hematology:	Total red blood cell count (RBC), hemoglobin, hematocrit, total white blood cell count (WBC), differential WBC, MCV (mean corpuscular volume), MCH (mean corpuscular hemoglobin), MCHC (mean corpuscular hemoglobin concentration),and platelet count;
Biochemistry:	Sodium, potassium, urea, creatinine, total bilirubin, gamma-glutamyl transferase (γ-GT), aspartate aminotransferase (AST), alanine aminotransferase (ALT), alkaline phosphatase, glucose, total protein, albumin, chloride, inorganic phosphorus, calcium, creatinine kinase, lactate dehydrogenase (LDH), C-reactive protein (CRP), cholesterol, triglycerides, uric acid, thyroid stimulating hormone (TSH);
Coagulation tests:	aPTT, prothrombin time (PT), fibrinogen;
Urinalysis:	pH, urobilinogen, erythrocytes, leucocytes, protein, ketones, bilirubin, nitrite, glucose.

For female patients of childbearing potential, a pregnancy test will be performed at Screening.

12.3 Assessment of anti-drug antibodies

Anti-drug antibodies will be analyzed by Covance Ltd., Harrogate, United Kingdom. Blood samples will be taken at Screening, on Day 43 and at the Final Visit on Day 71. Samples will be collected and stored at the site to be shipped to Covance in one shipment. Detailed written instructions regarding collection and storage of blood samples will be given to the investigator.

12.4 Electrocardiogram

A standard 12-lead ECG will be performed for each patient at Screening, Day 2, during the repeated dosing period 2 hours post-dose on Days 22, 29, 36, 43, and at the Final Visit. Results from 12-lead ECG will be recorded in the CRF using the assessment criteria ‘normal’, ‘abnormal, clinically not significant’ and ‘abnormal, clinically significant’.

12.5 Vital signs

Vital signs (heart rate, blood pressure and body temperature) will be measured at each visit. Systolic and diastolic blood pressure as well as pulse rate will be measured in sitting position after at least 5 minutes at rest. At Baseline, Days 22, 29, 36, and 43 pulse rate and blood pressure will be assessed pre-dose and 30 min, 1 h, 2 h, 4 h, and 8 h post-dose. If the patient agrees, the investigator may decide to perform a 24-hour blood pressure measurement. This may be reasonable in patients assigned to doses of Angiocal® of 6 mg/kg or higher.

12.6 Physical examination

A physical examination will be performed at Screening, Baseline, Days 2, 22, 43, and at the Final Visit and includes, as appropriate, clinical symptoms, bone pain, neurological symptoms, palpable tumors/regional lymph nodes, skin, and local injection reaction.

13 Pharmacokinetics

13.1 Specimen collection and shipment

Pharmacokinetic samples will be taken at the following time points during the study:

- Screening;
- Single treatment:
  - Pre-dose: 5 min;
  - Post-dose: 15 min, 30 min, 1 h, 2 h, 4 h, 8 h (±5 min for each time point);
- Follow-up period: Day 2 (48 h ±5 min), Day 3 (72 h ±5 min), Day 5 (96 h ±5 min, optional) and Day 15 (±2 h)
- Repeated dosing period: Days 22, 29, 36 and 43;
- Final Visit (Day 71).

Pharmacokinetic samples will be shipped to and analyzed by Covance Ltd., Harrogate, United Kingdom. Detailed written instructions regarding collection and storage of blood samples will be given to the investigator. PK data analysis will be done at Covance Clinical Research Unit, Leeds, United Kingdom.

13.2 Pharmacokinetic assessments

The following parameters will be assessed:

- $AUC_{0-\tau}$: Area under the plasma concentration-time over the dosing interval;
- $AUC_{0-tlast}$: Area under the plasma concentration-time curve from time zero to the last quantifiable concentration;
- $AUC_{0-\infty}$: Area under the plasma concentration-time curve from time zero to infinity;
- C_{max}: Maximum observed plasma concentration;
- T_{max}: Time of the maximum observed plasma concentration;
- $t_{½}$: Apparent terminal elimination half-life;
- CL: Total plasma clearance;
- V_z: Apparent volume of distribution during the terminal elimination phase;
- V_{ss}: Apparent volume of distribution at steady state;
- RA_{AUC}: Accumulation ratio based on $AUC_{0-\tau}$;
- RA_{Cmax} Accumulation ratio based on C_{max};
- RL: Ratio of Linearity.

In addition, dose normalized values (norm) for $AUC_{0-\tau}$ and C_{max} will be determined by dividing the original toxicokinetic parameter by the dose level.

14 Efficacy assessments

14.1 Tumor measurements

Tumor imaging will be performed using MRI or CT scans. A brain CT scan will only be required at Baseline in case of suspicion or evidence of brain metastases. The investigator should select one method of imaging at the beginning of the study and use that method throughout the study.

The investigator should be aware of the following contraindications for use related to the MR system:

MR contraindications (absolute)

- Active devices like cardiac pace maker, insulin pumps, nerve stimulators, cochlear implants and others as long as they are not explicitly MR compatible.

MR contraindications (relative, to be checked with the radiologists)

- Artificial heart valves, stent, penile implants, shrapnel/splinter, or aneurysm or other ferromagnetic clips or pins in the body;
- Non-removable piercings;
- Large tattoos made of metalliferous colors;
- Distinct claustrophobia;
- Osteosynthesis material.

Disease response will be assessed according to RECIST (Version 1.1) from within 28 days prior to Day 1 to Day 43.

Tumor vascularity and perfusion will be assessed by DCE-MRI as a possible biomarker at Screening, Day 2, and Day 43. Note that a failure to identify any target lesion does not lead to an exclusion of the patient from the study. The following parameters will be measured: transfer constant (K^{trans}), volume of extracellular extravascular space (V_e), baseline relaxation rate (R10) and the initial area under the contrast agent concentration curve (iAUC) of the first 60, 90, and 120 seconds after contrast agent arrival ($iAUC_{60}$, $iAUC_{90}$, and $iAUC_{120}$).

Details for the procedure and analysis are given in a separate protocol for MR imaging (please refer to the Appendix in Section 27). All data will be sent to and analyzed by the Magnetic Resonance Development and Application Center, Freiburg, Germany.

14.2 Eastern Cooperative Oncology Group (ECOG) performance status

ECOG performance status will be assessed at Screening, Baseline, and Days 2, 22, 43 and 71. The following scales and criteria are used to evaluate the impact of the disease on the patients' daily living abilities and to assess the patients' disease status:

0: Fully active, able to carry on all pre-disease performance without restriction

1: Restricted in physically strenuous activity but ambulatory and able to carry out work of a light or sedentary nature, e.g. light house work, office work

2: Ambulatory and capable of all self care but unable to carry out any work activities. Up and about more than 50% of waking hours

3: Capable of only limited self care, confined to bed or chair more than 50% of waking hours

4: Completely disabled. Cannot carry on any self care. Totally confined to bed or chair

5: Dead

14.3 Assessment of biomarkers

Blood samples for the analysis of PlGF in plasma and one additional biomarker of interest in serum will be taken at Day 1 (pre-dose) and Days 2, 3, 5 (optional), 15 and 43. Detailed written instructions regarding collection and storage of samples will be given to the investigator. Biomarker levels will be analyzed using a commercially available immunoassay.

15 Biostatistical methods

15.1 Analysis sets and types of analyses

All patients who are enrolled in the study and who receive at least one dose of study medication will be assessed to the safety analysis set and will be included in the evaluation of safety and efficacy.

No formal hypothesis will be stated and statistically tested. All parameters will be descriptively analyzed using standard statistical methods.

Tables and graphs, as well as patient listings will be presented by dose groups. Dose groups might be combined for tables and graphs in case of small numbers per dose groups. This will be further specified within the statistical analysis plan (SAP) or might be done on an ad hoc basis during analysis.

Further details on statistical analyses will be described within the SAP.

15.2 Analysis of study conduct and patient disposition

The number of patients enrolled and receiving at least one dose of study medication will be presented in detail to clearly describe the dose escalation pattern used in this study. The proportion of patients who prematurely discontinued the study will be summarized together with the reason of discontinuation.

15.3 Analysis of Baseline characteristics

Demographic variables such as sex, age, height, weight, ethnicity, and vital signs will be summarized, including frequency counts and percentages. No formal statistical comparisons of demographic and Baseline parameters will be made.

The distribution of males and females is according to the natural distribution within the study population for this study. No corrective measures will be taken. Pharmacokinetics in rats and cynomolgus monkeys showed no appreciable sex-linked differences and therefore no differences are expected in humans.

15.4 Safety Analyses

15.4.1 Adverse events

Adverse events will be summarized and provided as data listings. Verbatim terms will be mapped to preferred terms and organ systems using the current Medical Dictionary for Regulatory Activities (MedDRA) version. For each preferred term, frequency counts and percentages will be calculated. The nature, severity, seriousness, and relationship to study medication will be described for all study patients. Severity of the AEs will be graded according to NCI-CTCAE Version 3.0.

15.4.2 Dose limiting toxicity (DLT)

Dose limiting toxicity is defined as any of the non-hematological or hematological toxicities defined in section 12.1.6. Those events will be indicated as DLT if they occur after the single dose application within the follow-up period (until Day 15). The proportion of patients with DLT will be tabulated over time.

15.4.3 Clinical laboratory measures

Laboratory data (hematology, biochemistry, coagulation parameters, and urinalysis) will be summarized. Changes from Baseline will be presented using shift tables (employing the categories ‘normal’, ‘abnormal, clinically not significant’ and ‘abnormal, clinically significant’) and absolute changes in laboratory values, if appropriate.

15.4.4 Anti-drug antibodies

Anti-drug antibodies will be analyzed and separately reported by Covance Ltd., Harrogate, United Kingdom.

15.4.5 Electrocardiogram

The results of the 12-lead ECG recordings will be summarized by time point. QT intervals will be corrected using the Bazett formula. Changes from Baseline will be presented using shift tables (employing the categories ‘normal’, ‘abnormal, clinically not significant’ and ‘abnormal, clinically significant’) and absolute changes in laboratory values, if appropriate. Changes from Baseline in QTc between >30 and <60 ms and >60 ms will be listed and summarized separately.

15.4.6 Vital signs

Vital signs will be summarized by time point, along with the changes from Baseline.

15.4.7 Physical examination

The results of any changes in physical examination finding from Baseline will be summarized.

15.5 Pharmacokinetic analysis

Pharmacokinetic parameters as described in Section 13.2 will be analyzed and separately reported by Covance Clinical Research Unit, Leeds, United Kingdom.

15.6 Efficacy Analyses

15.6.1 Biomarkers

Plasma PlGF levels and one additional biomarker of interest in serum will be analyzed and reported separately.

15.6.2 Tumor measurements

RECIST disease response will be summarized. The objective tumor response for target lesions will be presented over time (employing the categories ‘complete response [CR]’, ‘partial response [PR]’, ‘progressive disease [PD]’ and ‘stable disease [SD]’) and absolute changes.

Parameters measured with DCI-MRI as described in section 14.1 will be analyzed by the Magnetic Resonance Development and Application Center, Freiburg, Germany. Changes in DCE-MRI parameters from Screening to Day 2 and Day 43 will be analyzed.

15.6.3 ECOG performance status

ECOG performance status will be tabulated over time. For a specification of ECOG categories refer to section 14.2.

15.7 Interim analysis

A formal interim analysis is not intended for this study. During the process of the study an evaluation of ongoing safety, tolerability and pharmacokinetic data, if available, will be performed to confirm or re-assign the planned doses of Angiocal® to be administered during the dose-escalation part of the study.

16 Patient withdrawal from study participation

Every patient has the right to withdraw his/her consent to participate in the study at any time and without giving reasons. Patients must be withdrawn from the study under the following circumstances:

- If the patient experiences a DLT event during the single or repeated dosing period;
- If, in the investigators opinion, continuation in the clinical study would be detrimental to the patient's well-being;
- The patient wishes to be withdrawn (withdrawal of consent);
- If the patient is lost to follow-up;
- If major protocol violations occur (e.g., non-compliance with study protocol, violation of eligibility criteria) that have significant impact on the evaluation and interpretation of the study data;
- If the sponsor decides to terminate the study.

Whenever possible, the tests and evaluations listed for the Final Visit should be performed. The sponsor should be notified of all study withdrawals as soon as possible and all terminations for adverse events within 72 hours. Whenever a patient is prematurely withdrawn from study participation the date and reason(s) for discontinuation of the study must be documented in the CRF.

Patients who terminate the study prematurely for whatever reason will be treated adequately by the investigator at his discretion and according to the standard treatment guidelines. In case of stable disease and response to treatment at Day 43 the investigator may decide to continue with Angiocal® treatment if the patient agrees. Angiocal® will then be given biweekly starting at Day 57. The Final Visit at Day 71 will not be performed in these patients. The investigator will decide to continue treatment during the follow-up visits every two weeks. After every fourth dose of Angiocal® a CT scan will be performed to determine the progress of the disease. In case of progressive disease no further treatment with Angiocal® will be given.

Patients withdrawn from the study will not be replaced. Reasonable effort should be made to contact any patient lost to follow up during the course of the study, to complete assessments and to retrieve any outstanding data.

17 Ethical and legal requirements

17.1 General requirements

The procedures outlined in the protocol pertaining to the conduct, evaluation, and documentation of this study are designed to ensure that the sponsor, the investigator, and the CRO perform the study in accordance with the Declaration of Helsinki and International Conference on Harmonization (ICH) GCP guidelines. In addition, all local regulatory requirements will be followed.

17.2 Independent ethics committee and competent authority approval

Before the start of the study, the clinical study protocol and its amendments any other appropriate study-related documents will be submitted for review and approval to the relevant ECs and to the CA, as required. Written approval of the study must be obtained from the EC and CA before any patient is enrolled at a center. Substantial amendments to the study protocol will also be forwarded to the EC and CA and need written approval.

17.3 Patient information and consent procedure

Prior to entry into the clinical study, the investigator will explain to each patient the aims, significance, methods, anticipated benefits, and potential hazards of the study. After these explanations and before entering the study, the patient will sign and date an informed consent form and will receive a copy of the signed and dated form. Each patient must be informed that participation in the study is voluntary and that he/she may withdraw from the study at any time and that withdrawal of consent will not affect his/her subsequent medical treatment or relationship with the treating physician. The informed consent forms will be reviewed and approved by the responsible EC prior to initiation of the study.

In the case of amendments to the final protocol that would directly affect the patient's participation in the study (e.g. a change in any procedure), the patient information sheet and informed consent form must be amended to incorporate this modification. Patients who want to continue study participation must agree to sign the amended form indicating that they re-consent to participate in the study.

Any changes to the proposed consent form suggested by the investigator must be agreed to by the sponsor before submission to the EC, and a copy of the approved version must be provided to the sponsor after EC approval.

17.4 Insurance coverage

Insurance coverage for all participating patients is guaranteed according to the country specific legal requirements.

18 Criteria for premature termination of the study and criteria for closing a study center

The sponsor has the right to terminate the entire study when new data become available which raise concern about the safety of the study drug, so that continuation of the study might cause unacceptable risks to patients.

If the study will be terminated prematurely, the patients will not suffer from any disadvantages regarding their medical care.

The sponsor may terminate the study at a study site when the investigator:

- fails to comply with relevant regulations or the principles of GCP;
- insufficiently adheres to protocol requirements;
- enrolls no or very few patients;
- provides data with insufficient quality.

19 Data collection

Electronic CRFs will be used and should be handled in accordance with the instructions provided. eCRFs will be provided as a regulatory compliant, electronically secure and protected, webbased database. An audit trail will record all entries and corresponding changes.

All eCRFs should be filled out completely by authorized study staff. Only authorized persons will be granted access. After the last query for a patient is closed, the eCRF is reviewed by the investigator and signed electronically.

All laboratory data (safety lab sampling as well as PK and biomarker analyses) will be imported electronically secure and protected into the eCRF data base directly from the corresponding laboratories or the study site, respectively.

20 Archiving of data

The investigator will archive all study data (patient identification code list, source data and investigator's file) and relevant correspondence. These documents will be kept according to the requirements of the ICH-GCP guidelines.

The sponsor will archive all study-related documents in the trial master file according to the requirements of the ICH-GCP guideline. Essential documents (as defined in the ICH-GCP guideline) must be retained for 2 years following the last approval of a marketing application in an ICH region; and, until there are no pending or contemplated marketing applications in an ICH region; or, at least 2 years have elapsed since the formal discontinuation of clinical development of the investigational product. According to the German GCP-V study documents must be retained for at least 10 years after the completion of the study. These documents must be retained for a longer period, however, if required by the applicable regulatory requirements or if needed by the sponsor. In addition, the investigator must make provisions for the patient’s medical records to be kept for the same period of time.

No data should be destroyed without the sponsor’s agreement: It is the responsibility of the sponsor to inform the investigator/institution as to when these documents no longer need to be retained. If an investigator leaves an investigational site, the responsibility for archiving of all study-related records has to be transferred to another person (e.g. other investigator). The sponsor has to be informed about any change in responsibility. Patients’ medical records and other original data will be archived in accordance with the archiving regulations or facilities of the investigational sites.

21 Monitoring and quality assurance

Monitoring visits will follow monitoring procedures developed by FGK Clinical Research GmbH, in order to comply with ICH-GCP and to ensure acceptability of the study data for international registration purpose. Regular monitoring visits by representatives of the sponsor and/or entrusted CRO at the investigator’s site prior to the start and during the course of the study will aid in following the study’s progress, ensure utmost accuracy of the data and allow for early detection of possible errors.

Investigators will be extensively trained on how to use the eCRF system. Data will directly be entered into electronic forms and automatically checked for plausibility and completeness during data entry. The clinical research associate will review the CRFs to ensure that they are completed and accurately entered into the database. The clinical research associate will also verify that all data entered in the eCRFs are consistent with source documents. The eCRF database will further be checked for accuracy and consistency by running a series of validated

plausibility-check programs at the data management department at FGK Clinical Research GmbH. Questions identified will be addressed back to the investigator for resolution. Once all questions are resolved the database will be locked.

Protocol deviations discovered during the monitoring visits will be documented in the monitoring visit report and an associated protocol deviation form which will be forwarded to the sponsor for notification and implementation of corrective actions, if necessary.

For quality assurance, the authorities require a direct comparison between the data recorded in the CRF and the source data compiled by the investigator. For this purpose, the investigators will allow the clinical research associate of the sponsor and/or the CRO to inspect the source data compiled for this study.

According to legal requirements, it is also possible that the Quality Assurance Department of FGK Clinical Research GmbH and the sponsor, or a designated contract auditor or a regulatory authority, may want to inspect the logistical procedures as well as to audit the data. The investigator will allow the persons responsible for the audit or the inspection to have access to the source data and documents and will answer any questions.

22 Study report and publications

By signing the clinical study protocol, the investigator agrees that the results of the clinical study may be used for publication. The investigator also agrees that she/he is not permitted to publish data related to the study independent of the sponsor and the other investigators. Any results of medical investigations with the sponsor’s products and all publications, lectures, and manuscripts based thereon shall be exchanged and discussed by the investigator and the sponsor 60 days prior to submission for publication or presentation. The sponsor must be supplied with a copy of any proposed publication. The sponsor’s comments on the publication, including the sponsor’s recommendation as to which publication is most appropriate, will be given without unreasonable delay, and no later than within 60 days. If the sponsor objects to the publication, in whole or in part, or requests to make changes, then the two parties shall agree to change such material as appropriate. If additional time is required in order for the sponsor to ensure protection of its intellectual property the sponsor will notify the publication panel who will delay submission of the abstract or manuscript.

It is understood by the investigator that the information developed in this clinical study will be used by the sponsor and, therefore, may be disclosed by the sponsor as required to other clinical investigators, other pharmaceutical companies, and government agencies. To allow for the use of the information derived from clinical studies, it is understood that there is an obligation to provide to the sponsor complete test results and all data compiled in this study.

23 Study periods

The overall study duration of this clinical study is expected to be approximately 9 to 12 months, with patient recruitment proposed to start in May 2010 and last patient visit occurring in April 2011. The end of the study is defined as the date of the last visit of the last patient in any participating center.

24 References

1. Folkman J. Angiogenesis inhibitors generated by tumors. Mol Med. 1995 Jan;1(2):120-2.
2. Folkman J. Endothelial cells and angiogenic growth factors in cancer growth and metastasis. Introduction. Cancer Metastasis Rev. 1990 Nov;9(3):171-4.
3. Pecorino L. Molecular biology of cancer: mechanisms, targets and therapeutics. 2nd ed. Oxford University Press. 2008.
4. Dvorak HF. Vascular permeability factor/vascular endothelial growth factor: a critical cytokine in tumor angiogenesis and a potential target for diagnosis and therapy. J Clin Oncol 2002. Nov 1;20(21):4368-80.
5. Otrock ZK, Makarem JA, Shamseddine AI. Vascular endothelial growth factor family of ligands and receptors: review. Blood Cells Mol Dis. 2007 May-Jun;38(3):258-68.
6. Tesone M, Stouffer RL, Borman SM, Hennebold JD, Molskness TA. Vascular endothelial growth factor (VEGF) production by the monkey corpus luteum during the menstrual cycle: isoform-selective messenger RNA expression in vivo and hypoxia-regulated protein secretion in vitro. Biol Reprod. 2005 Nov;73(5):927-34.
7. Wu LW, Mayo LD, Dunbar JD, Kessler KM, Baerwald MR, Jaffe EA, et al. Utilization of distinct signaling pathways by receptors for vascular endothelial cell growth factor and other mitogens in the induction of endothelial cell proliferation. J Biol Chem. 2000 Feb 18;275(7):5096-103.
8. Dimmeler S and Zeiher AM. Akt takes center stage in angiogenesis signaling. Circ Res. 2000 Jan 7-21;86(1):24-9.
9. Abedi H and Zachary I. Vascular endothelial growth factor stimulates tyrosine phosphorylation and recruitment to new focal adhesions of focal adhesion kinase and paxillin in endothelial cells. J Biol Chem. 1997 Jun 13;272(24): 15442-15451.
10. Cébe-Suarez S, Zehnder-Fjällman A, Ballmer-Hofer K. The role of VEGF receptors in angiogenesis; complex partnerships. Cell Mol Life Sci. 2006 Mar;63(5):601-15.
11. Ferrara N. VEGF and the quest for tumour angiogenesis factors. Nat Rev Cancer. 2002 Oct;2(10):795-803.
12. Fox E, Curt GA, Balis FM. Clinical Trial Design for Target-Based Therapy. The Oncologist. 2002. Vol. 7, No. 5, 401–409.

25 Changes to protocol Version 1.0

Changes to protocol Version 1.0 included the following:

- Actions to be taken if a patient experiences a DLT were clarified (*changed in Sections 1, 8.1, 8.2.2, 11.7, 11.9, 12.1.6 and 16*):

  Patients who experience a DLT after the single dose application within the follow-up period will not continue into the repeated dosing period. Patients who experience a DLT event during the repeated dosing period will be withdrawn from the study.

- The maximum sample size was changed to 42 patients and the calculation was precisely described (*changed in Sections 1 and 9.1*).
- The system for patient numbering was changed (*changed in Section 10.3*).
- The definition of the first study-related activity was deleted as this text was redundant (*changed in Section 12.1.3*).
- Statistical methods section in the synopsis was simplified (*changed in Section 1*).
- The definition of a DLT in patients with liver metastases was added (*changed in Sections 1 and 12.1.6*):

  The definition of DLT now considers patients with liver metastases who must have a prolonged (>2 weeks) grade 3 toxicity with an increase in ALT and AST >5.0 - 20.0 x ULN.

- The analysis of one additional biomarker of interest was included (*changed in Sections 1, 11.4, 11.5, 11.6, 14.3 and 15.6.1*).
- Spelling mistakes for sponsor's name and address were corrected (*changed in Sections 2 and 25*).

No patient was treated under protocol Version 1.0.

26 Approval and signatures

Study Code: Pieris001
EudraCT No.: 2009-015601-38

This clinical protocol was subject to critical review and has been approved by the sponsor.

The signatories declare,

- that they conduct the clinical study in compliance with all applicable regulations and guidelines as stated in the protocol, including the national law, the Declaration of Helsinki and ICH-GCP guidelines, and other information supplied;
- that they are familiar with the pharmacological and toxicological investigations of the study drug, the results from other studies, and the possible risks of the study;
- that they have read the protocol and agree to conduct the study as outlined.

Sponsor's signatory

Dr. Angelika C. Stern
Head of Project Management Angiogenesis
Pieris AG
Lise-Meitner-Straße 30
85354 Freising
Germany

10.5.2010 — Date
Signature

Coordinating Investigator and Director Clinical Investigation (LKP)

PD Dr. med. Klaus Mross
Klinik für Tumorbiologie an der Albert-Ludwigs-Universität Freiburg
Department of Medical Oncology
Breisacher Str. 117
76106 Freiburg im Breisgau, Germany

Date
Signature

26 Approval and signatures

Study Code: Pieris001
EudraCT No.: 2009-015601-38

This clinical protocol was subject to critical review and has been approved by the sponsor.

The signatories declare,

- that they conduct the clinical study in compliance with all applicable regulations and guidelines as stated in the protocol, including the national law, the Declaration of Helsinki and ICH-GCP guidelines, and other information supplied;
- that they are familiar with the pharmacological and toxicological investigations of the study drug, the results from other studies, and the possible risks of the study;
- that they have read the protocol and agree to conduct the study as outlined.

Sponsor's signatory

Dr. Angelika C. Stern
Head of Project Management Angiogenesis
Pieris AG
Lise-Meitner-Straße 30
85354 Freising
Germany

Date

Signature

Coordinating Investigator and Director Clinical Investigation (LKP)

PD Dr. med. Klaus Mross
Klinik für Tumorbiologie an der Albert-Ludwigs-Universität Freiburg
Department of Medical Oncology
Breisacher Str. 117
76106 Freiburg im Breisgau, Germany

10-05-10
Date

Signature

CRO's signatory

Dr. med. Edgar J. Fenzl
Managing Director/Medical Monitor
FGK Clinical Research GmbH
Heimeranstrasse 35
80339 München, Germany

10.05.10
Date

Edgar Fenzl
Signature

Dr. Monika Schwienbacher
Associate Project Director
FGK Clinical Research GmbH
Heimeranstrasse 35
80339 München, Germany

10-May-2010
Date

Signature

Olaf Böhm
Director Biostatistics & Data Management
FGK Clinical Research GmbH
Heimeranstrasse 35
80339 München, Germany

10 May 2010
Date

O. Böhm
Signature

Principal Investigator

Investigator's name (printed)

Date

Signature

27 Appendix: Protocol MR-Imaging

Protocol

MR-Imaging

Pieris001

Version V1.1 9.03.2010

This protocol was approved by:

**Dr. Martin Büchert**	signature	
**MRDAC**	date	12/5/10
**Dr. Klaus Mross**	signature	
**Study Chairman**	date	17.5.10
**Klinik für Tumorbiologie**		

Table of contents

MR Scanner ... 4
Imaging times ... 4
Lesion guidelines ... 4
Preparation ... 4
Examination protocol ... 6
1. Localizer/ Scout ... 6
2. T1w Gradient Echo (Fl2d) axial ... 6
3. T2w HASTE axial ... 6
4. T1w fat sat Gradient Echo (Fl2d) coronal ... 6
5. Test position for dynamic DCE-MRI scan coronal ... 6
6. T2*_ME_ Flash pre-contrast (optional) ... 7
7. EPI 2D Diffusion scan (ADC-Map) ... 7
8. Dynamic DCE-MRI scan ... 7
9. T1w fat sat Gradient Echo (Fl2d) axial ... 7
10. T1w fat sat Gradient Echo (Fl2d) coronal ... 7
11. EPI 2D Diffusion scan (ADC-Map) ... 7
12. Marked Target Lesion ... 7
QA Measurements ... 10
Data Entry Check ... 11
DCE-MRI Analysis protocol ... 11
Reading of the MR-images ... 13

MR Scanner

Examinations are carried out on a Siemens 1.5T clinical MR-scanner using

- body and spine array coils for best covering of the lesions
- MR-compatible computer controlled contrast media injector.

Imaging times

Number and dates of the MRI scans are according to the Pieris001 study protocol.
MRI scans will include T1- and T2-weighted images of measurable disease and dynamic contrast enhanced (DCE-) MRI, diffusion weighted and T2*-weighted (optional) MRI of one representative lesion (target lesion).

Lesion guidelines

Prior to acquiring DCE-MRI images on a study subject, a target lesion should be identified by a radiologist or an MR technologist under the supervision of a radiologist, who are familiar with the requirements of the Pieris001 study protocol. Below are the recommended lesion selection guidelines following modified RECIST criteria:

- hepatic metastases
- Minimum lesion size is 2cm longest in plane diameter
- Lesion should be well defined on standard anatomical MR Imaging
- Lesion should be non-necrotic and non-cystic

Preparation

Due to new regulations regarding the use of gadolinium-containing MRI contrast agents it is required that at time of MR examination an actual (not older than 14 days) creatinine value of the patient is available.
The patient has to be informed about the repetitive breath holding procedure during some of the sequences and the commands used via the intercom.

Patient positioning: The subject will be placed (supine - on his/her back) on the magnet bed. The patient will be positioned in such a manner that the imaged volume

resides in the center of elements 3+4 of the spine array coil which is integrated into the magnet bed.

Volume of interest: Coronal acquisitions should cover the whole abdomen from anterior to posterior, at least the whole liver. Axial acquisitions also have to cover to whole liver in all three directions.

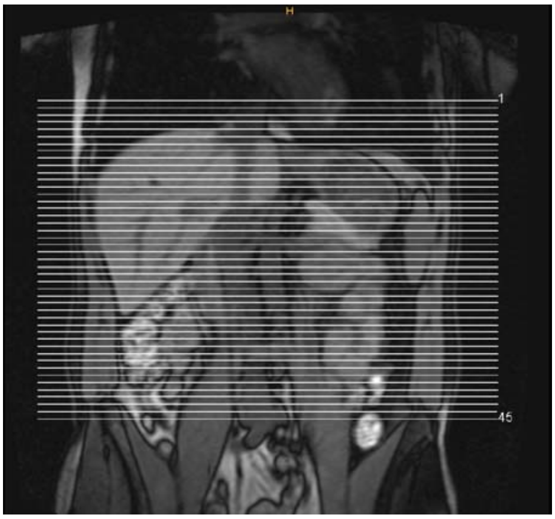

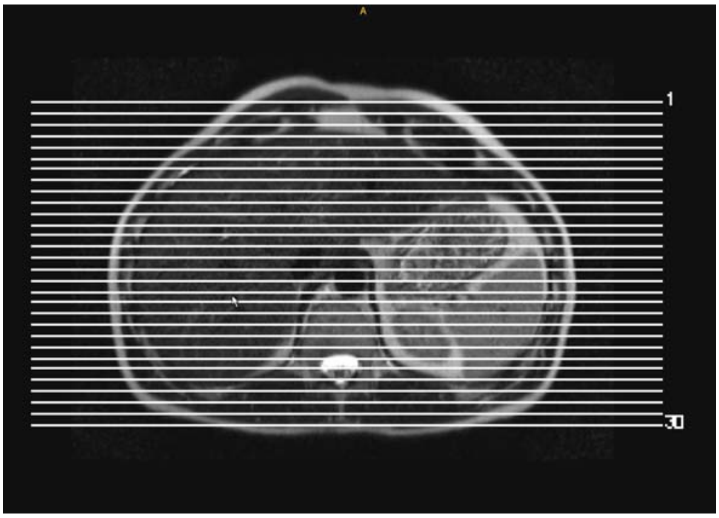

For cases were the above mentioned criteria's can not be matched with the default FOV and/or number of slices listed in the table below, these values may be increased. This could be rarely the case for overweight patients.
This volume of interest definition does not apply to the localizer and the IR-TrufiFisp measurements. Details about their positioning are described in the following examination protocol section.

Contrast injection: Gd-based contrast agent is administered into the right (left if not possible) antecubital vein

Contrast media: low molecular Gd-based MRI-CA like Multihance©.

Saline: 30ml.

Injection rate: 3 ml/s (w/ power injector).

The power injector is preset with an injection delay of 36 seconds (12 measurements of the dynamic scan), and the injector is started concurrently with start of the MR-scan. Injection is commenced after the first 12 measurements to allow a steady state to develop and have a sufficient range for baseline calculation.

Examination protocol

1. Localizer/ Scout

A localizer scan is performed for proper slice positioning of succeeding scans.

Localizers are acquired in breath hold mode (inspiration) as basis for positioning of the T1- and T2-weighted image scans.

2. T1w Gradient Echo (Fl2d) axial

The whole volume of interest is imaged. In the case of a liver metastasis, the whole liver is imaged with 5mm slices. This can not be done in a single breath-hold. The breath-hold commands are repeated. Number of breath-holds and total number of slices is controlled by the measurement parameter Concatenations.

Quick Fat sat pulse for fat suppression is applied. All the images where breath hold is necessary are acquired in inspiration.

3. T2w HASTE axial

T2 weighted images are acquired in the same manner as the T1w GRE scans. The complete volume of interest is imaged. Number of Concatenations is set to image i.e. the whole liver. The position of the imaging volume is copied from the T1w scan.

4. T1w fat sat Gradient Echo (Fl2d) coronal

A T1w scan is performed in coronal view. The whole volume of interest is imaged with 6 mm slices. The number of breath-hold commands is controlled by the measurement parameter concatenations.

The supervising radiologist or the MR technologist under supervision of the radiologist will then plan a coronal slice for the dynamic study. If there is a choice of multiple suitable lesions according to the study protocol the one in the region of least movement will be chosen. This will remain the target lesion throughout the trial.

5. Test position for dynamic DCE-MRI scan coronal

The T1 weighted multi TI inversion recovery TrueFISP sequence is used for the DCE-MRI dynamic scan. For the test scan number of measurements is set to 3. The coronal slice will be placed in such a way that the target lesion is cut in its biggest extension. The saturation slice, through which the inversion pulse is played out, has to be centered on the very same position as the imaging slice. Frequency offset may be adjusted to optimize imaging quality. Test measurements are repeated until positioning and image quality is satisfying.

6. T2*_ME_ Flash pre-contrast (optional)

T2* multi echo Flash scan is carried out with identical slice position in correspondence to the dynamic scan. Measurement is done in breath-hold. During the scan 12 echoes with echo times in the range of 5ms to 60ms are acquired.

7. EPI 2D Diffusion scan (ADC-Map)

The diffusion weighted scans are carried out with identical slice position in correspondence to the dynamic scan. Measurement is done in breath-hold. Diffusion weighted images are acquired with various b-values in 3 orthogonal directions and with two repetitions.

8. Dynamic DCE-MRI scan

The DCE-MRI dynamic scan is identical to the test scan described under 5. apart from setting number of measurements to 110.

The dynamic contrast enhanced sequence has duration of 5½ minutes. The scan is therefore done during gentle breathing. Start contrast media injector and MR sequence does take place concurrently. See remarks under ‘contrast injection’!

9. T1w fat sat Gradient Echo (Fl2d) axial

A post contrast repetition of the axial pre-contrast T1w Gradient Echo measurement described under 2. with additional fat saturation.

10. T1w fat sat Gradient Echo (Fl2d) coronal

A post contrast T1w Gradient Echo measurement in coronal slice orientation.

11. EPI 2D Diffusion scan (ADC-Map)

Dito 7. post contrast.

Additional data series without extra measurement

12. Marked Target Lesion

There is need of an additional series holding a copy of at least one image from ‘8. Dynamic DCE-MRI scan’. In this/these image/images the target lesion of the DCE-MRI measurement has to be unambiguously marked/circled.

Each examination has to be documented on the following examination protocol form.

DCE-MRI Untersuchungsprotokoll Pieris001

Patienten Daten

Patienten ID / Label			Untersuchungsdatum	
Geburtsdatum			Patientengewicht	kg
Lokalisation IV - Zugang	left	right	Kanülengröße/Farbe	
Kontrastmittel Typ			KM Vol.	ml
Injektionsrate	ml/s		NaCl Vol.	30 ml
Operator			Datensicherung CD	
Radiologe			Kreatinin/Clearance	/

MR-Examination

Serie #	Bilder-anzahl	Sequenz / Parameter / Bemerkungen
		Localizer/Scout
		T1w Gradient Echo (Fl2d) axial
		T2w HASTE axial
		T1w fat sat Gradient Echo (Fl2d) coronal
		Test measurements T1_IRTrufi
		T2*_ME_ Flash pre-contrast
		EPI 2D Diffusion scan (ADC-Map)
		**T1_IRTrufi 110 Measurements KM Gabe**
		T1w fat sat Gradient Echo (Fl2d) axial
		T1w fat sat Gradient Echo (Fl2d) coronal
		EPI 2D Diffusion scan (ADC-Map)
		Serie/n mit markierter Target Läsion

Anmerkungen:

V. 1.0 dt. 09.03.2010

Parameters overview for all sequences:

Sequence	T1 / FLASH 2D	T2 / HASTE	IR-TrueFisp	T2*-Bold	T1 / FLASH 2D	T1 / FLASH 2D	Diffusion / EPI 2D
Orientation	axial	axial	cor	cor	axial	cor	cor
fatsat	no	no	no	no	yes	yes	yes
TE (ms)	4.8	107	1,24	5.1 – 58.9	2.47	2.47	71
TR (ms)	99	1000	3000	65	107	84	900
Bandwidth (Hz/ Pixel)	140	446	950	292	260	260	1628
Flip Angle	80	150	40	30	70	70	-
Magn. prep.	None	None	Sel IR	None	None	None	None
Slices: #	45*	45*	1	1	45*	36*	3 - 7
thickness (mm)	5	5	10	10	5	6	5 / 10
Gap (mm)	0.5	0.5	-	-	0.5	0.6	-
Quick sat.	no	no	no	no	yes	yes	no
Base Resolution	256	320	128	256	256	256	128
Phase Resolution	80%	80%	100%	100%	75%	70%	100%
Phase partial Fourier	Off	5/8	5/8	Off	Off	Off	6/8
Interpolation	no	no	no	no	yes	Yes	no
FOV	350*	350*	400*	400*	350*	400*	400*
pFOV	75%	75%	100%	75%	75%	100	100%
TA (s)	76	45	9 / 330	14	108	75	17
Measurements	1	1	3 / 110	1	1	1	1
Excitation order interleaved	yes	yes	-	-	yes	yes	-
concatenations	5	3	1	1	7	5	1
Voxel (mm^3)	1.7 x 1.4 x 5.0	1.4 x 1.1 x 5.0	3.1 x 3.1 x 10	1.6 x 1.6 x 10	2.1 x 1.4 x 6.0	2.2 x 1.6 x 6.0	3.1 x 3.1 x 10

*FOV and number of slices may be increased if necessary to meet the criteria's given in the 'volume of interest' section. Sequences and parameters are subject to changes if required by security or technical reasons.

QA Measurements

Freiburg

QA measurements and documentation on our MR scanners are carried out on a regularly basis (weekly) according to manufacturers guidelines.

Third party sites

Within multi center studies each site has to take care of appropriate QA measurements and documentation.

Test Measurements

Prior the first patient study examination each site has to perform a volunteer examination following the above-described MR protocol except of the contrast media application. This data will be sent to MRDAC, where it will be checked regarding MR protocol conformity. After a positive report confirming that the protocol is in agreement with the above described protocol the site may start with patient examinations.

Data Management

Freiburg

MRI data will be saved on a local PACS system.

In addition CDs with DICOM image data will be burned for back up reasons. Data will be stored in accordance to legal regulations for medical data.

MRDAC will perform a data entry check within 48h after examination to check MR protocol conformity of the data.

Third party sites

DICOM images and the corresponding examination protocol form will be sent on the same day or not later than 24h after examination on CD/DVD to MRDAC. Alternatively it may be agreed on an electronic transfer of the data.

MRDAC will perform a data entry check within 48h after arrival to check MR protocol conformity of the data. A notification by email regarding the data entry check result will be sent via email from MRDAC to the centre which acquired the data.

Data Entry Check

The intention of this first data check is to evaluate formal conformity of the acquired data with the MR examination protocol.

This includes the following points

- Completeness of data with respect to
  - o number of series yes/no
  - o number of slices per series yes/no
- Coverage of volume of interest yes / no
- Contrast application yes/no
- Inversion recovery contrast in DCE-MRI measurement yes / no
- Patient motion acceptable / not acceptable
- General artifact level acceptable / not acceptable
- Parameter conformity of all sequences regarding crucial parameters

A more detailed check of the IR-TrueFisp series with its more than 700 individual images is beyond this entry check and is intrinsic part of the subsequent analysis.

DCE-MRI Analysis protocol

A dedicated custom-built software package developed under Matlab (http://www.mathworks.com) is used for the quantitative analysis of DCE-MRI data. This software package runs on external work stations and is implemented with a user-friendly graphical user interface to minimize user interaction. The data processing consists of several steps for the accurate determination of concentration values and physiologic parameters.

1) A region-of-interest (ROI) spanning the target lesion (see Lesion guidelines above) is defined in pre-contrast images of the dynamic series with the help of coronal anatomical T1w images. The chosen ROI is reviewed by the responsible radiologist. The ROIs are then semi-automatically registered for all time frames of the dynamic series using a correlation analysis-based algorithm. To minimize through-plane movement during breathing the 2D slices are acquired in a typically coronal oriented view. Registered ROIs undergo a visual inspection.

2) Mean R_1=1/T_1 values for the whole tumor are calculated from image signal intensities according to the formulas published by [Scheffler 2003].

3) Concentration values are calculated straightforward from the R_1 values according to the expression

$$C(t) = (R_1 - R_{10}) / r_1 , \qquad (0.1)$$

where e.g. for Gd-DTPA r_1=4.3 mmol/l*s is the relaxivity taken from literature and R_{10} is the baseline R_1 relaxation rate before contrast arrival [Rohrer 2005].

4) Pharmacokinetic modeling is done using the multi-compartment model of Tofts [Tofts and Kermode 1991]:

$$\frac{dc_t(t)}{dt} = K^{trans} * c_p(t) - k_{ep} * c_t(t) , \qquad (0.2)$$

where c_t is the concentration in tissue and c_p is the concentration in blood plasma. k_{ep} is the so-called reflux constant and is related to K^{trans} as $k_{ep} = K^{trans} / Ve$.

A standardized bi-exponential function serves as an arterial input function as defined by the following expression [Weinmann, Laniado et al. 1984, Tofts and Kermode 1991]: $c_p = D[a_1 \exp(-m_1 t) + a_2 \exp(-m_2 t)]$,

where a_1= 3.99 kg/l, a_2= 4.78 kg/l, m_1= 0.144 min^{-1} and m_2= 0.011 min^{-1}.

5) The initial area under curve (iAUC) was introduced by Evelhoch as an additional model-free DCE-MRI parameter [Evelhoch 1999]. The parameter iAUC60 is calculated as the area under the Gd concentration time curve for the first 60s after the onset of CA uptake. In addition, iAUC90 and iAUC120 are calculated.

6) A pixel wise analysis is done after ROI based analysis is completed. Results are saved in pixel wise format enabling histogram analysis and display of parameter maps for K^{trans} and iAUC60 (as well Ve, iAUC90, iAUC120). Chi square as an estimate of uncertainty of the fitting process is saved and can be included in further analysis. Fit failures can be classified.

References

Evelhoch, J. L. (1999). "Key factors in the acquisition of contrast kinetic data for oncology." J Magn Reson Imaging **10**(3): 254-9.

Scheffler, K. (2003). "On the transient phase of balanced SSFP sequences" Magn Reson Med **49**:781-3

Rohrer, M. (2005). "Comparison of magnetic properties of MRI contrast media solutions at different magnetic field strength" Invest Radiol **40**:715-24

Tofts, P. S. and A. G. Kermode (1991). "Measurement of the blood-brain barrier permeability and leakage space using dynamic MR imaging. 1. Fundamental concepts." Magn Reson Med **17**(2): 357-67.

Weinmann, H. J., M. Laniado, et al. (1984). "Pharmacokinetics of GdDTPA/dimeglumine after intravenous injection into healthy volunteers." Physiol Chem Phys Med NMR **16**(2): 167-72.

Reading of the MR-images

Freiburg

Experienced radiologists of the Radiological Department, University Medical Centre Freiburg, will read the MRI data and measure lesion sizes in the covered part of the upper abdomen according to modified RECIST criteria. All examinations of one patient should be read by the same radiologist. If the radiologist is at the day of one examination not at duty, he has to read the MRI data as soon as possible but at the latest at the third day after the examination. If this is not possible, because of longer absence of the radiologist, the MRI data can also be read by another radiologist. For each examination a reading formula (see next page) will be filled and sent per Email to the clinic sending the patients.

All the MR-examinations and readings performed by MRDAC are designed especially for the requirements of the study and cannot replace a standard diagnostic examination.

Third party sites (optional)

MRDAC can provide the same service described above for data acquired at third party sites.

Universitätsklinikum Freiburg Abt. Röntgendiagnostik
Ärztlicher Direktor Prof.Dr.med. M. Langer **Wiss. Direktor** Prof.Dr.rer.nat. J. Hennig
Hugstetter Str. 55, 79106 Freiburg

Klinik für Tumorbiologie Freiburg
Email an: dce-mrt@tumorbio.uni-freiburg.de
Kopie an: aag.mr@uniklinik-freiburg.de

Vorläufiger Kurzbefund

Patientenname: ____________________ **Studiennr:** ____________

Geburtsdatum: **Untersuchungsdatum:** ____________

MR-Dynamik: ☐ mit DCE ☐ ohne DCE **Start KM-Injektion:** ____________ **Uhr**

Tumorlage der **Referenzläsion** (RL):	
Tumorgröße der RL in der Voruntersuchung (VU):	
Tumorgröße der RL aktuell:	0,00
Gesamtdiameter der Target-Läsionen in VU:	
Gesamtdiameter der Target-Läsionen aktuell:	0,00
Non-Target-Läsionen:	
Neue Metastasen (Wenn ja, wo):	

Target Läsionen

Nr.	Organ	Serien-Nr. S/ Bild-Nr. B	Längster Durchmesser
1 (RL)			cm
2			cm
3			cm
4			cm
5			cm
6			cm
7			cm
8			cm
9			cm
10			cm
		**Summe der längsten Durchmesser:**	**0,00**

Non-Target Läsionen

Nr.	Organ	Serien-Nr. S/ Bild-Nr. B	Präsenz= 1 / Absenz= 0
1			
2			
3			
4			
5			
6			
7			
8			
9			
10			

Datum: 11.07.2008 **Arzt:** ____________________

V1.8. v.12/07

Amendment 1

Amending protocol Version 2.0 of 10-May-2010

A PHASE I, OPEN-LABEL STUDY OF THE SAFETY, TOLERABILITY, AND PHARMACOKINETICS OF ANGIOCAL® (PRS-050-PEG40) IN PATIENTS WITH SOLID TUMORS

Protocol code: Pieris001

EudraCT No.: 2009-015601-38

Sponsor: **Pieris AG**
Lise-Meitner-Straße 30
85354 Freising
Germany

Amendment Version: **Final 1.0**
Amendment Date: **11-June-2010**

TABLE OF CONTENTS

Page

1 OVERVIEW OF CHANGES 3

2 PROTOCOL SECTIONS CHANGED 3

2.1 Section 10.1.3 (Changes 1 and 2) 3

2.2 Section 11.1 (Change 3) 4

3 APPROVAL OF AMENDMENT 1 5

1 OVERVIEW OF CHANGES

The following changes to the clinical study protocol Version 2.0 are made:

Change 1:

The time specified in the protocol for Angiocal® administration will be given as guidance only, as the volume of the Angiocal® solution at low dose levels may be too small to be administered within 3 to 5 minutes.

Change 2:

To ensure a complete intravenous administration of Angiocal®, the injection of 10 mL of a 0.9% of sodium chloride solution immediately after Angiocal® administration is included in the protocol.

Change 3:

For consistency with other sections of the protocol, a clarification on the sequential treatment assignment is introduced in Section 11.1.

2 PROTOCOL SECTIONS CHANGED

The protocol sections that are changed are detailed below. Bolded text in the “new text” is used to indicate the addition, crossed out text in the “original text” indicates the deletion of information from the original text.

2.1 Section 10.1.3 (Changes 1 and 2)

Original text:

Angiocal® is to be administered intravenously by a slow bolus injection over 3 to 5 minutes. The volume to be administered to a patient is calculated according to handling instructions. Patients will receive 5 i.v. injections, one single treatment and 4 treatments during the repeated dosing period. During the repeated dosing period study medication will be administered once weekly for 4 weeks on Days 22, 29, 36, and 43.

New text:

Angiocal® is to be administered intravenously by a slow bolus injection **preferably** over 3 to 5 minutes. The volume to be administered to a patient is calculated according to handling instructions. Patients will receive 5 i.v. injections, one single treatment and 4 treatments during the repeated dosing period. During the repeated dosing period study medication will be administered once weekly for 4 weeks on Days 22, 29, 36, and 43. **After each**

administration of Angiocal® 10 mL of a 0.9% sodium chloride solution will be administered intravenously.

2.2 Section 11.1 (Change 3)

Original text:

Patient enrollment will be done sequentially. Each dose level cohort will consist of three patients. Only one patient will be treated first, the next two patients will be treated after 48 h of follow-up of the previously treated patient.

New text:

Patient enrollment will be done sequentially. Each dose level cohort will consist of three patients. Only one patient will be treated first, the next two patients will be treated after 48 h of follow-up **(Day 3)** of the previously treated patient.

3 APPROVAL OF AMENDMENT 1

Version: Final 1.0

Date: 11-June-2010

Study code: Pieris001

Sponsor: Pieris AG, Freising, Germany

The signatories declare, that they have read the protocol Amendment and that they agree with its contents.

Sponsor's signatory

Dr. Angelika C. Stern
Head of Project Management Angiogenesis
Pieris AG
Lise-Meitner-Straße 30
85354 Freising
Germany

11. June 2010 — Date

[signature] — Signature

Coordinating Investigator and Director Clinical Investigation (LKP)

PD Dr. med. Klaus Mross
Klinik für Tumorbiologie an der Albert-Ludwigs-Universität Freiburg
Department of Medical Oncology
Breisacher Str. 117
76106 Freiburg im Breisgau, Germany

Date

Signature

3 APPROVAL OF AMENDMENT 1

Version: Final 1.0

Date: 11-June-2010

Study code: Pieris001

Sponsor: Pieris AG, Freising, Germany

The signatories declare, that they have read the protocol Amendment and that they agree with its contents.

Sponsor's signatory

Dr. Angelika C. Stern
Head of Project Management Angiogenesis
Pieris AG
Lise-Meitner-Straße 30
85354 Freising
Germany

Date ____________ Signature ____________

Coordinating Investigator and Director Clinical Investigation (LKP)

PD Dr. med. Klaus Mross
Klinik für Tumorbiologie an der Albert-Ludwigs-Universität Freiburg
Department of Medical Oncology
Breisacher Str. 117
76106 Freiburg im Breisgau, Germany

11.6.10
Date ____________ Signature ____________

CRO's signatory

Dr. med. Edgar J. Fenzl<br>Managing Director/Medical Monitor<br>FGK Clinical Research GmbH<br>Heimeranstrasse 35<br>80339 München, Germany	28.06.10<br>Date	Signature
Dr. Monika Schwienbacher<br>Associate Project Director<br>FGK Clinical Research GmbH<br>Heimeranstrasse 35<br>80339 München, Germany	11-Jun-2010<br>Date	Signature
Olaf Böhm<br>Director Biostatistics & Data Management<br>FGK Clinical Research GmbH<br>Heimeranstrasse 35<br>80339 München, Germany	14 Jun 2010<br>Date	O. Böhm<br>Signature

Principal Investigator

[illegible]<br>Investigator's name (printed)	17.6.10.<br>Date	Signature

Amendment 2

Amending protocol Version 2.0 of 10-May-2010

A PHASE I, OPEN-LABEL STUDY OF THE SAFETY, TOLERABILITY, AND PHARMACOKINETICS OF ANGIOCAL® (PRS-050-PEG40) IN PATIENTS WITH SOLID TUMORS

Protocol code: Pieris001

EudraCT No.: 2009-015601-38

Previous amendments: **Amendment 1 of 11-Jun-2010**

Sponsor: **Pieris AG**
Lise-Meitner-Straße 30
85354 Freising
Germany

Amendment Version: **Final 1.0**
Amendment Date: **22-June-2010**

TABLE OF CONTENTS

Page

1 OVERVIEW OF CHANGES 3
2 PROTOCOL SECTIONS CHANGED 3
2.1 Sections 1 and 12.1.6 3
2.2 Sections 1 and 9.2 4
3 APPROVAL OF AMENDMENT 2 5

1 OVERVIEW OF CHANGES

As long-term toxicities are common side effects of radiation therapy and chemotherapy, patients with persistent grade 1 and 2 toxicities may be included in the study. Inclusion criterion #6 and the definition of dose limiting toxicity (non-hematological toxicity) will be adapted accordingly.

2 PROTOCOL SECTIONS CHANGED

The protocol sections that are changed are detailed below. Bolded text in the "new text" is used to indicate the addition of information to the original text.

2.1 Sections 1 and 12.1.6

Original text:

Non-hematological toxicity

- Any grade 3 or 4 toxicity, excluding nausea and vomiting, which responds to antiemetic treatment and alopecia;
- Prolonged (>2 weeks) grade 2 toxicities including serum creatinine 1.5 - 3 x ULN or calculated creatinine clearance <60 mL/min, increase in ALT, AST, alkaline phosphatase >2.5 - 5.0 x ULN, total bilirubin >1.5 - 3.0 ULN, intention tremor, slurred speech, nystagamus, or dysmetria. Exception: patients with liver metastases at screening must have a prolonged (>2 weeks) grade 3 toxicity with an increase in ALT and AST >5.0 - 20.0 x ULN.

New text:

Non-hematological toxicity

- Any grade 3 or 4 toxicity, excluding nausea and vomiting, which responds to antiemetic treatment and alopecia;
- Prolonged (>2 weeks) grade 2 toxicities including serum creatinine 1.5 - 3 x ULN or calculated creatinine clearance <60 mL/min, increase in ALT, AST, alkaline phosphatase >2.5 - 5.0 x ULN, total bilirubin >1.5 - 3.0 ULN, intention tremor, slurred speech, nystagamus, or dysmetria. Exception**s**: patients with liver metastases at screening must have a prolonged (>2 weeks) grade 3 toxicity with an increase in ALT and AST >5.0 - 20.0 x ULN**; patients with persistent grade 1 and 2 toxicities induced by previous anticancer therapy must have a prolonged (>2 weeks) grade 3 toxicity**.

2.2 Sections 1 and 9.2

Original text:

Inclusion criteria:

1. Males or females with pathologically confirmed diagnosis of advanced, recurrent or metastatic cancer, refractory to standard therapy. Patients with tumors for which no standard therapy exists will also be eligible;
2. Age ≥18 years;
3. Measurable or non-measurable disease according to RECIST, as determined by the investigator;
4. ECOG performance status 2;
5. Estimated life expectancy of at least 3 months;
6. No current toxicities due to previous anticancer therapy (radiation therapy, chemotherapy, or surgery);
7. Signed informed consent form and ability to understand the study procedures.

New text:

Inclusion criteria:

1. Males or females with pathologically confirmed diagnosis of advanced, recurrent or metastatic cancer, refractory to standard therapy. Patients with tumors for which no standard therapy exists will also be eligible;
2. Age ≥18 years;
3. Measurable or non-measurable disease according to RECIST, as determined by the investigator;
4. ECOG performance status 2;
5. Estimated life expectancy of at least 3 months;
6. No current **acute** toxicities due to previous anticancer therapy (radiation therapy, chemotherapy, or surgery); **patients with persistent grade 1 and 2 toxicities induced by previous anticancer therapy may be included;**
7. Signed informed consent form and ability to understand the study procedures.

3 APPROVAL OF AMENDMENT 2

Version: Final 1.0

Date: 22-June-2010

Study code: Pieris001

Sponsor: Pieris AG, Freising, Germany

The signatories declare, that they have read the protocol Amendment and that they agree with its contents.

Sponsor's signatory

Dr. Angelika C. Stern
Head of Project Management Angiogenesis
Pieris AG
Lise-Meitner-Straße 30
85354 Freising
Germany

22. June 2010 Date

[signature] Signature

Coordinating Investigator and Director Clinical Investigation (LKP)

PD Dr. med. Klaus Mross
Klinik für Tumorbiologie an der Albert-Ludwigs-Universität Freiburg
Department of Medical Oncology
Breisacher Str. 117
76106 Freiburg im Breisgau, Germany

Date

Signature

3 APPROVAL OF AMENDMENT 2

Version: Final 1.0

Date: 22-June-2010

Study code: Pieris001

Sponsor: Pieris AG, Freising, Germany

The signatories declare, that they have read the protocol Amendment and that they agree with its contents.

Sponsor's signatory

Dr. Angelika C. Stern
Head of Project Management Angiogenesis
Pieris AG
Lise-Meitner-Straße 30
85354 Freising
Germany

Date ________ Signature ________

Coordinating Investigator and Director Clinical Investigation (LKP)

PD Dr. med. Klaus Mross
Klinik für Tumorbiologie an der Albert-Ludwigs-Universität Freiburg
Department of Medical Oncology
Breisacher Str. 117
76106 Freiburg im Breisgau, Germany

22.6.10
Date

[signature]
Signature

CRO's signatory

Dr. med. Edgar J. Fenzl<br>Managing Director/Medical Monitor<br>FGK Clinical Research GmbH<br>Heimeranstrasse 35<br>80339 München, Germany	28.06.10<br>Date	Signature
Dr. Monika Schwienbacher<br>Associate Project Director<br>FGK Clinical Research GmbH<br>Heimeranstrasse 35<br>80339 München, Germany	22-Jun-2010<br>Date	Signature
Olaf Böhm<br>Director Biostatistics & Data Management<br>FGK Clinical Research GmbH<br>Heimeranstrasse 35<br>80339 München, Germany	22 Jun 2010<br>Date	Signature

Principal Investigator

Investigator's name (printed)	Date	Signature

Amendment 3

Amending protocol Version 2.0 of 10-May-2010

A PHASE I, OPEN-LABEL STUDY OF THE SAFETY, TOLERABILITY, AND PHARMACOKINETICS OF ANGIOCAL® (PRS-050-PEG40) IN PATIENTS WITH SOLID TUMORS

Protocol code: Pieris001

EudraCT No.: 2009-015601-38

Previous amendments: Amendment 1 of 11-Jun-2010
Amendment 2 of 22-Jun-2010

Sponsor: Pieris AG
Lise-Meitner-Straße 30
85354 Freising
Germany

Amendment Version: Final 1.0
Amendment Date: 10-Sep-2010

TABLE OF CONTENTS

Page

1 OVERVIEW OF CHANGES 3

2 APPROVAL OF AMENDMENT.. 4

1 OVERVIEW OF CHANGES

This amendment includes one major and several minor changes.

Major change:

Changes in DLT definition: Since patients with controlled hypertension are not excluded from this study, grade 3 hypertension (defined as "requiring more than one drug or more intensive therapy than previously") will be excluded from the definition of DLT, to allow adjustments in hypertension medication if necessary. Furthermore, an event has to be drug-related to qualify for the definition of a DLT.

Bolded text in the new definition is used to indicate the addition, crossed out text in the original definition indicates the deletion of information from the original text.

Old definition:

DLT is defined as any of the following clinical toxicities, referencing National Cancer Institute Common Toxicity Criteria for AEs (NCI-CTCAE version 3.0):

Non-hematological toxicity

- Any grade 3 ~~or 4~~ toxicity, excluding nausea ~~and~~ vomiting~~,~~ which respond to ~~antiemetic treatment and alopecia~~;

…………

New definition:

DLT is defined as any of the following clinical toxicities, referencing National Cancer Institute Common Toxicity Criteria for AEs (NCI-CTCAE version 3.0), **if not considered to be unrelated to study drug administration by the investigator**:

Non-hematological toxicity

- Any grade 3 toxicity, excluding nausea**,** vomiting **and hypertension** which respond to **adequate therapy at the discretion of the investigator**;
- **Any grade 4 toxicity (excluding nausea and vomiting which respond to adequate therapy);**

…………

Further minor changes:

If no safety concerns arise, during the repeated dosing period the pulse rate and blood pressure measurements at 4 hours and 8 hours post-dose may now be optionally performed at the discretion of the investigator.

The time window for the collection of blood samples pre-dose will be deleted. The time window for the collection of blood samples 8, 24 and 48 hours post-dose will be extended from 5 minutes to 2 hours.

Blood sampling for the assessment of anti-drug antibody response may be performed at Day 1 instead of sampling at Screening.

2 APPROVAL OF AMENDMENT

Version: Final 1.0

Date: 10-Sep-2010

Study code: Pieris001

Sponsor: Pieris AG, Freising, Germany

The signatories declare, that they have read the protocol Amendment and that they agree with its contents.

Sponsor's signatory

Dr. Angelika C. Stern
Head of Project Management Angiogenesis
Pieris AG
Lise-Meitner-Straße 30
85354 Freising
Germany

September 10, 2010
Date

Signature

Coordinating Investigator and Director Clinical Investigation (LKP)

PD Dr. med. Klaus Mross
Klinik für Tumorbiologie an der Albert-Ludwigs-Universität Freiburg
Department of Medical Oncology
Breisacher Str. 117
76106 Freiburg im Breisgau, Germany

10.9.10
Date

Signature

CRO's signatory

Dr. med. Edgar J. Fenzl<br>Managing Director/Medical Monitor<br>FGK Clinical Research GmbH<br>Heimeranstrasse 35<br>80339 München, Germany	22.09.10<br>Date	[signature]<br>Signature
Dr. Monika Schwienbacher<br>Associate Project Director<br>FGK Clinical Research GmbH<br>Heimeranstrasse 35<br>80339 München, Germany	27.09.2010<br>Date	[signature]<br>Signature

Principal Investigator

Investigator's name (printed)	Date	Signature

Amendment 4

Amending protocol Version 2.0 of 10-May-2010

A PHASE I, OPEN-LABEL STUDY OF THE SAFETY, TOLERABILITY, AND PHARMACOKINETICS OF ANGIOCAL® (PRS-050-PEG40) IN PATIENTS WITH SOLID TUMORS

Protocol code: Pieris001

EudraCT No.: 2009-015601-38

Previous amendments: **Amendment 1 of 11-Jun-2010**
Amendment 2 of 22-Jun-2010
Amendment 3 of 10-Sep-2010

Sponsor: **Pieris AG**
Lise-Meitner-Straße 30
85354 Freising
Germany

Amendment Version: **Final 1.0**
Amendment Date: **15-Nov-2010**

TABLE OF CONTENTS

Page

1 OVERVIEW OF CHANGES 3

2 PROTOCOL SECTIONS CHANGED 4

2.1 Section 1 (Change 1) 4

2.2 Section 1 (Change 1 and 2) 4

2.3 Section 11.4, 11.5.1, and 11.5.2 (Change 1) 5

2.4 Section 11.6 (Change 1) 6

2.5 Section 14.3 (Change 1 and 2) 6

2.6 Section 15.6.1 (Change 1) 6

2.7 Section 13.1 (Change 2) 7

2.8 Section 1 (Change 3) 7

2.9 Section 11.4 and 11.6 (Change 3) 7

2.10 Section 12.5 (Change 3) 8

3 APPROVAL OF AMENDMENT 9

1 OVERVIEW OF CHANGES

The following changes to the clinical study protocol Version 2.0 are made:

Change 1:

The analysis of additional potential biomarkers will be included. There are no changes in the collection of blood samples.

A number of biomarkers have been studied by other investigators but none of these predict response to treatment with anti-VEGF pathway inhibitors, although some analytes do change concentration under treatment. The new OncologyMAP™ multiplex panel recently released by Rules Based Medicine Inc. (Austin, TX, USA) facilitates the analysis of a wide range of oncology biomarkers implicated during the use of other cancer treatments. This type of analysis makes the best use of the limited sample volume, requiring only 0.5 mL of matrix for the determination. The aim is to identify analytes that change in concentration in response to Angiocal® rather than relying exclusively on what other investigators have reported with inhibitors of the same pathway.

Change 2:

The measurement of VEGF-A in plasma will be included. There are no changes in the collection of blood samples.

Angiocal® acts by sequestering VEGF-A, thus preventing its interaction with cognate membrane receptors. In order to determine whether this target engagement has been achieved at a given dose and within a given time interval, the remaining free VEGF-A level will be determined. Monitoring target saturation in this manner is crucial as VEGF-A is increased in the presence of other inhibitors of this pathway and this may also occur with Angiocal®. Therefore it is not possible to predict target engagement levels based on Angiocal® pharmacokinetic (PK) levels. Pieris AG is also in the process of establishing a complementing assay designed to measure the levels of the Angiocal® -VEGF complex. Analysis is planned to be performed at Pieris AG on PK sample aliquots following PK assays at Covance Ltd. (Harrogate, United Kingdom) only when these are no longer required for PK measurements.

Change 3:

Vital signs, as defined in Section 12.5 of the protocol, always include the measurement of heart rate, blood pressure, and temperature. For consistency reasons corresponding sections will be adapted accordingly.

2 PROTOCOL SECTIONS CHANGED

The protocol sections that are changed are detailed below. Bolded text in the "new text" is used to indicate the addition, crossed out text in the "original text" indicates the deletion of information from the original text.

2.1 Section 1 (Change 1)

Original text:

Efficacy

- …
- Biomarkers: ~~PlGF (placental growth factor) and one additional biomarker of interest~~;
- …

New text:

Efficacy

- …
- Biomarkers: **potential cancer-associated biomarkers in serum using the Human OncologyMAP™ (Rules-Based Medicine, Inc.)**;
- …

2.2 Section 1 (Change 1 and 2)

Original text:

Page 11:

…						
Blood samples for PK		x[5]	x	x	x	x
…						

Please see next page for footnotes

Page 12:

…						
Blood samples for PK	x	x	x	x	x	
…						

At Days 1, 22, 29, 36, and 43 all assessments will be performed pre-dose if not otherwise noted. AEs/toxicity will be recorded pre- and post-dose.

[1] To be performed within 28 days prior to Day 1.

[2] Taken during 30 days prior to study start.

[3] Pulse rate and blood pressure will be assessed pre-dose and 30 min, 1 h, 2 h, 4 h and 8 h post-dose.

[4] Biomarkers: ~~PlGF and one additional biomarker of interest~~.

5 ….

AE = adverse event, CT = computed tomography, CTC = Common Toxicity Criteria, DCE-MRI = Dynamic Contrast-Enhanced Magnetic Resonance Imaging, ECG = electrocardiogram, ECOG = Eastern Cooperative Oncology Group, MRI = Magnetic Resonance Imaging, NCI = National Cancer Institute, PK = pharmacokinetics, PlGF = placental growth factor, RECIST = Response Evaluation Criteria In Solid Tumors.

New text:

Page 11:

…						
Blood samples for PK **and VEGF-A**		x[5]	x	x	x	x
…						

Please see next page for footnotes

Page 12:

…						
Blood samples for PK **and VEGF-A**	x	x	x	x	x	
…						

At Days 1, 22, 29, 36, and 43 all assessments will be performed pre-dose if not otherwise noted. AEs/toxicity will be recorded pre- and post-dose.
[1] To be performed within 28 days prior to Day 1.
[2] Taken during 30 days prior to study start.
[3] Pulse rate and blood pressure will be assessed pre-dose and 30 min, 1 h, 2 h, 4 h and 8 h post-dose.
[4] Biomarkers: **potential cancer-associated biomarkers in serum using the Human OncologyMAP™**.
5 ….

AE = adverse event, CT = computed tomography, CTC = Common Toxicity Criteria, DCE-MRI = Dynamic Contrast-Enhanced Magnetic Resonance Imaging, ECG = electrocardiogram, ECOG = Eastern Cooperative Oncology Group, MRI = Magnetic Resonance Imaging, NCI = National Cancer Institute, PK = pharmacokinetics, PlGF = placental growth factor, RECIST = Response Evaluation Criteria In Solid Tumors**, VEGF-A = Vascular Endothelial Growth Factor**.

2.3 Section 11.4, 11.5.1, and 11.5.2 (Change 1)

Original text:

- …
- Blood samples for determination of ~~PlGF in plasma and one additional biomarker of interest~~ in serum;
- …

New text:

- …
- Blood samples for determination of **potential cancer-associated biomarkers** in serum;
- …

2.4 Section 11.6 (Change 1)

Original text:

- …
- Blood samples for determination of ~~PlGF and one additional biomarker of interest~~ in serum (only on Day 43).

…

New text:

- …
- Blood samples for determination of **potential cancer-associated biomarkers** in serum (only on Day 43).

…

2.5 Section 14.3 (Change 1 and 2)

Original text:

Blood samples for the analysis of ~~PlGF in plasma and one additional biomarker of interest~~ in serum will be taken at Day 1 (pre-dose) and Days 2, 3, 5 (optional), 15 and 43. Detailed written instructions regarding collection and storage of samples will be given to the investigator~~. Biomarker levels will be analyzed using a commercially available immunoassay.~~

New text:

Blood samples for the analysis of **potential cancer-associated biomarkers** in serum will be taken at Day 1 (pre-dose) and Days 2, 3, 5 (optional), 15 and 43. Detailed written instructions regarding collection and storage of samples will be given to the investigator. **Samples will be analyzed using the Human OncologyMAP™ v 1.0 (Rules-Based Medicine, Inc.)**.

VEGF-A (free VEGF-A and VEGF-A in complex with Angiocal®, if applicable) will be analyzed at Pieris AG. The analysis will be performed on PK samples, when these are no longer required for PK measurements (see Section 13.1).

2.6 Section 15.6.1 (Change 1)

Original text:

"~~Plasma PlGF levels and one additional biomarker of interest in serum~~ will be analyzed and reported separately."

New text:

"**Cancer-associated** b**iomarker levels in serum** will be analyzed and reported separately."

2.7 Section 13.1 (Change 2)

Original text:

Pharmacokinetic samples will be shipped to and analyzed by Covance Ltd., Harrogate, United Kingdom. Detailed written instructions regarding collection and storage of blood samples will be given to the investigator. PK data analysis will be done at Covance Clinical Research Unit, Leeds, United Kingdom.

New text:

Pharmacokinetic samples will be shipped to and analyzed by Covance Ltd., Harrogate, United Kingdom. Detailed written instructions regarding collection and storage of blood samples will be given to the investigator. PK data analysis will be done at Covance Clinical Research Unit, Leeds, United Kingdom.

When PK aliquots are no longer required for PK measurements, PK samples will be used for the determination of VEGF-A (see Section 14.3).

2.8 Section 1 (Change 3)

Original text:

…						
AEs/toxicity (NCI-CTCAE v3.0)	x	x	x	x	x	x

…
[2] ….
[3] Pulse rate ~~and~~ blood pressure will be assessed pre-dose and 30 min, 1 h, 2 h, 4 h and 8 h post-dose.
[4] ….

New text:

…						
AEs/toxicity (NCI-CTCAE v3.0)	x	x	x	x	x	x

…
[2] ….
[3] Pulse rate**,** blood pressure **and temperature** will be assessed pre-dose and 30 min, 1 h, 2 h, 4 h and 8 h post-dose.
[4] ….

2.9 Section 11.4 and 11.6 (Change 3)

Original text:

- …
- Vital signs: pulse rate ~~and~~ blood pressure 30 min, 1 h, 2 h, 4 h and 8 h post-dose (If

the patient agrees, the investigator may decide to perform a 24-hour blood pressure measurement. This may be reasonable in patients assigned to doses of Angiocal® of 6 mg/kg or higher);

- …

New text:

- …
- Vital signs: pulse rate, blood pressure **and temperature** 30 min, 1 h, 2 h, 4 h and 8 h post-dose (If the patient agrees, the investigator may decide to perform a 24-hour blood pressure measurement. This may be reasonable in patients assigned to doses of Angiocal® of 6 mg/kg or higher);
- …

2.10 Section 12.5 (Change 3)

Original text:

Vital signs (heart rate, blood pressure and body temperature) will be measured at each visit. Systolic and diastolic blood pressure as well as pulse rate will be measured in sitting position after at least 5 minutes at rest. At Baseline, Days 22, 29, 36, and 43 pulse rate ~~and~~ blood pressure will be assessed pre-dose and 30 min, 1 h, 2 h, 4 h, and 8 h post-dose. If the patient agrees, the investigator may decide to perform a 24-hour blood pressure measurement. This may be reasonable in patients assigned to doses of Angiocal® of 6 mg/kg or higher.

New text:

Vital signs (heart rate, blood pressure and body temperature) will be measured at each visit. Systolic and diastolic blood pressure as well as pulse rate will be measured in sitting position after at least 5 minutes at rest. At Baseline, Days 22, 29, 36, and 43 pulse rate, blood pressure **and temperature** will be assessed pre-dose and 30 min, 1 h, 2 h, 4 h, and 8 h post-dose. If the patient agrees, the investigator may decide to perform a 24-hour blood pressure measurement. This may be reasonable in patients assigned to doses of Angiocal® of 6 mg/kg or higher.

3 APPROVAL OF AMENDMENT

Version: Final 1.0

Date: 15-Nov-2010

Study code: Pieris001

Sponsor: Pieris AG, Freising, Germany

The signatories declare, that they have read the protocol Amendment and that they agree with its contents.

Sponsor's signatory

Dr. Angelika C. Stern
Head of Project Management Angiogenesis
Pieris AG
Lise-Meitner-Straße 30
85354 Freising
Germany

15.11.2010 — Date

[signature] — Signature

Coordinating Investigator and Director Clinical Investigation (LKP)

PD Dr. med. Klaus Mross
Klinik für Tumorbiologie an der Albert-Ludwigs-Universität Freiburg
Department of Medical Oncology
Breisacher Str. 117
76106 Freiburg im Breisgau, Germany

Date

Signature

3 APPROVAL OF AMENDMENT

Version: Final 1.0

Date: 15-Nov-2010

Study code: Pieris001

Sponsor: Pieris AG, Freising, Germany

The signatories declare, that they have read the protocol Amendment and that they agree with its contents.

Sponsor's signatory

Dr. Angelika C. Stern
Head of Project Management Angiogenesis
Pieris AG
Lise-Meitner-Straße 30
85354 Freising
Germany

__________ Date

__________ Signature

Coordinating Investigator and Director Clinical Investigation (LKP)

PD Dr. med. Klaus Mross
Klinik für Tumorbiologie an der Albert-Ludwigs-Universität Freiburg
Department of Medical Oncology
Breisacher Str. 117
76106 Freiburg im Breisgau, Germany

15.11.10
Date

[signature]
Signature

CRO's signatory

Dr. med. Edgar J. Fenzl
Managing Director/Medical Monitor
FGK Clinical Research GmbH
Heimeranstrasse 35
80339 München, Germany

18.11.2010 — Date
[signature] — Signature

Dr. Monika Schwienbacher
Associate Project Director
FGK Clinical Research GmbH
Heimeranstrasse 35
80339 München, Germany

15.11.2010 — Date
[signature] — Signature

Principal Investigator

Investigator's name (printed)

Date

Signature

Amendment 5

Amending protocol Version 2.0 of 10-May-2010

A PHASE I, OPEN-LABEL STUDY OF THE SAFETY, TOLERABILITY, AND PHARMACOKINETICS OF ANGIOCAL® (PRS-050-PEG40) IN PATIENTS WITH SOLID TUMORS

Protocol code: Pieris001

EudraCT No.: 2009-015601-38

**Previous amendments:**	**Amendment 1 of 11-Jun-2010**<br>**Amendment 2 of 22-Jun-2010**<br>**Amendment 3 of 10-Sep-2010**<br>**Amendment 4 of 15-Nov-2010**
**Sponsor:**	**Pieris AG**<br>**Lise-Meitner-Straße 30**<br>**85354 Freising**<br>**Germany**
**Amendment Version:**	**Final 1.0**
**Amendment Date:**	**16-Feb-2011**

TABLE OF CONTENTS

Page

1 OVERVIEW OF CHANGES 3
2 PROTOCOL SECTIONS CHANGED 3
2.1 Section 1 3
2.2 Section 8.1 5
2.3 Section 8.2.2 5
2.4 Section 11.7 6
2.5 Section 11.9 6
3 APPROVAL OF AMENDMENT 7

1 OVERVIEW OF CHANGES

The following change to the clinical study protocol Version 2.0 is made:

The continuation of treatment despite the DLT event fever, rigors/chills, or acute infusion reaction will be allowed, provided that this DLT event is not life threatening, that there is evidence that this DLT event can be treated, and that the patient was made fully aware of the risks and benefits of further treatment with the study medication. If a patient continues with treatment despite such a DLT event, prophylactic treatment with clemastin, ranitidine, and fortecortin to prevent the reoccurrence of this event is required before the administration of each following dose.

2 PROTOCOL SECTIONS CHANGED

The protocol sections that are changed are detailed below. Bolded text in the "new text" is used to indicate the addition of information to the original text.

2.1 Section 1

Original text:

…..In the absence of any dose limiting toxicity (DLT) and based on the patient's and investigator's decision, patients will continue into the repeated dosing period, where they will be treated and evaluated once weekly for 4 weeks on Days 22, 29, 36, and 43[1]. A Final Visit will be performed on Day 71…..

[1] At Day 43, patients who respond to Angiocal® treatment or have stable disease will, at the discretion of the investigator, be given the opportunity to get further treatment with Angiocal® in a biweekly schedule. The Final Visit at Day 71 will not be performed in these patients.

New text:

…..In the absence of any dose limiting toxicity (DLT) and based on the patient's and investigator's decision, patients will continue into the repeated dosing period, where they will be treated and evaluated once weekly for 4 weeks on Days 22, 29, 36, and 43[1]. **If a patient experiences the DLT event fever, rigors/chills, or acute infusion reaction, and this DLT event is not considered to be life threatening and responds to treatment, the patient may continue to be treated based on the patient's and investigator's decision. Prophylactic treatment with clemastin, ranitidine, and fortecortin is then required before the administration of each following dose.** A Final Visit will be performed on Day 71…..

[1] At Day 43, patients who respond to Angiocal® treatment or have stable disease will, at the discretion of the investigator, be given the opportunity to get further treatment with Angiocal® in a biweekly schedule. The Final Visit at Day 71 will not be performed in these patients.

Original text:

Those events will be indicated as DLT if they occur after the single dose application within the follow-up period (until Day 15). Patients who experience a DLT after the single dose application within the

follow-up period will not continue into the repeated dosing period. Patients who experience one of those events during the repeated dosing period will be withdrawn from the study.

New text:

Those events will be indicated as DLT if they occur after the single dose application within the follow-up period (until Day 15). Patients who experience a DLT after the single dose application within the follow-up period will not continue into the repeated dosing period[*]. Patients who experience one of those events during the repeated dosing period will be withdrawn from the study[*].

[*] **If within the single dose or the repeated dosing period, a patient experiences the DLT event fever, rigors/chills, or actue infusion reaction, and this DLT event is not considered to be life threatening and responds to treatment, a patient may continue to be treated based on the patient's and investigator's decision. Prophylactic treatment with clemastin, ranitidine, and fortecortin is then required before the administration of each following dose.**

Original text:

Each patient will come to the study site according to the following schedule:

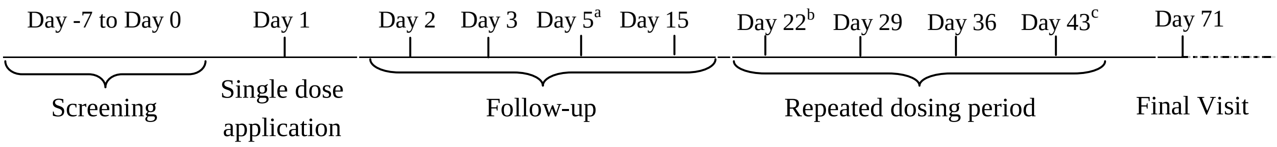

[a] Visit on Day 5 is optional and is at the discretion of the investigator.
[b] In the absence of any DLT and based on the patient's and investigator's decision, patients will continue into the repeated dosing period.
[c] Patients who respond to Angiocal® treatment or have stable disease will, at the discretion of the investigator, be given the opportunity to get further treatment with Angiocal® in a biweekly schedule. The Final Visit at Day 71 will not be performed in these patients.

New text:

Each patient will come to the study site according to the following schedule:

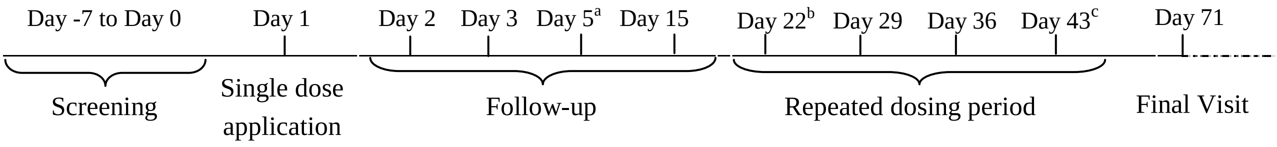

[a] Visit on Day 5 is optional and is at the discretion of the investigator.
[b] In the absence of any DLT and based on the patient's and investigator's decision, patients will continue into the repeated dosing period. **If a patient experiences the DLT event fever, rigors/chills, or acute infusion reaction, and this DLT event is not considered to be life threatening and responds to treatment a patient may continue to be treated based on the patient's and investigator's decision. Prophylactic treatment with clemastin, ranitidine, and fortecortin is then required before the administration of each following dose.**
[c] Patients who respond to Angiocal® treatment or have stable disease will, at the discretion of the investigator, be given the opportunity to get further treatment with Angiocal® in a biweekly schedule. The Final Visit at Day 71 will not be performed in these patients.

2.2 Section 8.1

Original text:

….In the absence of any dose limiting toxicity (DLT) and based on the patient's and investigator's decision, patients will continue with the repeated dosing period, where they will be treated and evaluated once weekly for 4 weeks on Days 22, 29, 36, and 43. A Final Visit will be performed on Day 71….

New text:

....In the absence of any dose limiting toxicity (DLT) and based on the patient's and investigator's decision, patients will continue with the repeated dosing period, where they will be treated and evaluated once weekly for 4 weeks on Days 22, 29, 36, and 43. **If a patient experiences the DLT event fever, rigors/chills, or acute infusion reaction, and this DLT event is not considered to be life threatening and responds to treatment, the patient may continue to be treated based on the patient's and investigator's decision. Prophylactic treatment with clemastin, ranitidine, and fortecortin is then required before the administration of each following dose.** A Final Visit will be performed on Day 71....

2.3 Section 8.2.2

Original text:

Patients who experience a DLT after the single dose application within the follow-up period will not continue into the repeated dosing period. Patients who experience a DLT event during the repeated dosing period will be withdrawn from the study.

New text:

Patients who experience a DLT after the single dose application within the follow-up period will not continue into the repeated dosing period[*]. Patients who experience a DLT event during the repeated dosing period will be withdrawn from the study[*].

[*] **If within the single dose or the repeated dosing period, a patient experiences the DLT event fever, rigors/chills, or acute infusion reaction, and this DLT event is not considered to be life threatening and responds to treatment, a patient may continue to be treated based on the patient's and investigator's decision. Prophylactic treatment with clemastin, ranitidine, and fortecortin is then required before the administration of each following dose.**

2.4 Section 11.7

Original text:

In the absence of any DLT and in case of stable disease or response to treatment at Day 43 the investigator may decide to continue with Angiocal® treatment if the patient agrees. Angiocal® will be given biweekly (every 2 weeks) starting at Day 57. The investigator will decide to continue treatment during the follow-up visits every two weeks….

New text:

In the absence of any DLT and in case of stable disease or response to treatment at Day 43 the investigator may decide to continue with Angiocal® treatment if the patient agrees. **If a patient experiences the DLT event fever, rigors/chills, or acute infusion reaction, and this DLT event is not considered to be life threatening and responds to treatment, a patient may continue to be treated based on the patient's and investigator's decision. Prophylactic treatment with clemastin, ranitidine, and fortecortin is then required before the administration of each following dose.** Angiocal® will be given biweekly (every 2 weeks) starting at Day 57. The investigator will decide to continue treatment during the follow-up visits every two weeks….

2.5 Section 11.9

Original text:

In the absence of any DLT and in case of stable disease or response to treatment at Day 43 the investigator may decide to continue with Angiocal® treatment if the patient agrees. Angiocal® will be given biweekly starting at Day 57. The Final Visit at Day 71 will not be performed in these patients.

New text:

In the absence of any DLT and in case of stable disease or response to treatment at Day 43 the investigator may decide to continue with Angiocal® treatment if the patient agrees. **If a patient experiences the DLT event fever, rigors/chills, or acute infusion reaction, and this DLT event is not considered to be life threatening and responds to treatment, a patient may continue to be treated based on the patient's and investigator's decision. Prophylactic treatment with clemastin, ranitidine, and fortecortin is then required before the administration of each following dose.** Angiocal® will be given biweekly starting at Day 57. The Final Visit at Day 71 will not be performed in these patients.

3 APPROVAL OF AMENDMENT

Version: Final 1.0

Date: 16-Feb-2011

Study code: Pieris001

Sponsor: Pieris AG, Freising, Germany

The signatories declare, that they have read the protocol Amendment and that they agree with its contents.

Sponsor's signatory

Dr. Angelika C. Stern
Head of Project Management Angiogenesis
Pieris AG
Lise-Meitner-Straße 30
85354 Freising
Germany

February 16, 2011 — Date

[signature] — Signature

Coordinating Investigator and Director Clinical Investigation (LKP)

PD Dr. med. Klaus Mross
Klinik für Tumorbiologie an der Albert-Ludwigs-Universität Freiburg
Department of Medical Oncology
Breisacher Str. 117
76106 Freiburg im Breisgau, Germany

Date

Signature

3 APPROVAL OF AMENDMENT

Version: Final 1.0

Date: 16-Feb-2011

Study code: Pieris001

Sponsor: Pieris AG, Freising, Germany

The signatories declare, that they have read the protocol Amendment and that they agree with its contents.

Sponsor's signatory

Dr. Angelika C. Stern
Head of Project Management Angiogenesis
Pieris AG
Lise-Meitner-Straße 30
85354 Freising
Germany

Date

Signature

Coordinating Investigator and Director Clinical Investigation (LKP)

PD Dr. med. Klaus Mross
Klinik für Tumorbiologie an der Albert-Ludwigs-Universität Freiburg
Department of Medical Oncology
Breisacher Str. 117
76106 Freiburg im Breisgau, Germany

16.2.11
Date

Signature

CRO's signatory

Dr. med. Edgar J. Fenzl
Managing Director/Medical Monitor
FGK Clinical Research GmbH
Heimeranstrasse 35
80339 München, Germany

16.02.2011 — Date
Signature

Dr. Monika Schwienbacher
Associate Project Director
FGK Clinical Research GmbH
Heimeranstrasse 35
80339 München, Germany

16.02.2011 — Date
Signature

Principal Investigator

Investigator's name (printed) | Date | Signature

A PHASE I, OPEN-LABEL STUDY OF THE SAFETY, TOLERABILITY, AND PHARMACOKINETICS OF ANGIOCAL® (PRS-050-PEG40) IN PATIENTS WITH SOLID TUMORS

Amendment 6

Amending protocol Version 2.0 of 10-May-2010

Including previous amendments: **Amendment 1 of 11-Jun-2010**
Amendment 2 of 22-Jun-2010
Amendment 3 of 10-Sep-2010
Amendment 4 of 15-Nov-2010
Amendment 5 of 16-Feb-2011

Protocol code: **Pieris001**
EudraCT No.: **2009-015601-38**

Sponsor: **Pieris AG**
Lise-Meitner-Straße 30
85354 Freising
Germany

Amendment Version: **Final 1.0**
Amendment Date: **28-Feb-2011**

TABLE OF CONTENTS

Page

1 OVERVIEW OF CHANGES 3

2 PROTOCOL SECTIONS CHANGED 4

2.1 Section 1 (Change 1) 4

2.2 Section 8.1 (Change 1) 5

2.3 Section 8.2.2 (Change 1) 6

2.4 Section 10.1.3 (Changes 1 and 3) 6

2.5 Section 10.2 (Changes 1 and 4) 7

2.6 Section 11.4 (Changes 1 and 2) 8

2.7 Section 11.6 (Changes 1 and 2) 8

2.8 Section 11.7 (Change 1) 9

2.9 Section 11.9 (Change 1) 10

2.10 Section 14.3 (Change 2) 10

3 APPROVAL OF AMENDMENT 12

1 OVERVIEW OF CHANGES

The following changes to the clinical study protocol Version 2.0 are made:

Change 1:

Prophylactic treatment with intravenously administered clemastin, ranitidine, and fortecortin before the administration of each dose of Angiocal® is included.

Justification:

Since a dose-dependent increase in the frequency and intensity of infusion reactions including fever and chills were observed in this study, the administration of prophylactic treatment with clemastin, ranitidine, and fortecortin prior to infusions of Angiocal® is included. It is anticipated that pre-medication will prevent these symptoms.

Change 2:

The analysis of inflammation markers is included. This analysis will be performed using existing PK samples. Moreover, the collection of additional blood samples for the analysis of hematology, clinical chemistry, and coagulation is included one hour after each administration of Angiocal® during the single and repeated dosing period.

Justification:

In order to collect as much information as possible on the mechanisms underlying the above mentioned infusion reactions, inflammation markers and clinical laboratory parameters will be analyzed.

Change 3:

The duration of the bolus injection of Angiocal® is extended from 3 to 5 minutes to approximately 20 minutes.

Justification:

In the current cohorts, patients receive larger volumes of Angiocal® and the time of administration is therefore changed to up to approximately 20 minutes.

Change 4:

A clarification on concomitant medication will be included.

Justification:

Patients with chronic daily treatment with aspirin (>325 mg/day), clopidogrel (>75 mg/day), or corticosteroids (dose of ≥10 mg/day methylprednisolone or equivalent) were excluded from the study by exclusion criteria #3 and #4. Prohibited medications were listed within the main part of the protocol (Section 10.2) without giving the maximum allowed doses of these medications. The corresponding section of the protocol will be changed accordingly.

2 PROTOCOL SECTIONS CHANGED

The protocol sections that are changed are detailed below. Bold text in the "new text" is used to indicate the addition, crossed out text in the "original text" indicates the deletion of information from the original text. Original text included previous amendments if applicable.

2.1 Section 1 (Change 1)

Original text:

….The single dose application will be followed by a 3-week follow-up period prior to start of the repeated dosing period. The time between the single dose and the repeated dosing period may be adjusted should the plasma half-life of the drug differ from the predicted duration based on pre-clinical studies. Patients will be evaluated at Screening (Day -7 to 0) and Baseline (Day 1)…….~~If a patient experiences the DLT event fever, rigors/chills, or acute infusion reaction, and this DLT event is not considered to be life threatening and responds to treatment, the patient may continue to be treated based on the patient's and investigator's decision. Prophylactic treatment with clemastin, ranitidine, and fortecortin is then required before the administration of each following dose.~~ A Final Visit will be performed on Day 71…..

New text:

….The single dose application will be followed by a 3-week follow-up period prior to start of the repeated dosing period. The time between the single dose and the repeated dosing period may be adjusted should the plasma half-life of the drug differ from the predicted duration based on pre-clinical studies. **Prior to each administration of Angiocal®, prophylactic treatment will be administered.** Patients will be evaluated at Screening (Day -7 to 0) and Baseline (Day 1)…….A Final Visit will be performed on Day 71…..

Original text:

Those events will be indicated as DLT if they occur after the single dose application within the follow-up period (until Day 15). Patients who experience a DLT after the single dose application within the follow-up period will not continue into the repeated dosing period*. Patients who experience one of those events during the repeated dosing period will be withdrawn from the study*.

~~* If within the single dose or the repeated dosing period, a patient experiences the DLT event fever, rigors/chills, or actue infusion reaction, and this DLT event is not considered to be life threatening and responds to treatment, a patient may continue to be treated based on the patient's and investigator's decision. Prophylactic treatment with clemastin, ranitidine, and fortecortin is then required before the administration of each following dose.~~

New text:

Those events will be indicated as DLT if they occur after the single dose application within the follow-up period (until Day 15). Patients who experience a DLT after the single dose application within the

follow-up period will not continue into the repeated dosing period. Patients who experience one of those events during the repeated dosing period will be withdrawn from the study.

Original text:

Each patient will come to the study site according to the following schedule:

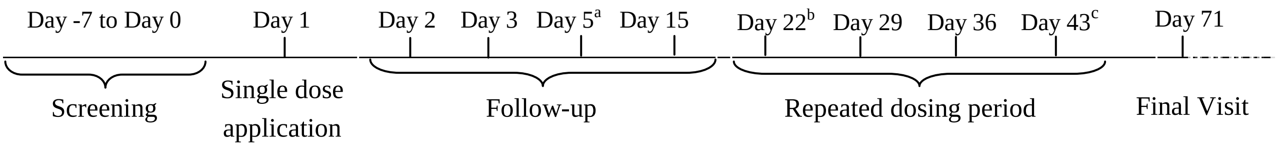

[a] Visit on Day 5 is optional and is at the discretion of the investigator.
[b] In the absence of any DLT and based on the patient’s and investigator's decision, patients will continue into the repeated dosing period. ~~If a patient experiences the DLT event fever, rigors/chills, or acute infusion reaction, and this DLT event is not considered to be life threatening and responds to treatment a patient may continue to be treated based on the patient’s and investigator's decision. Prophylactic treatment with clemastin, ranitidine, and fortecortin is then required before the administration of each following dose.~~
[c] Patients who respond to Angiocal® treatment or have stable disease will, at the discretion of the investigator, be given the opportunity to get further treatment with Angiocal® in a biweekly schedule. The Final Visit at Day 71 will not be performed in these patients.

New text:

Each patient will come to the study site according to the following schedule:

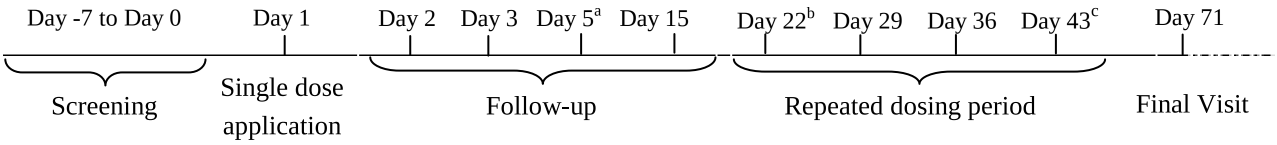

[a] Visit on Day 5 is optional and is at the discretion of the investigator.
[b] In the absence of any DLT and based on the patient’s and investigator's decision, patients will continue into the repeated dosing period.
[c] Patients who respond to Angiocal® treatment or have stable disease will, at the discretion of the investigator, be given the opportunity to get further treatment with Angiocal® in a biweekly schedule. The Final Visit at Day 71 will not be performed in these patients.

2.2 Section 8.1 (Change 1)

Original text:

….The single dose application will be followed by a 3-week follow-up period prior to start of the repeated dosing period. The time between the single dose and the repeated dosing period may be adjusted should the plasma half-life of the drug differ from the predicted duration based on pre-clinical studies. Patients will be evaluated at Screening (Day -7 to 0) and Baseline (Day 1)….~~If a patient experiences the DLT event fever, rigors/chills, or acute infusion reaction, and this DLT event is not considered to be life threatening and responds to treatment, the patient may continue to be treated~~

~~based on the patient's and investigator's decision. Prophylactic treatment with clemastin, ranitidine, and fortecortin is then required before the administration of each following dose.~~ A Final Visit will be performed on Day 71....

New text:

….The single dose application will be followed by a 3-week follow-up period prior to start of the repeated dosing period. The time between the single dose and the repeated dosing period may be adjusted should the plasma half-life of the drug differ from the predicted duration based on pre-clinical studies. **Prior to each administration of Angiocal®, prophylactic treatment will be administered.** Patients will be evaluated at Screening (Day -7 to 0) and Baseline (Day 1)…. A Final Visit will be performed on Day 71....

2.3 Section 8.2.2 (Change 1)

Original text:

Patients who experience a DLT after the single dose application within the follow-up period will not continue into the repeated dosing period*. Patients who experience a DLT event during the repeated dosing period will be withdrawn from the study*.

~~* If within the single dose or the repeated dosing period, a patient experiences the DLT event fever, rigors/chills, or acute infusion reaction, and this DLT event is not considered to be life threatening and responds to treatment, a patient may continue to be treated based on the patient's and investigator's decision. Prophylactic treatment with clemastin, ranitidine, and fortecortin is then required before the administration of each following dose.~~

New text:

Patients who experience a DLT after the single dose application within the follow-up period will not continue into the repeated dosing period. Patients who experience a DLT event during the repeated dosing period will be withdrawn from the study.

2.4 Section 10.1.3 (Changes 1 and 3)

Original text:

Angiocal® is to be administered intravenously by a slow bolus injection preferably over~~ 3 to 5 minutes~~. The volume to be administered to a patient is calculated according to handling instructions. Patients will receive 5 i.v. injections, one single treatment and 4 treatments during the repeated dosing period. During the repeated dosing period study medication will be administered once weekly for 4 weeks on Days 22, 29, 36, and 43. After each administration of Angiocal® 10 mL of a 0.9% sodium chloride solution will be administered intravenously.

New text:

90 minutes (±15 minutes) before each administration of Angiocal®, prophylactic treatment with clemastin (2 mg), ranitidine (50 mg), and fortecortin (16 mg) will be administered intravenously, starting with the third patient of the sixth cohort.

Angiocal® is to be administered intravenously by a slow bolus injection preferably over **up to approximately 20 minutes**. The volume to be administered to a patient is calculated according to handling instructions. Patients will receive 5 i.v. injections, one single treatment and 4 treatments during the repeated dosing period. During the repeated dosing period study medication will be administered once weekly for 4 weeks on Days 22, 29, 36, and 43. After each administration of Angiocal® 10 mL of a 0.9% sodium chloride solution will be administered intravenously.

2.5 Section 10.2 (Changes 1 and 4)

Original text:

All medication taken during 30 days prior to study start as well as all concurrent medication taken by the patient during the course of the study will be documented in the corresponding section of the electronic case report form (eCRF) along with the indication and duration of treatment. Medication use will be reviewed by the investigator and checked against the exclusion criteria.

The use of any of the following medications is prohibited during the course of the study:

- Anticancer therapy;
- Aspirin;
- Clopidogrel;
- Corticosteroids.

New text:

90 minutes (±15 minutes) before each administration of Angiocal®, prophylactic treatment with clemastin (2 mg), ranitidine (50 mg), and fortecortin (16 mg) will be administered intravenously, starting with the third patient of the sixth cohort.

All medication taken during 30 days prior to study start as well as all concurrent medication taken by the patient during the course of the study will be documented in the corresponding section of the electronic case report form (eCRF) along with the indication and duration of treatment. Medication use will be reviewed by the investigator and checked against the exclusion criteria.

The use of any of the following medications is prohibited during the course of the study:

- Anticancer therapy;
- Aspirin **(>325 mg/day)**;
- Clopidogrel **(>75 mg/day)**;

- Corticosteroids **(dose of ≥10 mg/day methylprednisolone or equivalent), with the exception of inhaled steroids and fortecortin**.

2.6 Section 11.4 (Changes 1 and 2)

Original text:

The following assessments will be performed before the first i.v. injection of Angiocal®:

- …
- AEs and toxicity (National Cancer Institute Common Toxicity Criteria for AEs [NCI-CTCAE] version 3.0);

…

The following assessments will take place after the first i.v. injection of Angiocal®:

- …
- AEs and toxicity (NCI-CTCAE version 3.0)~~.~~

New text:

The following assessments will be performed before the first i.v. injection of Angiocal®:

- …
- AEs and toxicity (National Cancer Institute Common Toxicity Criteria for AEs [NCI-CTCAE] version 3.0);
- **Intravenous administration of 2 mg clemastin, 50 mg ranitidine, and 16 mg fortecortin 90 min (±15 min) before Angiocal® injection**

…

The following assessments will take place after the first i.v. injection of Angiocal®:

- …
- AEs and toxicity (NCI-CTCAE version 3.0)**;**
- **Blood sampling for the analysis of hematology, clinical chemistry, and coagulation (1 hour ±5 min after Angiocal® injection)**

2.7 Section 11.6 (Changes 1 and 2)

Original text:

The following assessments will take place before the first i.v. injection of Angiocal® during the repeated dosing period:

- …

- Blood samples for determination of potential cancer-associated biomarkers in serum (only on Day 43)~~.~~

…

<u>Note:</u> An emergency kit for the treatment of a potential anaphylactic shock should be readily available. The following assessments will take place after the first i.v. injection of Angiocal® during the repeated dosing period:

- …
- AEs and toxicity (NCI-CTCAE version 3.0)~~.~~

<u>New text:</u>

The following assessments will take place before the first i.v. injection of Angiocal® during the repeated dosing period:

- …
- Blood samples for determination of potential cancer-associated biomarkers in serum (only on Day 43)**;**
- **Intravenous administration of 2 mg clemastin, 50 mg ranitidine, and 16 mg fortecortin 90 min (±15 min) before Angiocal® injection.**

…

<u>Note:</u> An emergency kit for the treatment of a potential anaphylactic shock should be readily available. The following assessments will take place after the first i.v. injection of Angiocal® during the repeated dosing period:

- …
- AEs and toxicity (NCI-CTCAE version 3.0)**;**
- **Blood sampling for the analysis of hematology, clinical chemistry, and coagulation (1 hour ±5 min after Angiocal® injection)**

2.8 <u>Section 11.7 (Change 1)</u>

<u>Original text:</u>

In the absence of any DLT and in case of stable disease or response to treatment at Day 43 the investigator may decide to continue with Angiocal® treatment if the patient agrees. ~~If a patient experiences the DLT event fever, rigors/chills, or acute infusion reaction, and this DLT event is not considered to be life threatening and responds to treatment, a patient may continue to be treated based on the patient's and investigator's decision. Prophylactic treatment with clemastin, ranitidine, and fortecortin is then required before the administration of each following dose.~~ Angiocal® will be given

biweekly (every 2 weeks) starting at Day 57. The investigator will decide to continue treatment during the follow-up visits every two weeks….

New text:

In the absence of any DLT and in case of stable disease or response to treatment at Day 43 the investigator may decide to continue with Angiocal® treatment if the patient agrees. **90 minutes (±15 min) before each administration of Angiocal®, prophylactic treatment with clemastin (2 mg), ranitidine (50 mg), and fortecortin (16 mg) will be administered.** Angiocal® will be given biweekly (every 2 weeks) starting at Day 57. The investigator will decide to continue treatment during the follow-up visits every two weeks….

2.9 Section 11.9 (Change 1)

Original text:

In the absence of any DLT and in case of stable disease or response to treatment at Day 43 the investigator may decide to continue with Angiocal® treatment if the patient agrees. ~~If a patient experiences the DLT event fever, rigors/chills, or acute infusion reaction, and this DLT event is not considered to be life threatening and responds to treatment, a patient may continue to be treated based on the patient’s and investigator's decision. Prophylactic treatment with clemastin, ranitidine, and fortecortin is then required before the administration of each following dose.~~ Angiocal® will be given biweekly starting at Day 57. The Final Visit at Day 71 will not be performed in these patients.

New text:

In the absence of any DLT and in case of stable disease or response to treatment at Day 43 the investigator may decide to continue with Angiocal® treatment if the patient agrees. Angiocal® will be given biweekly starting at Day 57. The Final Visit at Day 71 will not be performed in these patients.

2.10 Section 14.3 (Change 2)

Original text:

14.3 Assessment of biomarkers

Blood samples for the analysis of potential cancer-associated biomarkers in serum will be taken at Day 1 (pre-dose) and Days 2, 3, 5 (optional), 15 and 43. Detailed written instructions regarding collection and storage of samples will be given to the investigator. Samples will be analyzed using the Human OncologyMAP™ v 1.0 (Rules-Based Medicine, Inc.).

VEGF-A (free VEGF-A and VEGF-A in complex with Angiocal®, if applicable) will be analyzed at Pieris AG. The analysis will be performed on PK samples, when these are no longer required for PK measurements (see Section 13.1).

New text:

14.3 Assessment of biomarkers **and markers of inflammation**

Blood samples for the analysis of potential cancer-associated biomarkers in serum will be taken at Day 1 (pre-dose) and Days 2, 3, 5 (optional), 15 and 43. Detailed written instructions regarding collection and storage of samples will be given to the investigator. Samples will be analyzed using the Human OncologyMAP™ v 1.0 (Rules-Based Medicine, Inc.).

VEGF-A (free VEGF-A and VEGF-A in complex with Angiocal®, if applicable) will be analyzed at Pieris AG. The analysis **of VEGF-A and inflammation markers** will be performed on PK samples, when these are no longer required for PK measurements (see Section 13.1).

3 APPROVAL OF AMENDMENT

Version: Final 1.0

Date: 28-Feb-2011

Study code: Pieris001

Sponsor: Pieris AG, Freising, Germany

The signatories declare, that they have read the protocol Amendment and that they agree with its contents.

Sponsor's signatory

Dr. Angelika C. Stern
Head of Project Management Angiogenesis
Pieris AG
Lise-Meitner-Straße 30
85354 Freising
Germany

February 28, 2011
Date

Signature

Coordinating Investigator and Director Clinical Investigation (LKP)

PD Dr. med. Klaus Mross
Klinik für Tumorbiologie an der Albert-Ludwigs-Universität Freiburg
Department of Medical Oncology
Breisacher Str. 117
76106 Freiburg im Breisgau, Germany

Date

Signature

3 APPROVAL OF AMENDMENT

Version: Final 1.0

Date: 28-Feb-2011

Study code: Pieris001

Sponsor: Pieris AG, Freising, Germany

The signatories declare, that they have read the protocol Amendment and that they agree with its contents.

Sponsor's signatory

Dr. Angelika C. Stern
Head of Project Management Angiogenesis
Pieris AG
Lise-Meitner-Straße 30
85354 Freising
Germany

Date ______ Signature ______

Coordinating Investigator and Director Clinical Investigation (LKP)

PD Dr. med. Klaus Mross
Klinik für Tumorbiologie an der Albert-Ludwigs-Universität Freiburg
Department of Medical Oncology
Breisacher Str. 117
76106 Freiburg im Breisgau, Germany

1.3.11
Date

Signature

KTB Klinik für Tumorbiologie
Priv.-Doz. Dr. med. Klaus Mross
Geschäftsführender Oberarzt
Klinik für Internistische Onkologie
Breisacherstraße 117 · D - 79106 Freiburg
Fon: 07 61/206-18 33 · Fax: 07 61 /206-18 32

CRO's signatory

Dr. med. Edgar J. Fenzl
Managing Director/Medical Monitor
FGK Clinical Research GmbH
Heimeranstrasse 35
80339 München, Germany

18.03.2011 — Date
Signature

Dr. Monika Schwienbacher
Associate Project Director
FGK Clinical Research GmbH
Heimeranstrasse 35
80339 München, Germany

28/02/2011 — Date
Signature

Principal Investigator

Investigator's name (printed) — Date — Signature

A PHASE I, OPEN-LABEL STUDY OF THE SAFETY, TOLERABILITY, AND PHARMACOKINETICS OF ANGIOCAL® (PRS-050-PEG40) IN PATIENTS WITH SOLID TUMORS

Amendment 7

Amending protocol Version 2.0 of 10-May-2010

**Including previous amendments:**	**Amendment 1 of 11-Jun-2010**
	**Amendment 2 of 22-Jun-2010**
	**Amendment 3 of 10-Sep-2010**
	**Amendment 4 of 15-Nov-2010**
	**Amendment 5 of 16-Feb-2011**
	**Amendment 6 of 28-Feb-2011**

**Protocol code:**	**Pieris001**
**EudraCT No.:**	**2009-015601-38**

**Sponsor:**	**Pieris AG**<br>**Lise-Meitner-Straße 30**<br>**85354 Freising**<br>**Germany**

**Amendment Version:**	**Final 1.0**
**Amendment Date:**	**28-Apr-2011**

TABLE OF CONTENTS

Page

1 OVERVIEW OF CHANGES ... 3

2 PROTOCOL SECTIONS CHANGED ... 4

2.1 Section 1 (Changes 2 and 3) ... 4

2.2 Section 10.1.3 (Change 1) ... 5

2.3 Section 11.7 (Change 3) ... 6

2.4 Section 12.2 (Change 4) ... 7

2.5 Section 12.3 (Change 3) ... 7

2.6 Section 13.1 (Changes 2 and 3) ... 7

2.7 Section 14.3 (Changes 3 and 5) ... 8

3 APPROVAL OF AMENDMENT ... 9

1 OVERVIEW OF CHANGES

The following changes to the clinical study protocol Version 2.0 are made:

Change 1:

In patients who experience infusion reactions despite the use of premedication, the duration of Angiocal® administration is extended from 20 minutes to approximately 120 minutes.

Justification:

It is likely that infusion reactions are caused by high peak plasma concentrations (C_{max}) of Angiocal®. While bolus injections result in a high C_{max}, a prolonged duration of Angiocal® administration reduces C_{max}. It is therefore anticipated that an extended duration of Angiocal® administration will prevent these infusion reactions. The prolonged infusion time does not pose any risks to the patient.

Change 2:

In patients who will receive Angiocal® over a period of 120 minutes, additional blood samples will be taken for pharmacokinetic (PK) assessments 5 minutes before the end of Angiocal® administration.

Justification:

The highest plasma levels of Angiocal® are expected at the end of administration. To gain information on the maximum tolerated C_{max}, blood samples are taken immediately before the end of Angiocal® administration.

Change 3:

Within the biweekly dosing period additional blood samples for PK, Anti-Drug Antibody (ADA), and biomarker testing will be taken pre-dose.

Justification:

The results will be used to gain more information about the pharmacokinetics and immunogenicity of Angiocal® in patients who completed the repeated dosing period and continued into the biweekly dosing schedule.

As specified in Amendment 4 to the clinical study protocol, the analysis of VEGF-A will be performed on PK sample aliquots only when these are no longer required for PK measurements. No additional blood samples will be taken for the analysis of VEGF-A.

Change 4:

Within Section 12.2 of the protocol blood sampling for routine clinical laboratory during the biweekly dosing period was not mentioned, this will be adjusted for reasons of consistency.

Change 5:

The analysis of biomarkers in plasma was erroneously deleted in Amendment 4 to the clinical study protocol. Biomarkers will be analyzed in both plasma and serum. Corresponding sections will be changed accordingly and are not further specified below.

2 PROTOCOL SECTIONS CHANGED

The protocol sections that are changed are detailed below. Bold text in the “new text” is used to indicate the addition, crossed out text in the “original text” indicates the deletion of information from the original text. Original text included previous amendments if applicable.

2.1 Section 1 (Changes 2 and 3)

Original text:

Page 11:

	Screening		Single treatment		Follow-up	
	(Day -7 to Day 0)	(Day 1) Baseline	Day 2 (24 h ±2 h)	Day 3 (48 h ±2 h)	Day 5 (96 h ±2 h)	Day 15
	Visit 1	Visit 2	Visit 3	Visit 4	Visit 5 - optional	Visit 6
…						
Blood samples for PK and VEGF-A		x[5]	x	x	x	x
…						

Page 12:

	Repeated dosing period				Final Visit	Biweekly dosing period starting on Day 57
	Day 22	Day 29	Day 36	Day 43	Day 71	
	Visit 7	Visit 8	Visit 9	Visit 10	Visit 11	
Anti-drug antibody response				x	x	
….						
….						
Blood samples for biomarkers[4]				x		
…						
…						
Blood samples for PK and VEGF-A	x	x	x	x	x	
….						
….						

At Days 1, 22, 29, 36, ~~and~~ 43 all assessments will be performed pre-dose if not otherwise noted. AEs/toxicity will be recorded pre- and post-dose.

…

[7] To be performed after every fourth dose of treatment. Treatment will stop in case of progressive disease.

…

New text:

Page 11:

	Screening		Single treatment		Follow-up	
	(Day -7 to Day 0)	(Day 1) Baseline	Day 2 (24 h ±2 h)	Day 3 (48 h ±2 h)	Day 5 (96 h ±2 h)	Day 15
	Visit 1	Visit 2	Visit 3	Visit 4	Visit 5 - optional	Visit 6
…						
Blood samples for PK[8] and VEGF-A		x[5]	x	x	x	x
…						

Page 12:

	Repeated dosing period				Final Visit	Biweekly dosing period starting on Day 57
	Day 22	Day 29	Day 36	Day 43	Day 71	
	Visit 7	Visit 8	Visit 9	Visit 10	Visit 11	
Anti-drug antibody response				x	x	**x**
….						
….						
Blood samples for biomarkers[4]				x		**x**
…						
…						
Blood samples for PK[8] and VEGF-A	x	x	x	x	x	**x**
….						
….						

At Days 1, 22, 29, 36, 43, **and during the biweekly dosing period** all assessments will be performed pre-dose if not otherwise noted. AEs/toxicity will be recorded pre- and post-dose.

...

[7] To be performed after every fourth dose of treatment. Treatment will stop in case of progressive disease.

[8] In patients who will receive Angiocal® over a period of approximately 120 minutes, PK samples will be additionally taken 5 minutes before the end of Angiocal® administration.

…

2.2 Section 10.1.3 (Change 1)

Original text:

90 minutes (±15 minutes) before each administration of Angiocal®, prophylactic treatment with clemastin (2 mg), ranitidine (50 mg), and fortecortin (16 mg) will be administered intravenously, starting with the third patient of the sixth cohort.

Angiocal® is to be administered intravenously by a slow bolus injection preferably over up to approximately 20 minutes. The volume to be administered to a patient is calculated according to handling instructions. Patients will receive 5 i.v. injections, one single treatment and 4 treatments during the repeated dosing period. During the repeated dosing period study medication will be administered once weekly for 4 weeks on Days 22, 29, 36, and 43. After each administration of Angiocal® 10 mL of a 0.9% sodium chloride solution will be administered intravenously.

New text:

90 minutes (±15 minutes) before each administration of Angiocal®, prophylactic treatment with clemastin (2 mg), ranitidine (50 mg), and fortecortin (16 mg) will be administered intravenously, starting with the third patient of the sixth cohort.

Angiocal® is to be administered intravenously by a slow bolus injection preferably over up to approximately 20 minutes. **If a patient experiences an infusion reaction after dosing, the duration of further Angiocal® administrations may be extended to approximately 120 minutes.** The volume to be administered to a patient is calculated according to handling instructions. Patients will receive 5 i.v. injections, one single treatment and 4 treatments during the repeated dosing period. During the repeated dosing period study medication will be administered once weekly for 4 weeks on Days 22, 29, 36, and 43. After each administration of Angiocal® 10 mL of a 0.9% sodium chloride solution will be administered intravenously.

2.3 Section 11.7 (Change 3)

Original text:

The investigator will decide to continue treatment during the follow-up visits every two weeks. The following assessments will take place:

- Physical examination;
- ECOG performance status;
- …

New text:

The investigator will decide to continue treatment during the follow-up visits every two weeks. The following assessments will take place:

- **Blood sampling for PK (pre-dose);**
- **Blood sampling for ADA response assessment in serum (pre-dose);**
- **Blood sampling for determination of potential cancer-associated biomarkers (pre-dose);**
- Physical examination;
- ECOG performance status;
- …

2.4 Section 12.2 (Change 4)

Original text:

Blood and urine samples for the determination of hematology, biochemistry, coagulation parameters, and urinalysis will be taken at Screening, Baseline, Day 2, Day 15 (only hematology), Day 22, Day 29, Day 36, Day 43 ~~and~~ the Final Visit.

New text:

Blood and urine samples for the determination of hematology, biochemistry, coagulation parameters, and urinalysis will be taken at Screening, Baseline, Day 2, Day 15 (only hematology), Day 22, Day 29, Day 36, Day 43**,** the Final Visit, **and during the biweekly dosing period**.

2.5 Section 12.3 (Change 3)

Original text:

Anti-drug antibodies will be analyzed by Covance Ltd., Harrogate, United Kingdom. Blood samples will be taken at Screening, on Day 43 ~~and~~ at the Final Visit on Day 71. Samples will be collected and stored at the site to be shipped to Covance in one shipment. Detailed written instructions regarding collection and storage of blood samples will be given to the investigator.

New text:

Anti-drug antibodies will be analyzed by Covance Ltd., Harrogate, United Kingdom. Blood samples will be taken at Screening, on Day 43**,** at the Final Visit on Day 71**, and during the biweekly dosing period**. Samples will be collected and stored at the site to be shipped to Covance in one shipment. Detailed written instructions regarding collection and storage of blood samples will be given to the investigator.

2.6 Section 13.1 (Changes 2 and 3)

Original text:

Pharmacokinetic samples will be taken at the following time points during the study:

- …
- Repeated dosing period: Days 22, 29, 36 and 43;
- Final Visit (Day 71)~~.~~

…

New text:

Pharmacokinetic samples will be taken at the following time points during the study:

- …
- Repeated dosing period: Days 22, 29, 36 and 43;
- Final Visit (Day 71)**;**
- **Biweekly dosing period.**

In patients who will receive Angiocal® over a period of approximately 120 minutes, PK samples will be additionally taken 5 minutes before the end of Angiocal® administration.

…

2.7 Section 14.3 (Changes 3 and 5)

Original text:

Blood samples for the analysis of potential cancer-associated biomarkers ~~in serum~~ will be taken at Day 1 (pre-dose) and Days 2, 3, 5 (optional), 15 and 43. Detailed written instructions regarding collection and storage of samples will be given to the investigator….

New text:

Blood samples for the analysis of potential cancer-associated biomarkers will be taken at Day 1 (pre-dose) and Days 2, 3, 5 (optional), 15 and 43, **and during the biweekly dosing period**. Detailed written instructions regarding collection and storage of samples will be given to the investigator….

3 APPROVAL OF AMENDMENT

Version: Final 1.0

Date: 28-Apr-2011

Study code: Pieris001

Sponsor: Pieris AG, Freising, Germany

The signatories declare, that they have read the protocol Amendment and that they agree with its contents.

Sponsor's signatory

Laurent Audoly, PhD
Chief Scientific Officer
Pieris AG
Lise-Meitner-Straße 30
85354 Freising
Germany

28 April 2011
Date

Signature

Coordinating Investigator and Director Clinical Investigation (LKP)

PD Dr. med. Klaus Mross
Klinik für Tumorbiologie an der Albert-Ludwigs-Universität Freiburg
Department of Medical Oncology
Breisacher Str. 117
76106 Freiburg im Breisgau, Germany

Date

Signature

3 APPROVAL OF AMENDMENT

Version: Final 1.0

Date: 28-Apr-2011

Study code: Pieris001

Sponsor: Pieris AG, Freising, Germany

The signatories declare, that they have read the protocol Amendment and that they agree with its contents.

Sponsor's signatory

Laurent Audoly, PhD
Chief Scientific Officer
Pieris AG
Lise-Meitner-Straße 30
85354 Freising
Germany

Date

Signature

Coordinating Investigator and Director Clinical Investigation (LKP)

PD Dr. med. Klaus Mross
Klinik für Tumorbiologie an der Albert-Ludwigs-Universität Freiburg
Department of Medical Oncology
Breisacher Str. 117
76106 Freiburg im Breisgau, Germany

29.4.11
Date

Signature

CRO's signatory

Dr. med. Edgar J. Fenzl
Managing Director/Medical Monitor
FGK Clinical Research GmbH
Heimeranstrasse 35
80339 München, Germany

2.05.2011 — Date
Edgar Fenzl — Signature

Dr. Monika Schwienbacher
Associate Project Director
FGK Clinical Research GmbH
Heimeranstrasse 35
80339 München, Germany

29 APR 2011 — Date
Monika Schwienbacher — Signature

Principal Investigator

Investigator's name (printed) | Date | Signature
